# Overexpression of RNF38 facilitates TGF-β signaling by Ubiquitinating and degrading AHNAK in hepatocellular carcinoma

**DOI:** 10.1186/s13046-019-1113-3

**Published:** 2019-03-05

**Authors:** Rui Peng, Peng-Fei Zhang, Xuan Yang, Chuan-Yuan Wei, Xiao-Yong Huang, Jia-Bin Cai, Jia-Cheng Lu, Chao Gao, Hai-Xiang Sun, Qiang Gao, Dou-Sheng Bai, Guo-Ming Shi, Ai-Wu Ke, Jia Fan

**Affiliations:** 1 Liver Cancer Institute, Zhongshan Hospital, Fudan University; Key Laboratory of Carcinogenesis and Cancer Invasion (Fudan University), Ministry of Education, 180 Feng lin Road, Shanghai, 200032 People’s Republic of China; 2 Clinical Medical College, Yangzhou University, Yangzhou, 225009 Jiangsu China; 3 Department of Oncology, Shanghai East Hospital, Tongji University School of Medicine, Shanghai, 200031 China; 4 Cancer Center, Institutes of Biomedical Sciences, Fudan University, Shanghai, 200031 People’s Republic of China

**Keywords:** Hepatocellular carcinoma, RNF38, AHNAK, TGF-β signaling, Prognosis

## Abstract

**Background:**

RING finger protein 38 (RNF38), a member of the RNF protein family, has just emerged as a vital driver of cancer progression. However, the oncogenic mechanisms of RNF38 remain unexplored.

**Methods:**

Using frozen tumor tissue and tissue microarray from hepatocellular carcinoma (HCC) patients, we tried to probe the expression of RNF38 in HCC and its clinical value. Then the biological functions of RNF38 were analyzed in vivo and vitro. Stable isotope labeling with amino acids (SILAC) in cell culture and co-immunoprecipitation proteomic analyses were combined to reveal the potential mechanism of RNF38 in HCC progression.

**Results:**

We report that RNF38 expression was markedly higher in HCC tissues than in peritumor tissues. Correspondingly, RNF38 overexpression promoted the HCC cell migration and invasion and inhibited apoptosis both in vitro and in vivo. And elevated RNF38 expression induced HCC cell epithelial-mesenchymal transition by facilitating transforming growth factor-β (TGF-β) signaling via ubiquitinating and degrading neuroblast differentiation-associated protein (AHNAK), a well-established inhibitor of TGF-β signaling. Furthermore, AHNAK interference restored the HCC cell invasion and metastasis deprived by RNF38 downregulation. Clinically, elevated RNF38 and transforming growth factor beta receptor 1 (TGFBR1) expression was related to short overall survival (OS) and high cumulative recurrence rates in HCC patients.

**Conclusions:**

High levels of RNF38 promote HCC by facilitating TGF-β signaling and are a novel marker for predicting the prognosis of HCC patients and a potential therapeutic target in HCC.

**Electronic supplementary material:**

The online version of this article (10.1186/s13046-019-1113-3) contains supplementary material, which is available to authorized users.

## Introduction

Hepatocellular carcinoma (HCC) is still among the leading causes of cancer-related death worldwide, especially in China [1, 2]. The high rate of relapse and early invasion and metastasis are the major reasons for the poor outcomes of HCC patients. Currently, except for curative surgical therapy, effective means of controlling HCC progression are lacking. During the past decade, targeted drugs such as sorafenib and regorafenib have been put into clinical use [3]. However, most patients realize a limited benefit due to drug resistance [4]. Thus, revealing the molecular mechanism of HCC progression and identifying new key markers or targets for the diagnosis and treatment of HCC patients is extremely urgent.

Ubiquitination is a complex yet significant process that controls a vast array of cellular functions, including cell metabolism, survival, differentiation, as well as the cell cycle. Among the multiple steps in the ubiquitination process, E3 ubiquitin ligases (E3s) control precise substrate recognition by mediating the transfer of ubiquitin from E2-ubiquitin-binding enzymes (E2s) to substrates, and thus govern the fate or subcellular localization of ubiquitinated proteins [5]. E3s are frequently dysregulated in tumors, resulting in the dysfunction of tumor suppressor genes or oncogenes which further participate in cellular transformation or tumor progression [6, 7]. RING finger (RNF) proteins, the largest class of E3s with RNF domains containing eight conserved cysteine and histidine residues, play various roles in human physiology and pathologies [8], especially cancer progression [9]. For example, RNF43 frequently mutates in colorectal and endometrial cancers [10] and in liver fluke-associated cholangiocarcinoma [11]. RNF38, a member of the RNF protein family, is abundant in the human testis, and its dysfunction is involved in a variety of human disorders, particularly neoplasms [12]. Indeed, the human RNF38 gene is located on the short arm of chromosome 9, which is a region enriched with drivers of chronic myelogenous leukemia (CML) [13, 14]. Additionally, RNF38 was identified to ubiquitinate TP53 in HEK293T cells [15], and upregulated RNF38 expression was recently found to promote non-small cell lung cancer cell invasion and metastasis by inducing cell epithelial-mesenchymal transition (EMT) [16]. Thus, there is a growing need to determine the detailed molecular mechanism of RNF38 in cancer.

In this study, we aimed to determine the expression of RNF38 in HCC tissues and the potential prognostic value of RNF38, particularly the definitive mechanism by which RNF38 induces the invasion and metastasis of HCC.

## Methods and materials

### Patients samples and follow up

A total of 200 HCC patients who underwent complete surgical excision between January 2006 and December 2008 at Zhongshan Hospital Fudan University (Shanghai, China) were enrolled randomly in the study. The diagnosis of HCC was confirmed independently by two pathologists. Neoplastic and matched normal tissues were obtained after surgery and were further used for tissue microarray (TMA) construction. The fresh tumor samples were acquired from Zhongshan Hospital (Shanghai, China). The study was approved by the Institutional Review Board of Zhongshan Hospital Fudan University, and informed consent was obtained from the patients prior to the study.

### Immunohistochemistry (IHC), immunoblot (IB), and real-time quantitative PCR (qRT-PCR) analyses

The IHC protocol and qualitative criteria for the intensity of staining were used as shown in our previous study [17]. And the IHC details were listed in the Additional file 1. The integrated optical density (IOD) values were assessed by Image-Pro Plus software. IB analysis was performed in our earlier study [18]. Relative protein expression was analyzed by Image J software. The information for the primary antibodies was listed in Additional file 2: Table S1. For qRT-PCR, RNA was extracted from tissue or cell lines as previously described [19], with SYBR Green Realtime PCR Master Mix (Yeasen, Shanghai, China). The PCR primers used were as follows: RNF38, forward: 5′-AACACGGAGAGCAGTTCCAC-3′ and reverse: 5′-CCTGGCATACGTCTTCAACA-3′. GAPDH, forward: 5′-GGTATGACAACGAATTTGGC-3′ and reverse: 5′-GAGCACAGGGTACTTTATTG-3′. AHNAK, forward: 5′-GAGGTCTTCCAGGCATTGGTGTTC-3′ and reverse: 5′-GGCAGGTTCA CATCACATCCAGAG-3′. TGFBR1, forward: 5′-GCAGAGCTGTGAAGCCTTGAGAG-3′ and reverse: 5′- ATGCCTTCCTGTTGACTGAGTTGC-3′.

### Cell lines and transfection

The human HCC cell lines HCCLM3, Huh7, PLC/PRF/5, and HepG2 were obtained from the Chinese Academy of Science Cell Bank (Shanghai, China). All cell lines were cultured in DMEM supplemented with 10% fetal bovine serum and 1% antibiotics at 37 °C in a humidified incubator with 5% CO_2_.

All vectors were purchased from Shanghai Genomeditech Company (Shanghai, China). The HCCLM3 cell line was transfected with RNF38 short hairpin RNA (shRNA) lentiviral vectors and the corresponding control vectors, and the HepG2 cell line was transfected with RNF38 cDNA lentiviral vectors and the corresponding control vectors. The AHNAK small interfering RNA (siRNA) was also constructed by Shanghai Genomeditech Company (Shanghai, China). The RNF38 shRNA target sequences were as follows: shRNA1: 5′- GUCUCGACAUAAUUCCAUU-3′, shRNA2: 5′-CAUGGGAGAUGACAUCAAA-3′. The AHNAK siRNA target sequences were as follows: siRNA1, forward, 5′-CUGCUGCUGCCCAACUGGC tt-3′, reverse, 5′-GCCAGUUGGGCAGCAGCAGtt-3′; siRNA2, forward, 5′-GGCGUCUUGUGC AGGAGGtt-3′, reverse, 5′-CCUCCUGCACAAAGACGCCtt-3′; siRNA3, forward, 5′-GCUGCC CAACUGGCAGGGUtt-3′, reverse, 5′ ACCCUGCCAGUUGGGCAGCtt-3′. The antibiotic-resistant transfected cells were selected by adding puromycin to the culture medium for 7 days.

### Cell migration, Matrigel invasion, and cell proliferation assays

The cell migration and matrigel invasion assays were performed as described in our previous report [20]. For the cell proliferation assay, 2 × 10^3^ cells were incubated in 96-well plates, and at the prescribed time, the OD value was determined by a cell counting kit-8 (CCK-8) assay (Yeasen, Shanghai, China).

### Immunofluorescent staining and flow cytometry assays

Immunofluorescence staining was performed as described in our previous study [21]. Flow cytometric analysis was performed to calculate the rate of HCC cell apoptosis. The cell samples were stained with annexin V-FITC/PI, cells in the lower-right quadrant were Annexin V positive and defined as early apoptotic cells, and cells in the upper-right quadrant were Annexin V positive and PI positive and defined as late apoptotic cells. Both quadrants were included to evaluate the capability of the cells to resist apoptosis.

### Tumor growth in vivo

Four-week-old nude mice were acquired from the Shanghai Institute of Material Medicine and were raised in the specific pathogen-free animal laboratory. A total of 5 × 10^6^ HCC cells were injected to generate subcutaneous tumors in the nude mice. Tumor growth was measured every 5 days after the injection until the 32nd day. Tumors were measured in two dimensions, as described in our earlier study [20]. Serial sections of mice lungs were stained with H&E, and the metastatic rate was calculated as previously described [21]. The Mice experiments conformed to the requirements of the Animal Ethics Committee of Zhongshan Hospital, Fudan University.

### Coimmunoprecipitation (co-IP)

First, cold RIPA lysis buffer (50 mM Tris (pH 7.4), 150 mM NaCl, 1% Triton X-100, 1% sodium deoxycholate, and 0.1% SDS) containing 1 μm complete protease inhibitor (Roche) was added to cultured cells. Next, the mixture was placed on a low-speed rotating shaker for 30 min at 4 °C. Then, the supernatant was transferred to new tubes after centrifugation at 13,000 g and 4 °C for 15 min. Subsequently, the precleared lysates were incubated with primary antibody (RNF38, 1:100; AHNAK, 1:100 or, IgG (homologous control antibody) 1:100) preabsorbed protein A- and G-sepharose beads overnight at 4 °C. The precipitates were cleaned with the RIPA buffer three times. The whole immunoprecipitates were boiled in 1× SDS loading buffer (Beyotime, P0015A) for 5 min, and the proteins were resolved by SDS-PAGE on 10% separating gel. Finally, immunoblots were probed with the appropriate antibody and detected by ECL.

### Stable isotope labeling by amino acids in cell culture (SILAC)

Briefly, HepG2-NC cells were maintained in normal DMEM nutrient medium with 10% FBS, which is called light from here on. And HepG2-RNF38 cells maintained with nutrient media containing L-lysine-2HCl (^13^C_6_, ^15^N_2_) (Thermo Scientific™, 88,432), which is deemed to be heavy from here on. The reverse reaction was set up under the same conditions, but HepG2-NC cells were maintained in heavy acid and HepG2-RNF38 were maintained in light acid. The whole system was cultivated in the SILAC medium for 10 passages to achieve full labeling before harvesting. The cells were lysed by modified RIPA buffer (50 mM Tris-HCl, pH 7.8, 150 mM NaCl, 1% NP-40, 0.25% sodium deoxycholate and 1 mM EDTA) [22] and mixed in a one to one ratio. The lytic cells were centrifuged in 4 °C. The supernatant was aspirated, and the mixture lysate was digested by trypsin, and fractionated by high-performance liquid chromatography (HPLC) and analyzed by LC-MS/MS. For these two reactions, we calculated the ratio of light to heavy or heavy to light peptides. And a 1.5 fold change cutoff for the peptide prophet probability used to filter the dataset. And the detail of defferential proteins are listed in Additional file 3: Table S2 and Additional file 4: Table S3

### Liquid chromatography coupled with tandem mass spectrometry (LC-MS/MS)

Immunoprecipitates or SILAC mixture was subjected to LC-MS/MS (Q-Extacive Plus, Thermo Scientific) analysis. The details of the LC-MS/MS method are described in the Additional file 1.

### Ubiquitination assay and cycloheximide (CHX) chase assay

For the ubiquitination assay, HCCLM3-NC and HCCLM3-shRNA2 cells were treated with MG132 (5 μmol, Selleck, China). Next, these two cell lines were subjected to co-IP and the IB analysis was used to detect ubiquitin. For CHX chase assay was used to observe the half-life of AHNAK. HCCLM3-NC and HCCLM3-shRNA2 cells were treated with CHX (50 μg/mL) for the indicated times, and IB was performed.

### Statistical analysis

SPSS software 21.0 was used for statistical analyses. Student’s t-test was used to compare quantitative data between two groups. The relationship between the two proteins was assessed by the Pearson correlation coefficient. The Kaplan-Meier method and the log-rank test were used to analyze the overall survival and cumulative recurrence rate. *p* < 0.05 was considered statistically significant.

## Results

### RNF38 is overexpressed in HCC tissues and associated with poor prognosis in HCC patients

**Fig. 1:**
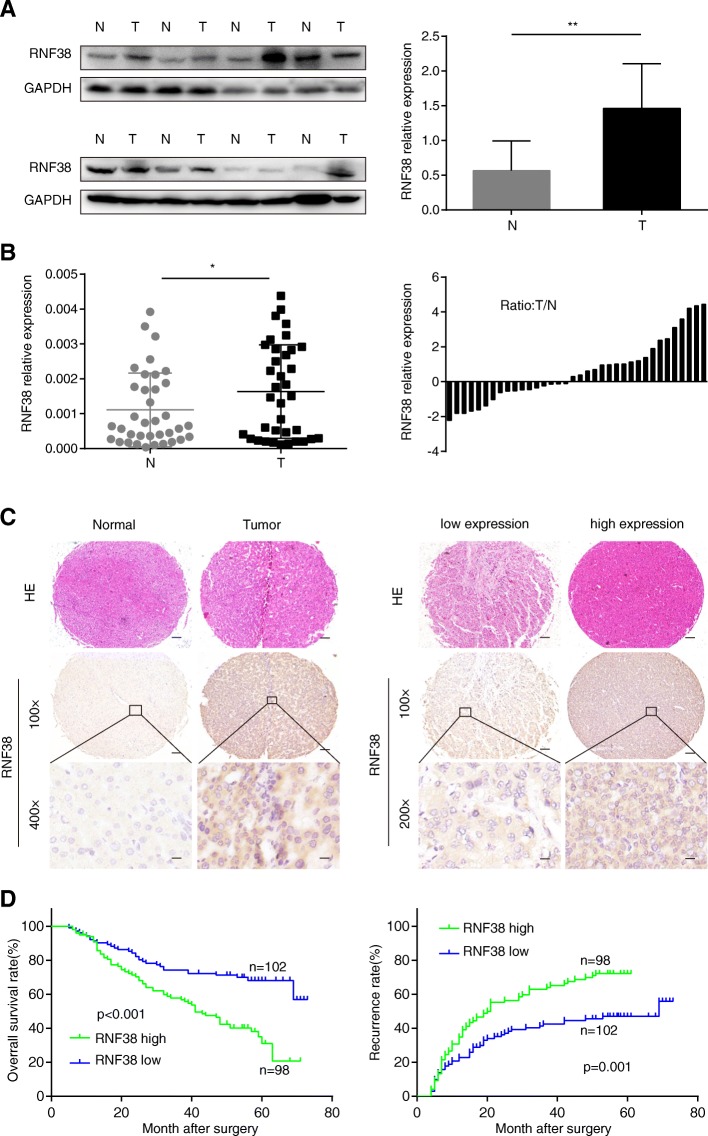
RNF38 is overexpressed in HCC patients and associated with HCC patients’ poor prognosis. **a**. The expression of RNF38 protein in HCC tumor tissues and matched normal tissues was verified by western blot. **b**. The expression of RNF38 mRNA was verified by qRT-PCR. **c**. The expression of RNF38 was measured by IHC in TMAs including 200 HCC patients, and typical photos were presented. **d**. Prognostic analysis of RNF38 expression in 200 HCC patients. Scale bar: 100 μm. *,*p* < 0.05

We first examined the levels of RNF38 protein and mRNA in 36 pairs of HCC and adjacent nontumor tissues and found that the RNF38 protein expression was higher in HCC tissues than in normal tissues (Fig. 1a, *p* = 0.033). This finding was also true for the mRNA expression (Fig. 1b, *p* = 0.036). These results were consistent with those availabe in a public database (www.oncomine.org), that showed that RNF38 was overexpressed in several gastrointestinal cancers (Additional file 5: Figure S1 A-D). IHC staining of tissues from 200 HCC patients showed that RNF38 expression was significantly higher in tumors than in peritumor tissues (Fig. 1c, left panel). Moreover, the RNF38 expression in HCC samples was considerably variable among patients (Fig. 1c, right panel). Further analyses indicated that high levels of RNF38 expression were significantly correlated with large tumor size (*p* = 0.008), the absence of tumor encapsulation (*p* = 0.004), the presence of embolus (*p* = 0.030), and advanced TNM stage (*p* = 0.019, Table 1). Moreover, patients with high RNF38 expression (*n* = 98) had shorter survival times and a high rate of recurrence than those with low RNF38 expression (*n* = 102, Fig. 1e). Importantly, Cox regression analysis indicated that tumor size, embolus, and the RNF38 staining level are independent prognostic factors for HCC patients (Table 2**)**. All in all, these results suggest that RNF38 could be a key promoter of HCC progression.

**Table 1:** Correlation between RNF38 and clinicopathological characteristics in 200 HCC patients

Variables	Number of patients		
	*RNF38* ^*high*^	RNF38^low^	*P* value
Gender			
Female	12	15	0.611
Male	86	87	
Year			
< 52	43	53	0.253
≥ 52	55	49	
Hepatic cirrhosis			
Yes	87	91	0.921
No	11	11	
HbsAg			
Positive	79	86	0.491
Negtive	19	16	
HCV			
Positive	0	2	0.498^a^
Negtive	98	100	
AFP			
< 20	*33*	43	0.217
≥ 20	*65*	59	
Tumor size (cm)			
< 5	46	67	0.008 (**)
≥ 5	52	35	
Tumor number			
Single	83	85	0.793
Multiple	15	17	
Tumor encapsulation			
Complete	61	43	0.004 (**)
None	37	59	
Tumor differentiation			
I + II	66	79	0.110
III + IV	32	23	
Edmondson-Steiner Grade			
I + II	58	62	0.817
III	40	40	
Embolus			
Absence	60	77	0.030 (*)
Present	38	25	
TNM stage			
I	61	79	0.019 (*)
II + III	37	23	

Note: ^a^, Fisher test; AFP, alpha-fetoprotein; *p* < 0.05 was considered significant; **p < 0.01; *p < 0.05

**Table 2:** Univariate and multivariate analyses of factors associated with overall survival and cumulative recurrence

Factors	overall survival				cumulative recurrence			
	Univariate		Mulvariate		Univariate		Mulvariate	
	*P* value	HR	95%Cl	*P* value	*P* value	HR	95% Cl	*P* value
Gender (Female vs. Male)	0.060			NA	0.127			NA
Age (years) (≥53 vs. < 53)	0.888			NA	0.469			NA
Liver cirrhosis (yes vs. no)	0.930			NA	0.127			NA
HBsAg (positive vs. negative)	0.183			NA	0.622			NA
HCV (positive vs. negative)	0.983			NA	0.793			NA
Serum AFP, ng/mL (≥20 vs. < 20)	0.065			NA	0.011			NS
Tumor differentiation (III/IV vs. I/II)	0.197			NA	0.069			NA
Edmondson-Steiner Grade (I/II vs. III)	0.191			NA	0.018			NS
Tumor encapsulation (yes vs. no)	0.195			NA	0.043			NS
TNM stage (I/II vs. III/IV)	0.005			NS	0.086			NA
Tumor size (diameter, cm) (≥5 vs. < 5)	0.001	1.587	1.043–2.414	*0.031*	0.001	1.823	1.247–2.665	*0.002*
Embolus (Absence vs. Present)	< 0.001	2.232	1.472–3.385	*< 0.001*	< 0.001			NS
Tumor number (multiple vs. single)	0.045			NS	0.002	2.121	1.366–3.294	*0.001*
RNF38 staining (high vs. low)	< 0.001	2.046	1.321–3.171	*0.001*	0.001	1.642	1.117–2.415	*0.012*

Abbreviations: *95% CI* 95% confidence interval, *AFP* alpha-fetoprotein, *TNM* tumor node metastasis, *HBsAg* hepatitis B surface antigen, *HCV* hepatitis C virus, *HR* hazard ratio, *NA* not adopted, *NS* not significant

Cox proportional hazards regression model

### High levels of RNF38 promote HCC cell motility, and invasion and inhibit HCC cell apoptosis both in vitro and in vivo

**Fig. 2:**
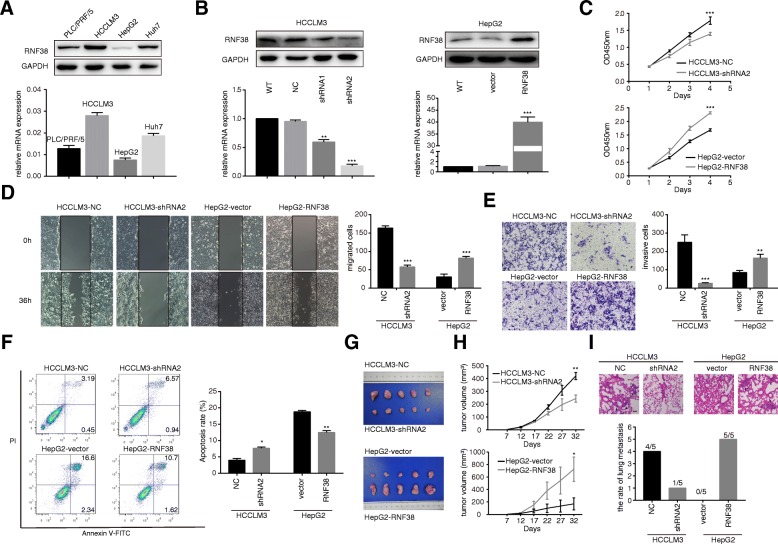
RNF38 involved in tumor motility and invasiveness, apoptosis and proliferation in vitro and *vivo*. **a**. Western blot detected RNF38 expression in four HCC cell lines (PLC/PRF/5, HCCLM3, HepG2, Huh7). **b**. The efficiency of transfection in HCCLM3 and HepG2 cell lines were confirmed by qRT-PCR and western blot. **c**. Cell proliferation was measured by the CCK-8 assay. **d**. Cell migration in HCC cells with different RNF38 expression was assessed by wound healing assay. **e**. Cell invasion ability in HCC cells was assessed by transwell assay. **f**. HCC cells apoptosis rate was evaluated by flow cytometry. **g**. Tumor derived from nude mice implanted with the HCCLM3-NC, HCCLM3-shRNA2, HepG2-vector, HepG2-RNF38 cell lines (*n* = 5). **h**. Tumor growth curves of the subcutaneous xenografts. **I.** The representative photos showed lung metastasis. Scale bar: 100 um.* *p* < 0.05, ** *p* < 0.01, *** *p* < 0.001

To understand the effect of RNF38 on HCC development, we determined the RNF38 expression in four HCC cell lines (Fig. 2a) and transfected RNF38 shRNA into HCCLM3 cells and RNF38 vectors into HepG2 cells. Stable expression of RNF38 was confirmed by western blotting and qRT-PCR (Fig. 2b). The CCK-8 assay revealed that RNF38 knockdown inhibited the HCC cell growth rate (Fig. 2c, both *p* < 0.001,). Moreover, the migration and invasion assays showed that overexpression of RNF38 promoted the motility of HCC cells (Fig. 2d, *p* < 0.001 and Fig. 2e, *p* < 0.001, *p* = 0.0046). Furthermore, flow cytometry indicated that RNF38 downregulation enhanced the rate of apoptosis inhibition (Fig. 2f, *p* = 0.0121, *p* = 0.0076). Subsequently, a subcutaneous xenograft model was established to analyze the function of RNF38 in vivo. One week after inoculation, tumors were palpable, and the growth curve suggested that HCCLM3-NC cells (398.32 ± 16.85 mm^3^) exhibited higher proliferation ability than HCCLM3-shRNA2 cells (217.79 ± 19.69 mm^3^). In contrast, HepG2-RNF38 cells group (671.43 ± 107.14 mm^3^) had a higher proliferative ability than HepG2-NC cells (170.43 ± 37.96 mm^3^, Fig. 2g and h). Moreover, the lung metastasis rate was examined by H&E staining (Fig. 2i, upper panel), and the results showed that the Incidence of pulmonary metastasis was 80% (4 of 5) in the group of animals injected with HCCLM3-NC cells, which markedly higher than that in the HCCLM3-shRNA2 group (20%, 1 of 5). Furthermore, the metastasis rate was 0% (0 of 5) in HepG2-NC group, and 100% (5 of 5) in the HepG2-RNF38 group. (Fig. 2i, lower panel).

### High levels of RNF38 induce EMT in HCC cells

**Fig. 3:**
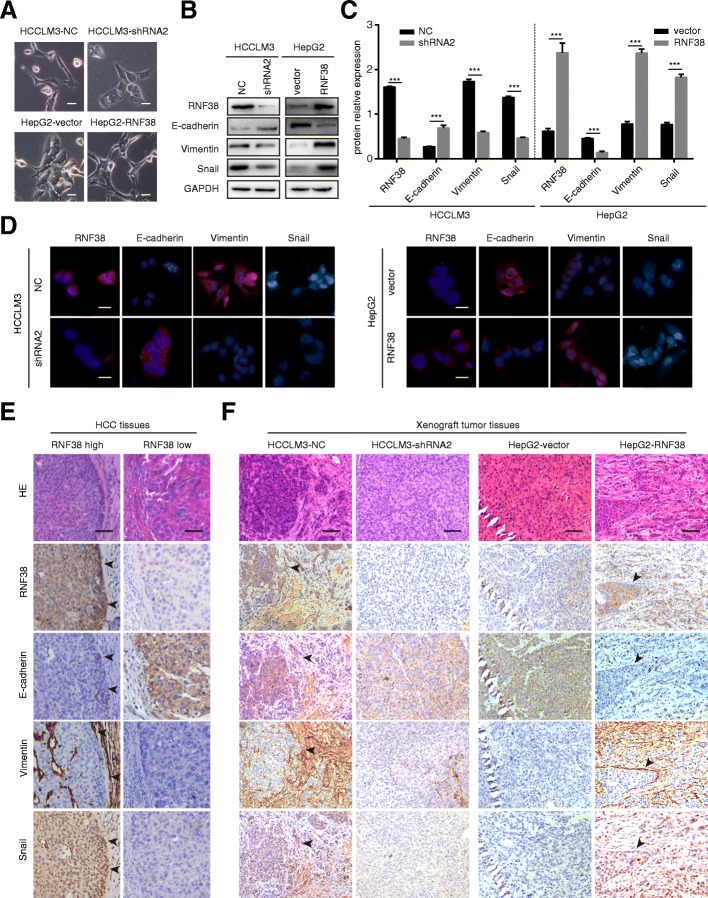
RNF38 induced HCC cell EMT. **a**. The cellular morphology of four cell lines (HCCLM3-NC, HCCLM3-shRNA2, HepG2-vector, and HepG2-RNF38) was detected by phase contrast microscope. **b**. Expression of epithelial and mesenchymal markers was compared among HCCLM3-NC, HCCLM3-shRNA2, HepG2-vector, and HepG2-RNF388 cell lines. **c**. Semi-quantitative analysis of western bolt. **d**. Representative immunofluorescent images of RNF38, E-cadherin, Vimentin, and β-catenin in HCCLM3-NC, HCCLM3-shRNA2, HepG2-vector, and HepG2-RNF38 cells. **e**. Serial section and IHC staining showed expression of RNF38, E-cadherin, Vimenail. **f**. Xenograft tumor tissues IHC staining showed expression of RNF38, E-cadherin, Vimentin, and Snail. Scale bars 50 μm. The typical EMT cells are represented as black arrows. * *p* < 0.05,** *p* < 0.01, *** *p* < 0.001

The role of EMT in cancer metastasis is now widely accepted [23]. Here, we found that HCCLM3-shRNA2 and HepG2-NC cells joined closely like epithelial cells, while the other two groups of cells appeared spindle-like, and fibroblastic (Fig. 3a). Next, we determined the levels of several EMT markers in cell lines with different RNF38 expression levels and found that the E-cadherin expression was higher in HCCLM3-shRNA2 cells than in HCCLM3-NC cells and in HepG2-NC cells than in HepG2-RNF38 cells, while the expression of snail and vimentin was lower in HCCLM3-shRNA2 cells than in HCCLM3-NC cells and in HepG2-NC cells than in HepG2-RNF38 cells (Fig. 3b). Semi-quantitative IB analysis results are shown in Fig. 3c. Furthermore, The EMT phenotype was further confirmed by immunofluorescence (Fig. 3D). Importantly, IHC analysis of the serial sections showed that cancer cells from aggressive margins had higher levels of RNF38, vimentin and snail and loss of E-cadherin in both HCC and transplanted tumor tissues, strongly suggesting that cells with elevated RNF38 expression underwent EMT (Fig. 3e and f). The above results indicate that RNF38 fosters the progression of HCC by inducing EMT in HCC cells.

### AHNAK is a substrate of RNF38 in HCC cells

**Fig. 4:**
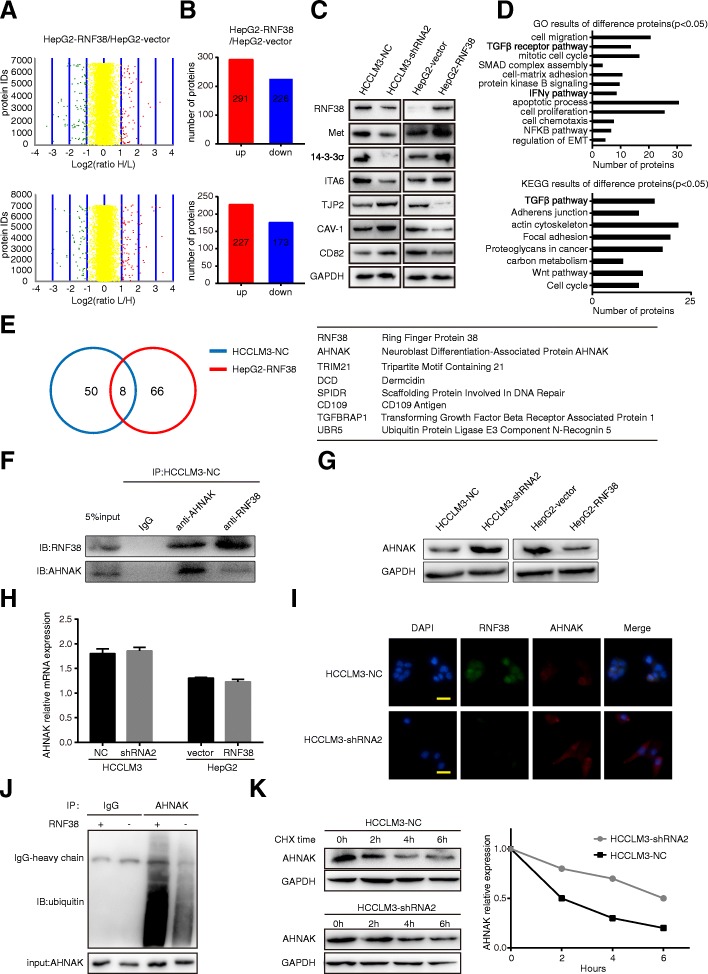
Proteomics analysis indicated AHNAK is a ubiquitination substrate of RNF38. **a**. SILAC-based proteomics data of HepG2-vector and HepG2-RNF38 cells. Red plots represent up-regulated proteins and green plots represent down-regulated proteins. **b**. Histogram showed the total number of up-regulated proteins and down-regulated proteins in two groups. **c**. Randomly chose eight proteins to verify the proteomics data. **d**. GO and KEGG analysis were performed for those up or down regulated proteins. **e**. Venn chart showed the number of substrates of RNF38 between HCCLM3-NC and HepG2-RNF38, and 8 overlapped proteins were included in the diagram. Eight overlapped proteins are listed in the Table. **f**. Co-IP and western blot were used to definite the RNF38 interacted with AHNAK. **g**. Western blot revealed that RNF38 expression affected the level of AHNAK protein. **h**. qRT-PCR showed that RNF38 had no influence on the AHNAK mRNA. **i**. Immunofluorescent staining images showed that the high level of RNF38 (green) was negatively related to the AHNAK (red) protein level. Nucleus (blue). **j**. AHNAK were immunoprecipitated from HCCLM3-NC and HCCLM3-shRNA2 and analyzed by ubiquitin antibody; *NC* negative control, *KD* knockdown. **k**. AHNAK’s half-life was prolonged after RNF38 de-regulation. CHX treated time point is 0 h, 2 h, 4 h, 6 h, respectively. And relative AHNAK expression was shown in the right panel. Abbreviations: CHX, cycloheximide; DAPI, 4′, 6-diamidino-2-phenylindole. Scale bars 50 um

Here, we performed SILAC to determine the change in total protein in cells with different RNF38 expression levels (Fig. 4a) and found that 518 proteins were upregulated, and 399 proteins were downregulated (Fig. 4b). These results were further confirmed by selecting 8 proteins randomly for western blot analysis (Fig. 4c). The functional analysis indicated that the differentially expressed proteins were enriched primarily in the Gene Ontology (GO) terms of cell migration, transforming growth factor beta receptor pathway, cell cycle, cell proliferation, negative regulation of apoptotic process, cell chemotaxis, and positive regulation of epithelial to mesenchymal transition, and the Kyoto Encyclopedia of Genes and Genomes (KEGG) pathways of cell cycle, central carbon metabolism in cancer, Wnt pathway, proteoglycans in cancer, focal adhesion, regulation of actin cytoskeleton, adherens junction, and TGF-β pathway (Fig. 4d). Then, co-IP was used to isolate and identify the interactome of RNF38 in protein lysate from HCC cells expressing high levels of RNF38. In this assay, 58 and 74 proteins were isolated from HCCLM3-NC and HepG2-RNF38 cell lines, respectively (Fig. 4e). In addition, eight proteins including RNF38, AHNAK, tripartite motif containing 21 (TRIM21), dermcidin (DCD), scaffold protein involved in DNA repair (SPIDR), CD109 molecule (CD109), transforming growth factor beta receptor associated protein 1(TGFBRAP1), ubiquitin protein ligase E3 component n-recognin 5(UBR5) overlapped in the two different cell lines (Fig. 4e). Interestingly, TRIM21 and UBR5 are E3 ligases [24, 25], CD109 and TGFBRAP1 are well-known regulators in the TGF-β pathway that act as oncogenes [26, 27]. Due to the obvious downregulation of AHNAK in cells overexpressing RNF38, we tried to elucidate the relationship between RNF38 and AHNAK protein in HCC cell. Co-IP combined with immunoblotting showed that endogenous RNF38 forms a complex with AHNAK (Fig. 4f) and immunoblotting revealed that although the overexpression of RNF38 resulted in the downregulation of AHNAK protein expression (Fig. 4g), RNF38 expression was not related to AHNAK mRNA expression (Fig. 4h). Additionally, IF analysis showed that the high level of RNF38 was negatively associated with the AHNAK protein level (Fig. 4i). A a ubiquitination assay and a CHX chase assay, indicated a decrease of RNF38 that obviously abrogated the AHNAK ubiquitination after MG132 (5 μmol) treatment for 10 h (Fig. 4j). Furthermore, the physical interaction between RNF38 and AHNAK was examined, the confocal imaging showed that RNF38 (green) and AHNAK (red), whose expression partially overlapping, indicated that they interacted with each other (Additional file 6: Figure S2). Furthermore, the downregulation of RNF38 prolonged the half-life of the AHNAK protein (Fig. 4k). These data suggest that the AHNAK protein is a substrate of RNF38 and that RNF38 promotes AHNAK degradation.

### RNF38 facilitates TGF-β/Smad2/3 signaling in HCC cells by degrading AHNAK protein

**Fig. 5:**
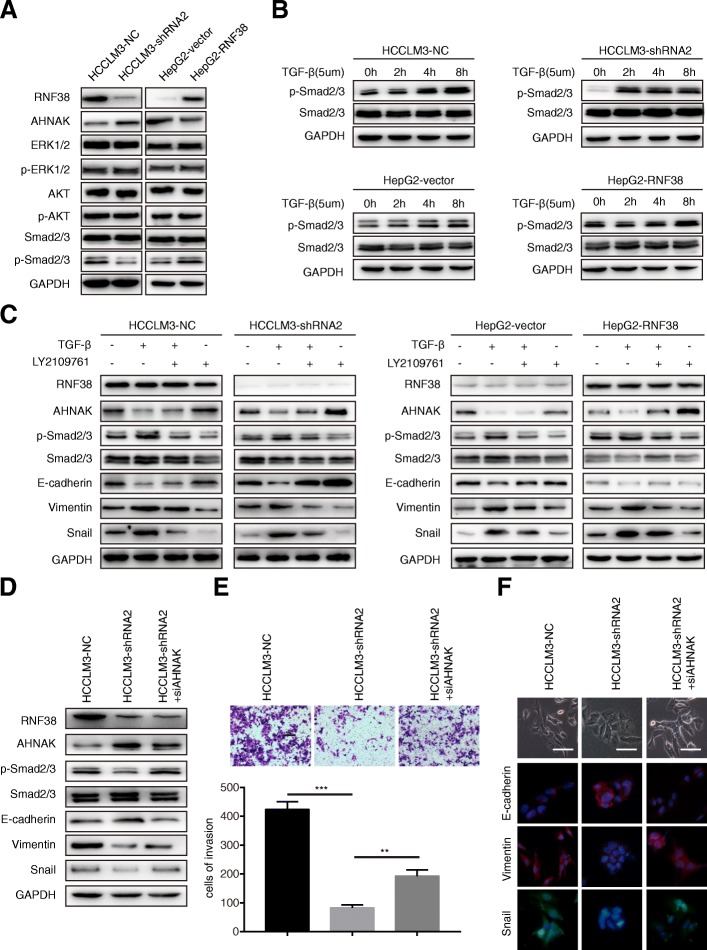
High level of RNF38 faciliates TGF-β pathway by ubiquitinating and degrading AHNAK. **a**. Western blot showed the phosphorylation level of ERK1/2, AKT, Smad2/3 when the RNF38 was knockdown or overexpression. **b**. Western blot showed phosphorylation level of Smad2/3 in HCCLM3-NC, HCCLM3-shRNA2, HepG2-vector and HepG2-RNF38 cells treated by (TGF-β, 5um) for 0 h, 2 h, 4 h, 8 h. **c**. The levels of main EMT molecules and p-Smad after TGF-β and/or inhibitor incubation. **d**. The levels of main EMT molecules and p-Smad in HCCLM3 co-transfected NC, shRNF38, siAHNAK. **e**. Cell migration and invasion of HCCLM3 was inhibited by shRNF38, which was further rescued by AHNAK suppression. **f**. The cell EMT was inhibited via knockdown RNF38, and rescued by the consequential downregulation of AHNAK in HCCLM3 cells. The shape of cancer cells and EMT markers were showed. Scale bars 100 um

**Fig. 6:**
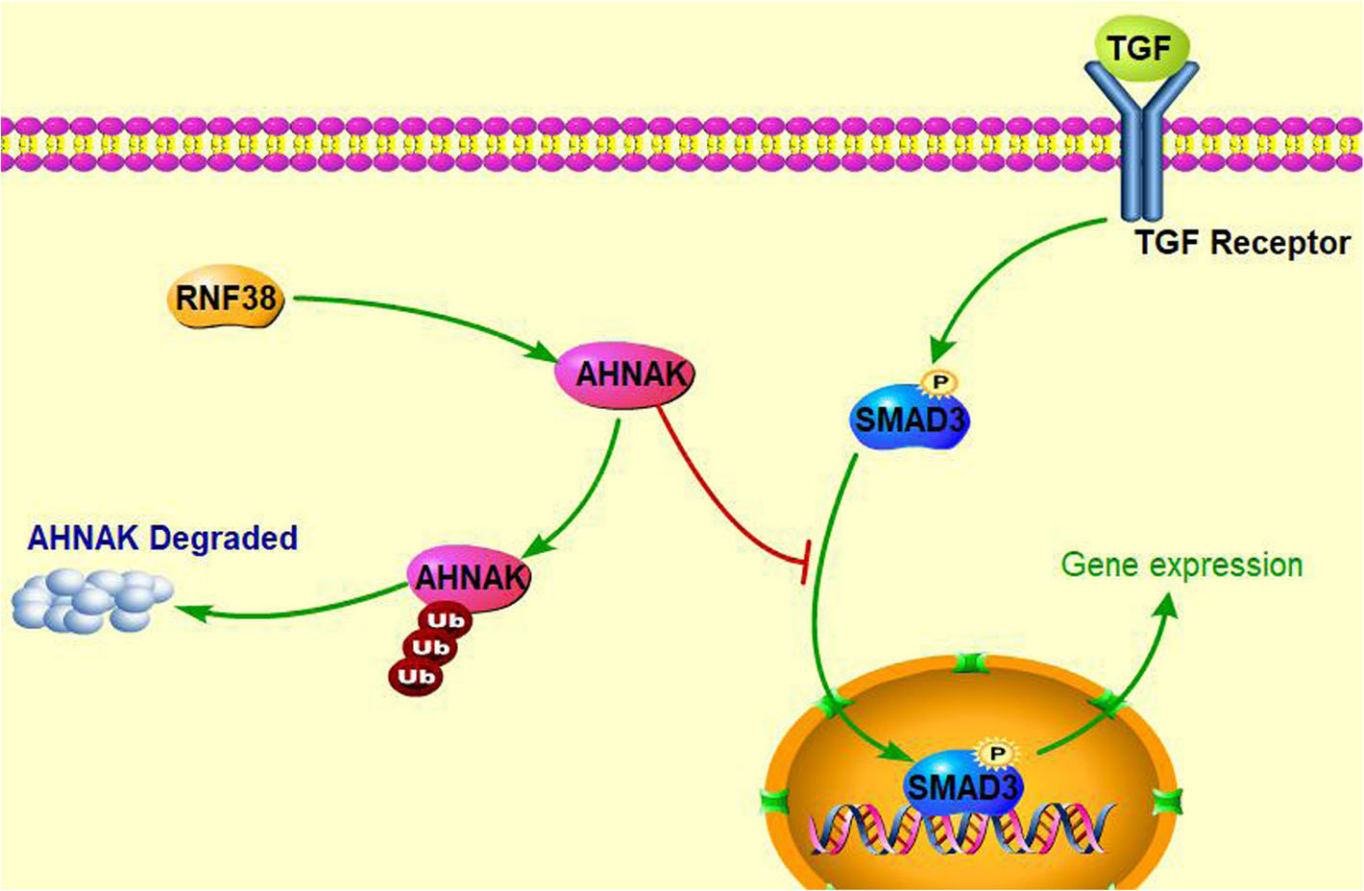
Model of high level of RNF38 on TGF-β/Smad signaling. RNF38 overexpression ubiquitinate and degrade AHNAK, which inhibit the p-Smad enteintor the nucleus

The SILAC results indicated that the RNF38 might be involved in the TGF-β pathway, and previous studies have demonstrated that AHNAK could influence several signaling pathways, especially the TGF-β pathway [28–30]. Here, we tried to determine the signaling related to RNF38 expression. As shown in Fig. 5a**,** the expression of the phosphorylated Drosophila mothers against decapentaplegic protein 2/3 (p-Smad2/3) was reduced by RNF38 knockdown, but there were no significant changes in the expression of phosphorylated AKT or ERK1/2, indicating that a high level of RNF38 might promote HCC progression mainly via TGF-β1/Smad signaling. Thus, we further determined the role of RNF38 in TGF-β pathway. After treating four cell lines with TGF-β1 (5 μm) for 0 h, 2 h, 4 h, 8 h, we chose the 8 h as the optimal treatment time for the subsequent study (Fig. 5b). The four cell lines were treated with TGF-β1 (5 μm, 8 h) and/or LY2109761 (10 μm, 2 h), a TGF-β/smad2/3 inhibitor, and we found that compared with the control cells, RNF38 silenced cells showed a decreasing trend in the response to TGF-β stimulation, which was rescued by LY2109761 treatment (Fig. 5c). Additionally, to assess whether an increase of AHNAK protein expression by RNF38 knockdown also led to decreased phospho-Smad2/3 levels, we reintroduced siAHNAK in HCCLM3-shRNA2 and compared the phosphorylation level of Smad2/3 among in HCCLM3-NC, HCCLM3-shRNA2, and HCCLM3-shRNA2-siAHNAK cells (Additional file 7: Figure S3). Immunoblotting showed that interference of AHNAK restored phospho-smad2/3 levels in RNF38-depleted cells (Fig. 5d). Importantly, the functional test revealed that suppressing AHNAK in HCCLM3-shRNA2 cells dramatically restored HCC cell invasiveness (Fig. 5e) and remodeled the cell morphology from epithelial cells to mesenchymal cells (Fig. 5f). These results imply that RNF38 contributes to the invasion and EMT of HCC cells by ubiquitinating and degrading AHNAK to abrogate the inhibition of TGF-β signaling (Fig. 6).

### RNF38 expression and TGF-β signaling in the prognosis of HCC patients

**Fig. 7:**
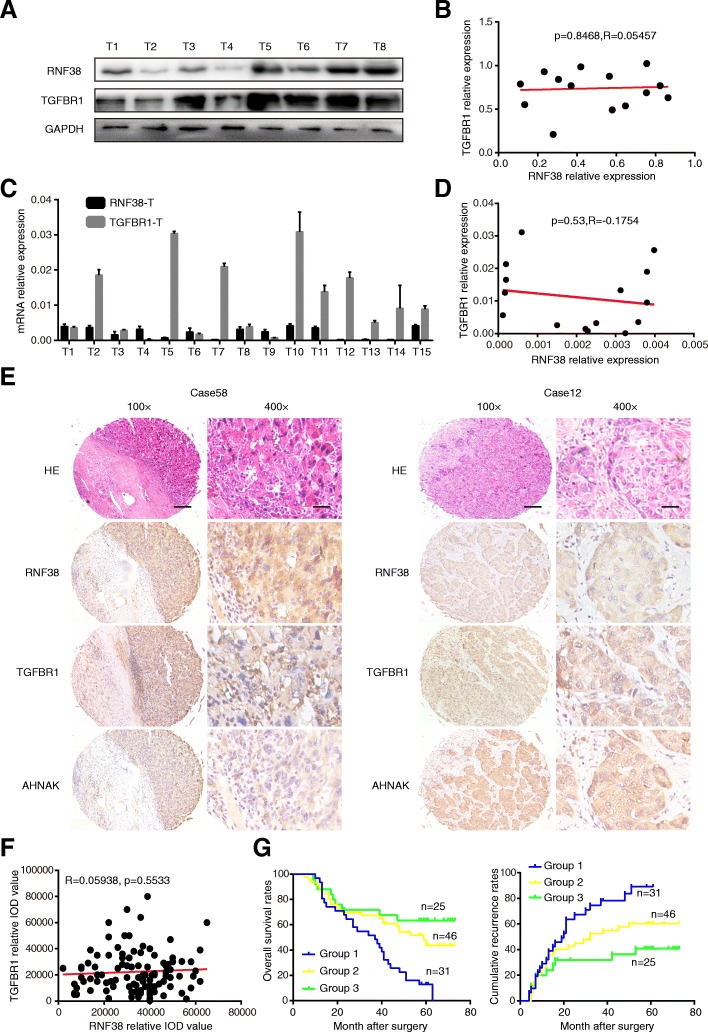
The RNF38/TGFBR1 complex is an independent factor predicting the prognosis of HCC patients. **a**. The protein expression of RNF38 and TGFBR1 in 15 HCC tumor tissue were detected through western blot, and the representative images were shown. **b**. A positive correlation between two proteins were observed in tumor tissues at the protein. **c**. The mRNA expression of RNF38 and TGFBR1 in 15 HCC tumor tissue were detected through qRT-PCR. The representative image was shown. **d**. there is no correlation between the two proteins were detected in tumor tissues at the mRNA level. **e**. Representative immunostaining images of RNF38, AHNAK and TGFBR1. **f**. Percentage of specimens showing low or high expression of TGFBR1 in 102 HCC tissues, respectively, with low and high of RNF38 expression. And their relationship was assessed by Pearson correlation coefficient **g**. HCC patients with high RNF38 and high TGFBR1 had the most unfavorable prognosis including the OS and recurrence. Group 1, patients of RNF38 high and TGFBR1 high (*n* = 31). Group 2, patients of RNF38 and TGFBR1 low or high (*n* = 46), Group 3, patients of RNF38 low and TGFBR1 low (*n* = 25)

In most cell types, the canonical TGF-β pathway induces Smad phosphorylation via TGFBR1, which is activated by TGFBR2 upon ligand binding and forms a tetrameric receptor heterocomplex with TGFBR2 [31]. To further reveal the role of RNF38 in TGF-β signaling in HCC patients, we randomly selected 15 HCC tissues in which determine the expression of RNF38 and TGFBR1, an important molecule in the TGF-β pathway, at the protein and mRNA level (Fig. 7a and c), and the Pearson correlational analysis showed that no correlation between RNF38 and TGFBR1 expression at either the protein (*R* = 0.1006, *p* = 0.72; Fig. 7b) or mRNA level (*R* = -0.1754, *p* = 0.53; Fig. 7d). Moreover, we randomly chose the 102 of 200 patients and validated the expression of RNF38, TGFBR1, and AHNAK by IHC. The results revealed that both RNF38 and AHNAK were present in the cytoplasm of HCC cells, and that positive TGFBR1 staining was mainly localized in the membrane and cytoplasm of cancer cells (Fig. 7e). The correlation between RNF38 and TGFBR1 protein expression in these patients was analyzed via a scatter plot, the results suggested that there was no significant correlation between RNF38 and TGFBR1 expression (*p* = 0.55, R = 0.05938, Fig. 7F). Unexpectedly, patients with high levels of both RNF38 and TGFBR1 exhibited a trend of poor prognoses (Group 1, patients with both RNF38 and TGFBR1 high (*n* = 31). Group 2, patients with either RNF38 or TGFBR1 high (*n* = 46), Group 3, patients with both RNF38 and TGFBR1 low (*n* = 25). (Fig. 7g). In addition, the expression of RNF38 significantly correlated with the expression of AHNAK (Additional file 8: Figure S4A). The scatter plot revealed a significant inverse correlation between the relative IOD values for RNF38 and AHNAK (Additional file 8: Figure S4B), indicating that the higher the RNF38 expression was, the lower AHNAK density. Furthermore, the 5-year OS and recurrence curve indicated that the AHNAK-high group (*n* = 54) had a higher rate of survival and a lower rate of recurrence than the AHNAK-low group (*n* = 48). (*p* = 0.02, *p* = 0.03, respectively. Additional file 8: Figure S4B). Additionally, we divided the patients into three groups according to positive staining intensity in our previous study [20]: Group 1 comprised patients with low RNF38 and high AHNAK (*n* = 30). Group 2, patients with RNF38 and AHNAK expression either both low or both high, Group 3, patients with high RNF38 and low AHNAK expression (*n* = 36). Then, we investigated the impact of combined RNF38 and AHNAK expression on survival and recurrence. Group 3 had the lowest overall survival rate (33.9% vs 67.5 and 83.4%) and highest recurrence rate (75.9% vs 70.5 and 39.0%) than others group (33.9% vs 67.5 and 83.4%, Additional file 8: Figure S4C and 4D). In conclusion, co-expression of RNF38 and TGFBR1 is an independent prognostic parameter for OS and cumulative recurrence.

## Discussion

HCC is a cancer with high invasion and metastasis potential, which contributes to the low survival rate of HCC patients. In the present study, we identified RNF38 as a novel driver in HCC progression via the induction of EMT. First, we discovered that RNF38 expression was elevated in HCC tissues and that a high level of RNF38 was positively associated with the HCC malignant phenotype in a large sample of clinical specimens. Next, we revealed via an experimental approach combining SILAC and co-IP that RNF38, an E3 ligase, regulated AHNAK protein expression. Moreover, we demonstrated that high levels of RNF38 powerfully regulate TGF-β signaling. Finally, we showed that RNF38 combined with TGFBR1 expression was an independent predictor of recurrence and survival in HCC patients. The intricate signaling network orchestrated by TGF-β signaling to modulate the different stages of HCC indicated that RNF38 might play a unique role in the molecular pathogenesis of HCC.

Here, we provided sufficient evidence to demonstrate that RNF38 promotes HCC progression; notably, cells with high levels of RNF38 tended to exhibit the mesenchymal phenotype both in vitro and in vivo. Combined with the report that the mutation of RNF38 has been found in hepatitis B virus-positive HCC cells based on a cDNA microarray assay [32], our results indicate that RNF38 might promote HCC progression via diverse mechanisms. Furthermore, as an E3 ligase, RNF38 may have complex biological functions due to its diverse substrates. We combined SILAC and co-IP with transcriptome analysis to show that RNF38 functions by the ubiquitylation of AHNAK, which is a protein belonging to the AHNAK family that consists of two giant proteins [33]. Although AHNAK was originally revealed to be involved in promyelocytic leukemia [34], it is currently known to be related to solid tumor development. For instance, AHNAK negatively regulates cell growth via TGF-β signaling as a tumor suppressor in breast cancer [28]. Recently, a report suggested that the knockdown of AHNAK in a melanoma cell line led to the loss of cadherin-1 and was associated with poor patient outcomes [29], providing evidence that upregulated RNF38 induced a reduction in E-cadherin levels rather than directly interacting with E-cadherin. The results of the present study revealed that AHNAK acts as a tumor suppressor that is ubiquitinated by RNF38 at the protein level but is not affected at the mRNA level. These findings offer novel insight into the inactivation of this tumor suppressor. Furthermore, the correlation analysis indicated a strong negative correlation between RNF38 and AHNAK protein levels in tumor tissues from HCC patients. Clinically, the RNF38/AHNAK complex predicted poor outcomes of HCC patients. Thus, we concluded that a high level of RNF38 might strongly promote HCC.

The TGF-β evokes an intricate signaling network in HCC progression [35], and the dysfunction of this signaling network leads to early-stage HCC [36]. A dual character of the TGF-β pathway in tumors has long been recognized, and a deep comprehension of the mechanistic basis and clinical relevance of TGF-β signaling is required. Here, using RNA interference and pharmacologic approaches, we provided solid evidence that the overexpression of RNF38 contributes to HCC cell EMT through facilitating TGF-β signaling by the ubiquitination of AHNAK. Thus, we identified a novel player in TGF-β signaling that disables the tumor-suppressive arm of this pathway. Indeed, we consistently found that RNF38 interference downregulated Smad2/3 phosphorylation to inhibit cellular responses to TGF-β1. By combining these findings with those of studies indicating the tumor-promoting effects of increased RNF38 expression in clinical and animal models, we conclude that the level of RNF38 modulates cell responsiveness to TGF-β, thus playing an important role in HCC development.

## Conclusion

RNF38 is overexpressed in HCC, and RNF38 upregulation endowed HCC cells with the EMT phenotype by facilitating TGF-β signaling via ubiquitinating and degrading AHNAK. Therefore, RNF38 could be a novel marker for poor prognosis in HCC patients and a potential therapeutic strategy for HCC.

## Additional files

Additional file 1: Supplementary methods and materials and figure legends. (DOCX 19 kb)

Additional file 2: **Table S1.** Antibodies resource (DOCX 15 kb)

Additional file 3: **Figure S1.** Analysis RNF38 mRNA level in gastrointestinal tumor vs. normal tissues. **A**-**D**. RNF38 mRNA level was up-regulated in colon cancer, gastric cancer, and liver cancer, compared to normal tissues (All data retrieved from www.oncomine.org). (PDF 114 kb)

Additional file 4: **Figure S2.** Confocal microscopy images of RNF38 and AHNAK. Shown are HCCLM3-NC cells (blue, DAPI; green, RNF38; red, AHNAK). Scale: 2000 × . (PDF 113 kb)

Additional file 5: **Figure S3.** The AHNAK expression in HCCLM3 and HCCLM3-siAHNAK cells. Western blot showed that the expression of AHNAK after transfecting the short interfering RNA to HCCLM3. (PDF 152 kb)

Additional file 6: **Figure S4.** Clinical relevance of RNF38 and/or AHNAK expression. **A.** The correlation between RNF38 and AHNAK were assessed by the Pearson correlation. **B.** RNF38 and AHNAK IOD relative value of 102 patients had negative correlation. **C.** HCC patients with low AHNAK had the lowest survival rate and the highest recurrence rate. **D.** HCC patients with high RNF38 and low AHNAK had the most unfavorable prognosis including the OS and recurrence. Group 1, patients of RNF38 low and AHNAK high (*n* = 30). Group 2, patients of both RNF38 and AHNAK low or high (*n* = 36), Group 3, patients of RNF38 high and AHNAK low (*n* = 36). (PDF 132 kb)

Additional file 7: **Table S2.** SILAC of differential proteins in HepG2-RNF38 and HepG2-vector (Up-regulated) (DOCX 43 kb)

Additional file 8: **Table S3.** SILAC of differential proteins in HepG2-RNF38 and HepG2-vector (Down-regulated) (DOCX 37 kb)

## Abbreviations

AHNAK: Neuroblast Differentiation-Associated Protein
CML: Chronic myelogenous leukemia;
EMT: Epithelial-mesenchymal transition
HCC: Hepatocellular carcinoma
OS: Overall survival
qRT-PCR: Real-Time Quantitative polymerase chain reaction
RNF: RING finger protein
SILAC: Stable isotope labeling with amino acids in cell culture
Smad: Drosophila mothers against decapentaplegic protein
TGF-β: Transforming growth factor-β
TMA: Tissue microarray
