# Supplementary material for: Overexpression of RNF38 facilitates TGF-β signaling by Ubiquitinating and degrading AHNAK in hepatocellular carcinoma
Source: J Exp Clin Cancer Res. 2019 Mar 5;38:113. doi: 10.1186/s13046-019-1113-3 (PMC6402116; doi:10.1186/s13046-019-1113-3)
Supplement: Supplementary file 1 — Supplementary methods and materials and figure legends. (DOCX 19 kb) [file 13046_2019_1113_MOESM1_ESM.docx]

**Supplementary methods and materials**

**Immunohistochemistry**

The criteria for the evaluation of RNF38 positive staining were established as follows: dark brown staining was scored as 2+, brown staining as + and weak brown or absent staining as − after estimation of intensity. The mean area of positive staining (50 %) was used as a cut-off value to distinguish differential expression in proportion to measurement (score –, staining area ≤50 % of tumor section; score +, staining area >50 % of tumor section) using an integrated imaging system (MetaMorph Imaging System version 3.0; Universal Imaging Corp, Buckinghamshire UK). After a comprehensive assessment of both staining intensity and area presentation, which was performed by two independent researchers including a pathologist, patients with scores ≥2+ were considered to have high expression, and patients with scores <2+ were considered to have low expression.

**Liquid chromatography coupled with tandem mass spectrometry (LC-MS/MS)**

Briefly, the detergent, DTT and other low-molecular-weight components were removed using 200 μl UA buffer (8 M Urea, 150 mM Tris-HCl pH 8.0) by repeated ultrafiltration (Microcon units, 30 kD) facilitated by centrifugation. Then 100 μL 0.05 M iodoacetamide in UA buffer was added to block reduced cysteine residues and the samples were incubated for 20 min in darkness. The filter was washed with 100 μl UA buffer three times and then 100 μl 25 mM NH4HCO3 twice. Finally, the protein suspension was digested with 4 μg trypsin (Promega) in 40 μl 25 mM NH4HCO3 overnight at 37 °C, and the resulting peptides were collected as a filtrate. The peptide concentration was determined with OD280 by Nanodrop device. The peptide of each sample was desalted on C18 Cartridges (Empore™ SPE Cartridges, Sigma), then concentrated by vacuum centrifugation and reconstituted in 10 µl of 0.1% (v/v) Formic acid. MS experiments were performed on a Q Exactive HF mass spectrometer that was coupled to Easy nLC (Thermo Scientific). Peptide was first loaded onto a trap column (100μm*20mm, 5μm, C18) with 0.1% formic acid, then separated by an analytical column (75μm*120mm, 3μm, C18)) with a binary gradient of buffer A (2% acetonitrile and 0.1% Formic acid) and buffer B (80% acetonitrile and 0.1% Formic acid) at a flow rate of 300 nL/min over 60 min. The gradient was set as following: 2%–4% buffer B from 0 min to 2min, 4% to 30% buffer B from 2 min to 57 min, 30% to 45% buffer B from 57 min to 62 min,, 45% to 90% buffer B from 62 min to 64 min, 90% buffer B kept till to 75min. MS data was acquired using a data-dependent top20 method dynamically choosing the most abundant precursor ions from the survey scan (350–1800 m/z) for HCD fragmentation. A lock mass of 445.120025 Da was used as internal standard for mass calibration. The full MS scans were acquired at a resolution of 120,000 at m/z 200, and 15,000 at m/z 200 for MS/MS scan. The maximum injection time was set to for 50 ms for MS and 45 ms for MS/MS. Normalized collision energy was 28 and the isolation window was set to 1.2 Th. Dynamic exclusion duration was 60 s.

The MS data were analyzed using MaxQuant software version 1.6.0.16. MS data were searched against the UniProtKB Human database (162989 total entries, downloaded 04/14/2018). The trypsin was seleted as digestion enzyme. The maximal two missed cleavage sites and the mass tolerance of 4.5 ppm for precursor ions and 20 ppm for fragment ions were defined for database search. Carbamidomethylation of cysteines was defined as fixed modification, while acetylation of protein N-terminal and Lysine, oxidation of Methionine, ubiquitination of Lysine and phosphorylation of Serine, Threonine and Tyrosin were set as variable modifications for database searching. The database search results were filtered and exported with <1% false discovery rate (FDR) at peptide level, PTM site and protein level, respectively. PTM site localization and associated probability were determined by MaxQuant. Only high confidence PTM site annotations with P≥0.75 (localization probability) were accepted as Class I sites.
