# Supplementary material for: Overexpression of RNF38 facilitates TGF-β signaling by Ubiquitinating and degrading AHNAK in hepatocellular carcinoma
Source: J Exp Clin Cancer Res. 2019 Mar 5;38:113. doi: 10.1186/s13046-019-1113-3 (PMC6402116; doi:10.1186/s13046-019-1113-3)
Supplement: Supplementary file 2 — Table S1. Antibodies resource (DOCX 15 kb) [file 13046_2019_1113_MOESM2_ESM.docx]

Supplementary table 1. Antibodies resource

| Antibodies | Manufacturer (CAT) | Application |
| --- | --- | --- |
| RNF38 | Proteintech (25132-1-AP) | IF, WB, IHC |
| RNF38 | Santa Cruz (sc-515213) | IP, WB |
| GAPDH | Beyotime Biotechnology (AF006) | WB |
| Vimentin | Cell Signaling Technology (D21H3) | WB, IF |
| E-cadherin | Cell Signaling Technology (24E10) | WB, IF |
| Snail | NOVUS BIOLOGICALS (AF3639) | WB, IF |
| AHNAK | Abcam (ab68556) | IHC, IF, WB, IP |
| AHNAK | Abcam (ab168104) | WB |
| Ub | Santa Cruz (sc-8017) | WB |
| ERK | Cell Signaling Technology (4695S) | WB |
| p-ERK | Cell Signaling Technology (4370S) | WB |
| AKT | Cell Signaling Technology (9272S) | WB |
| p-AKT | Cell Signaling Technology (4685S) | WB |
| Smad2/3 | Cell Signaling Technology (8685S) | WB |
| p-Smad2/3 | Bioss (bs-8853R) | WB |
| TGFBR1 | Abcam (ab53647) | IHC, WB |
| Met | Abcam (ab51067) | WB |
| 14-3-3σ | Abcam (ab14123) | WB |
| TJP2 | Abcam (ab191133) | WB |
| CAV-1 | Abcam (ab85491) | WB |
| CD82 | Abcam (ab66400) | WB |

**Note:** IF, immunofluorescence; WB, Western Blotting; IHC, Immunohistochemistry; IP, immunoprecipitation
