# Supplementary figures and images for: Overexpression of RNF38 facilitates TGF-β signaling by Ubiquitinating and degrading AHNAK in hepatocellular carcinoma
Source: J Exp Clin Cancer Res. 2019 Mar 5;38:113. doi: 10.1186/s13046-019-1113-3 (PMC6402116; doi:10.1186/s13046-019-1113-3)

**A**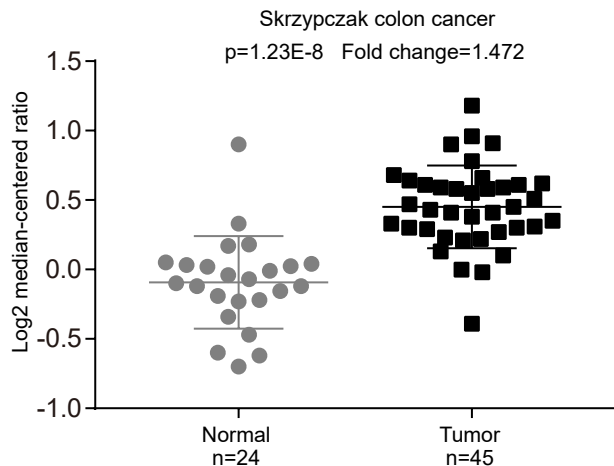**B**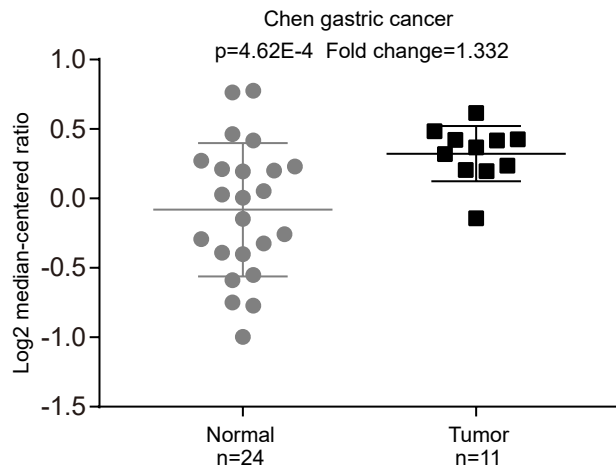**C**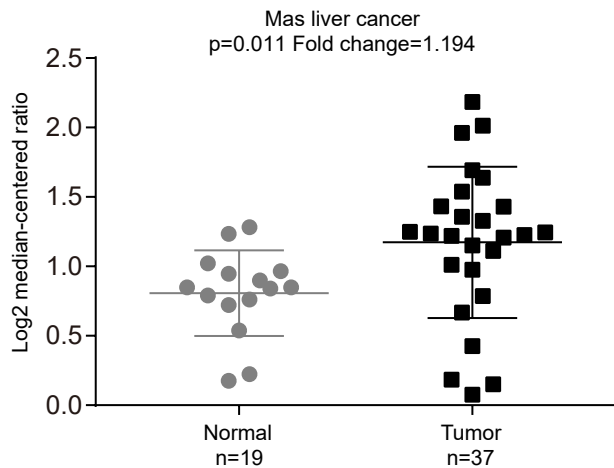**D**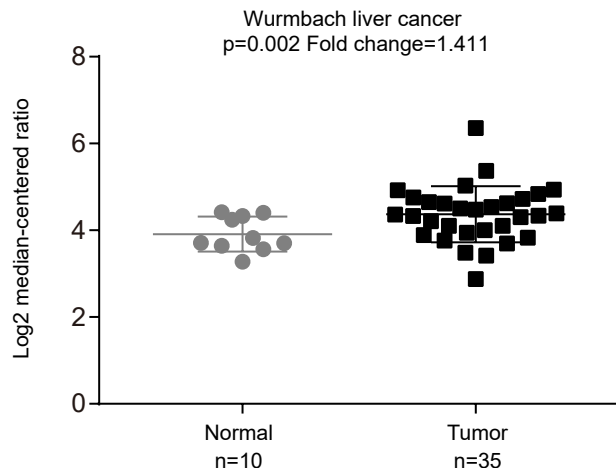

Supplement: Supplementary file 3 — Figure S1. Analysis RNF38 mRNA level in gastrointestinal tumor vs. normal tissues. A-D. RNF38 mRNA level was up-regulated in colon cancer, gastric cancer, and liver cancer, compared to normal tissues (All data retrieved from www.oncomine.org). (PDF 114 kb) [file 13046_2019_1113_MOESM3_ESM.pdf]

DAPI

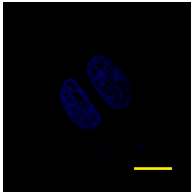

RNF38

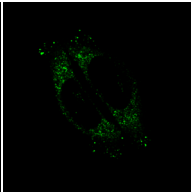

AHNAK

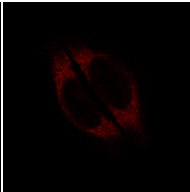

Merge

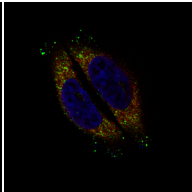

Supplement: Supplementary file 4 — Figure S2. Confocal microscopy images of RNF38 and AHNAK. Shown are HCCLM3-NC cells (blue, DAPI; green, RNF38; red, AHNAK). Scale: 2000 × . (PDF 113 kb) [file 13046_2019_1113_MOESM4_ESM.pdf]

# HCCLM3

WT

NC

siRNA1

siRNA2

siRNA3

AHNAK

GAPDH

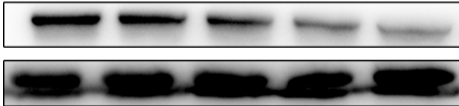

Supplement: Supplementary file 5 — Figure S3. The AHNAK expression in HCCLM3 and HCCLM3-siAHNAK cells. Western blot showed that the expression of AHNAK after transfecting the short interfering RNA to HCCLM3. (PDF 152 kb) [file 13046_2019_1113_MOESM5_ESM.pdf]

**A**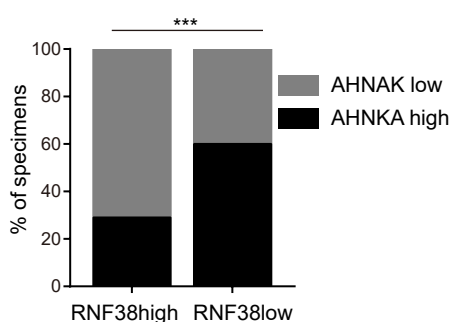**B**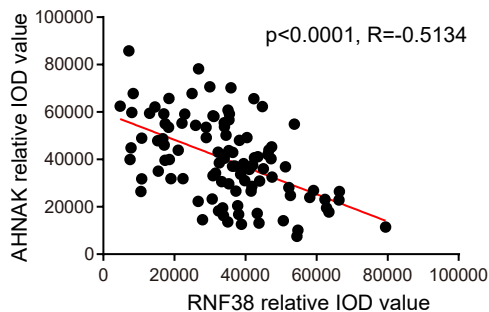**C**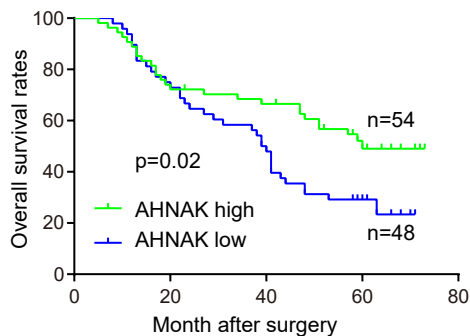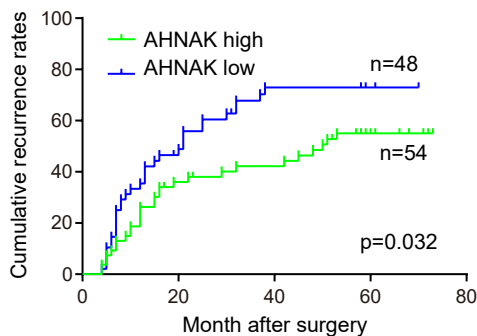**D**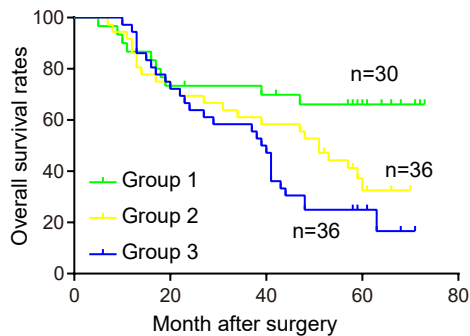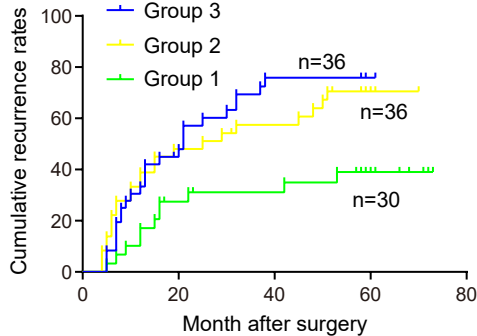

Supplement: Supplementary file 6 — Figure S4. Clinical relevance of RNF38 and/or AHNAK expression. A. The correlation between RNF38 and AHNAK were assessed by the Pearson correlation. B. RNF38 and AHNAK IOD relative value of 102 patients had negative correlation. C. HCC patients with low AHNAK had the lowest survival rate and the highest recurrence rate. D. HCC patients with high RNF38 and low AHNAK had the most unfavorable prognosis including the OS and recurrence. Group 1, patients of RNF38 low and AHNAK high (n = 30). Group 2, patients of both RNF38 and AHNAK low or high (n = 36), Group 3, patients of RNF38 high and AHNAK low (n = 36). (PDF 132 kb) [file 13046_2019_1113_MOESM6_ESM.pdf]
