# Supplementary material for: Overexpression of RNF38 facilitates TGF-β signaling by Ubiquitinating and degrading AHNAK in hepatocellular carcinoma
Source: J Exp Clin Cancer Res. 2019 Mar 5;38:113. doi: 10.1186/s13046-019-1113-3 (PMC6402116; doi:10.1186/s13046-019-1113-3)
Supplement: Supplementary file 7 — Table S2. SILAC of differential proteins in HepG2-RNF38 and HepG2-vector (Up-regulated) (DOCX 43 kb) [file 13046_2019_1113_MOESM7_ESM.docx]

Supplementary table 2. SILAC of differential proteins in HepG2-RNF38 and HepG2-vector (Up-regulated)

| Accession | Description | Score | Coverage | Fold change (≥1.5 times) |
| --- | --- | --- | --- | --- |
| Q16270 | Insulin-like growth factor-binding protein 7 OS=Homo sapiens GN=IGFBP7 PE=1 SV=1 - [IBP7_HUMAN] | 31.9 | 7.09 | 10.633 |
| Q6NUP7 | Serine/threonine-protein phosphatase 4 regulatory subunit 4 OS=Homo sapiens GN=PPP4R4 PE=1 SV=1 - [PP4R4_HUMAN] | 0 | 0.69 | 6.894 |
| Q7Z5R6 | Amyloid beta A4 precursor protein-binding family B member 1-interacting protein OS=Homo sapiens GN=APBB1IP PE=1 SV=1 - [AB1IP_HUMAN] | 77.72 | 4.2 | 5.803 |
| P02655 | Apolipoprotein C-II OS=Homo sapiens GN=APOC2 PE=1 SV=1 - [APOC2_HUMAN] | 44.14 | 8.91 | 5.242 |
| Q8TF61 | F-box only protein 41 OS=Homo sapiens GN=FBXO41 PE=2 SV=5 - [FBX41_HUMAN] | 28.18 | 1.14 | 4.503 |
| P11169 | Solute carrier family 2, facilitated glucose transporter member 3 OS=Homo sapiens GN=SLC2A3 PE=1 SV=1 - [GTR3_HUMAN] | 619.27 | 16.53 | 4.388 |
| Q96D42 | Hepatitis A virus cellular receptor 1 OS=Homo sapiens GN=HAVCR1 PE=1 SV=2 - [HAVR1_HUMAN] | 32.77 | 2.23 | 4.285 |
| Q969P6 | DNA topoisomerase I, mitochondrial OS=Homo sapiens GN=TOP1MT PE=1 SV=1 - [TOP1M_HUMAN] | 87.27 | 7.65 | 4.277 |
| Q9H0F5 | RING finger protein 38 OS=Homo sapiens GN=RNF38 PE=1 SV=2 - [RNF38_HUMAN] | 220.52 | 19.35 | 4.186 |
| P03915 | NADH-ubiquinone oxidoreductase chain 5 OS=Homo sapiens GN=MT-ND5 PE=1 SV=2 - [NU5M_HUMAN] | 29.29 | 2.82 | 4.097 |
| P40261 | Nicotinamide N-methyltransferase OS=Homo sapiens GN=NNMT PE=1 SV=1 - [NNMT_HUMAN] | 264.63 | 20.45 | 4.018 |
| Q05639 | Elongation factor 1-alpha 2 OS=Homo sapiens GN=EEF1A2 PE=1 SV=1 - [EF1A2_HUMAN] | 5571.74 | 37.8 | 3.977 |
| P80404 | 4-aminobutyrate aminotransferase, mitochondrial OS=Homo sapiens GN=ABAT PE=1 SV=3 - [GABT_HUMAN] | 26.02 | 1.6 | 3.936 |
| P35542 | Serum amyloid A-4 protein OS=Homo sapiens GN=SAA4 PE=1 SV=2 - [SAA4_HUMAN] | 26.12 | 6.15 | 3.736 |
| P37840 | Alpha-synuclein OS=Homo sapiens GN=SNCA PE=1 SV=1 - [SYUA_HUMAN] | 220.24 | 47.14 | 3.671 |
| Q15125 | 3-beta-hydroxysteroid-Delta(8),Delta(7)-isomerase OS=Homo sapiens GN=EBP PE=1 SV=3 - [EBP_HUMAN] | 154.2 | 12.17 | 3.607 |
| P06307 | Cholecystokinin OS=Homo sapiens GN=CCK PE=1 SV=1 - [CCKN_HUMAN] | 77.7 | 7.83 | 3.47 |
| P08697 | Alpha-2-antiplasmin OS=Homo sapiens GN=SERPINF2 PE=1 SV=3 - [A2AP_HUMAN] | 29.33 | 1.83 | 3.465 |
| Q9UJU2 | Lymphoid enhancer-binding factor 1 OS=Homo sapiens GN=LEF1 PE=1 SV=1 - [LEF1_HUMAN] | 39.74 | 2.76 | 3.396 |
| O95996 | Adenomatous polyposis coli protein 2 OS=Homo sapiens GN=APC2 PE=1 SV=1 - [APCL_HUMAN] | 0 | 0.78 | 3.216 |
| P08833 | Insulin-like growth factor-binding protein 1 OS=Homo sapiens GN=IGFBP1 PE=1 SV=1 - [IBP1_HUMAN] | 316.66 | 32.82 | 3.173 |
| Q96PZ7 | CUB and sushi domain-containing protein 1 OS=Homo sapiens GN=CSMD1 PE=1 SV=2 - [CSMD1_HUMAN] | 0 | 0.17 | 3.149 |
| Q2QL34 | Mpv17-like protein OS=Homo sapiens GN=MPV17L PE=1 SV=1 - [MP17L_HUMAN] | 34.66 | 5.1 | 3.007 |
| Q14244 | Ensconsin OS=Homo sapiens GN=MAP7 PE=1 SV=1 - [MAP7_HUMAN] | 22.76 | 4.94 | 2.93 |
| Q8IY57 | YY1-associated factor 2 OS=Homo sapiens GN=YAF2 PE=1 SV=3 - [YAF2_HUMAN] | 153.51 | 20 | 2.929 |
| Q5T2W1 | Na(+)/H(+) exchange regulatory cofactor NHE-RF3 OS=Homo sapiens GN=PDZK1 PE=1 SV=2 - [NHRF3_HUMAN] | 396.99 | 42.77 | 2.894 |
| O95025 | Semaphorin-3D OS=Homo sapiens GN=SEMA3D PE=2 SV=2 - [SEM3D_HUMAN] | 28.33 | 1.42 | 2.822 |
| Q9ULV0 | Unconventional myosin-Vb OS=Homo sapiens GN=MYO5B PE=1 SV=3 - [MYO5B_HUMAN] | 39.02 | 2.38 | 2.808 |
| Q9H361 | Polyadenylate-binding protein 3 OS=Homo sapiens GN=PABPC3 PE=1 SV=2 - [PABP3_HUMAN] | 1174.15 | 26.78 | 2.72 |
| Q9NQB0 | Transcription factor 7-like 2 OS=Homo sapiens GN=TCF7L2 PE=1 SV=2 - [TF7L2_HUMAN] | 31.89 | 1.45 | 2.697 |
| P27487 | Dipeptidyl peptidase 4 OS=Homo sapiens GN=DPP4 PE=1 SV=2 - [DPP4_HUMAN] | 61.85 | 6.01 | 2.684 |
| Q14116 | Interleukin-18 OS=Homo sapiens GN=IL18 PE=1 SV=1 - [IL18_HUMAN] | 196.64 | 40.41 | 2.655 |
| P11168 | Solute carrier family 2, facilitated glucose transporter member 2 OS=Homo sapiens GN=SLC2A2 PE=1 SV=1 - [GTR2_HUMAN] | 116.72 | 11.07 | 2.616 |
| Q96JB3 | Hypermethylated in cancer 2 protein OS=Homo sapiens GN=HIC2 PE=1 SV=2 - [HIC2_HUMAN] | 44.52 | 2.28 | 2.614 |
| P04083 | Annexin A1 OS=Homo sapiens GN=ANXA1 PE=1 SV=2 - [ANXA1_HUMAN] | 321.07 | 38.1 | 2.61 |
| Q9H477 | Ribokinase OS=Homo sapiens GN=RBKS PE=1 SV=1 - [RBSK_HUMAN] | 61.19 | 4.97 | 2.607 |
| O76038 | Secretagogin OS=Homo sapiens GN=SCGN PE=2 SV=2 - [SEGN_HUMAN] | 30.38 | 7.61 | 2.607 |
| Q9NYS0 | NF-kappa-B inhibitor-interacting Ras-like protein 1 OS=Homo sapiens GN=NKIRAS1 PE=1 SV=1 - [KBRS1_HUMAN] | 26.47 | 3.65 | 2.587 |
| Q9ULT6 | E3 ubiquitin-protein ligase ZNRF3 OS=Homo sapiens GN=ZNRF3 PE=1 SV=3 - [ZNRF3_HUMAN] | 44.26 | 1.28 | 2.547 |
| P12107 | Collagen alpha-1(XI) chain OS=Homo sapiens GN=COL11A1 PE=1 SV=4 - [COBA1_HUMAN] | 25.24 | 1.05 | 2.54 |
| A6NFI3 | Zinc finger protein 316 OS=Homo sapiens GN=ZNF316 PE=1 SV=1 - [ZN316_HUMAN] | 64.23 | 3.19 | 2.532 |
| Q8N1F1 | Putative uncharacterized protein LRRC75A-AS1, mitochondrial OS=Homo sapiens GN=LRRC75A-AS1 PE=5 SV=1 - [C1AS1_HUMAN] | 0 | 5.38 | 2.515 |
| Q9P2K5 | Myelin expression factor 2 OS=Homo sapiens GN=MYEF2 PE=1 SV=3 - [MYEF2_HUMAN] | 71.36 | 11.33 | 2.504 |
| Q6P4I2 | WD repeat-containing protein 73 OS=Homo sapiens GN=WDR73 PE=1 SV=1 - [WDR73_HUMAN] | 23.39 | 4.5 | 2.494 |
| Q6ZR08 | Dynein heavy chain 12, axonemal OS=Homo sapiens GN=DNAH12 PE=2 SV=2 - [DYH12_HUMAN] | 30.74 | 0.36 | 2.488 |
| O14975 | Very long-chain acyl-CoA synthetase OS=Homo sapiens GN=SLC27A2 PE=1 SV=2 - [S27A2_HUMAN] | 136.77 | 13.23 | 2.458 |
| P12277 | Creatine kinase B-type OS=Homo sapiens GN=CKB PE=1 SV=1 - [KCRB_HUMAN] | 2109.24 | 57.74 | 2.431 |
| P34741 | Syndecan-2 OS=Homo sapiens GN=SDC2 PE=1 SV=2 - [SDC2_HUMAN] | 44.13 | 11.94 | 2.422 |
| Q9H7M9 | V-type immunoglobulin domain-containing suppressor of T-cell activation OS=Homo sapiens GN=VSIR PE=1 SV=3 - [VISTA_HUMAN] | 41.75 | 9.65 | 2.405 |
| P42677 | 40S ribosomal protein S27 OS=Homo sapiens GN=RPS27 PE=1 SV=3 - [RS27_HUMAN] | 471.13 | 39.29 | 2.399 |
| Q8N159 | N-acetylglutamate synthase, mitochondrial OS=Homo sapiens GN=NAGS PE=1 SV=1 - [NAGS_HUMAN] | 0 | 1.31 | 2.366 |
| Q96ES7 | SAGA-associated factor 29 OS=Homo sapiens GN=SGF29 PE=1 SV=1 - [SGF29_HUMAN] | 28.11 | 5.46 | 2.346 |
| Q15154 | Pericentriolar material 1 protein OS=Homo sapiens GN=PCM1 PE=1 SV=4 - [PCM1_HUMAN] | 2663.84 | 35.33 | 2.321 |
| P27701 | CD82 antigen OS=Homo sapiens GN=CD82 PE=1 SV=1 - [CD82_HUMAN] | 419.32 | 51.65 | 2.29 |
| Q9C075 | Keratin, type I cytoskeletal 23 OS=Homo sapiens GN=KRT23 PE=1 SV=2 - [K1C23_HUMAN] | 125.67 | 21.09 | 2.281 |
| Q6ZVX9 | Membrane progestin receptor epsilon OS=Homo sapiens GN=PAQR9 PE=1 SV=1 - [PAQR9_HUMAN] | 49.26 | 5.84 | 2.278 |
| Q92484 | Acid sphingomyelinase-like phosphodiesterase 3a OS=Homo sapiens GN=SMPDL3A PE=1 SV=2 - [ASM3A_HUMAN] | 44.43 | 6.84 | 2.262 |
| P04440 | HLA class II histocompatibility antigen, DP beta 1 chain OS=Homo sapiens GN=HLA-DPB1 PE=1 SV=1 - [DPB1_HUMAN] | 25.4 | 2.71 | 2.258 |
| O43657 | Tetraspanin-6 OS=Homo sapiens GN=TSPAN6 PE=1 SV=1 - [TSN6_HUMAN] | 131.55 | 15.51 | 2.253 |
| P84022 | Mothers against decapentaplegic homolog 3 OS=Homo sapiens GN=SMAD3 PE=1 SV=1 - [SMAD3_HUMAN] | 102.25 | 16.94 | 2.249 |
| Q0ZGT2 | Nexilin OS=Homo sapiens GN=NEXN PE=1 SV=1 - [NEXN_HUMAN] | 84.39 | 4 | 2.248 |
| O14713 | Integrin beta-1-binding protein 1 OS=Homo sapiens GN=ITGB1BP1 PE=1 SV=1 - [ITBP1_HUMAN] | 24.47 | 5.5 | 2.239 |
| Q14766 | Latent-transforming growth factor beta-binding protein 1 OS=Homo sapiens GN=LTBP1 PE=1 SV=4 - [LTBP1_HUMAN] | 33.68 | 0.58 | 2.236 |
| Q8TF42 | Ubiquitin-associated and SH3 domain-containing protein B OS=Homo sapiens GN=UBASH3B PE=1 SV=2 - [UBS3B_HUMAN] | 120.42 | 10.94 | 2.232 |
| Q92686 | Neurogranin OS=Homo sapiens GN=NRGN PE=1 SV=1 - [NEUG_HUMAN] | 0 | 19.23 | 2.201 |
| O15119 | T-box transcription factor TBX3 OS=Homo sapiens GN=TBX3 PE=1 SV=4 - [TBX3_HUMAN] | 23.02 | 2.42 | 2.201 |
| Q15654 | Thyroid receptor-interacting protein 6 OS=Homo sapiens GN=TRIP6 PE=1 SV=3 - [TRIP6_HUMAN] | 28.43 | 6.93 | 2.193 |
| Q8IUH5 | Palmitoyltransferase ZDHHC17 OS=Homo sapiens GN=ZDHHC17 PE=1 SV=2 - [ZDH17_HUMAN] | 104.01 | 3.01 | 2.183 |
| P23443 | Ribosomal protein S6 kinase beta-1 OS=Homo sapiens GN=RPS6KB1 PE=1 SV=2 - [KS6B1_HUMAN] | 27.86 | 2.29 | 2.179 |
| Q96GJ1 | tRNA (uracil(54)-C(5))-methyltransferase homolog OS=Homo sapiens GN=TRMT2B PE=1 SV=1 - [TRM2_HUMAN] | 122.19 | 2.98 | 2.175 |
| Q9P021 | Cysteine-rich PDZ-binding protein OS=Homo sapiens GN=CRIPT PE=1 SV=1 - [CRIPT_HUMAN] | 30.38 | 18.81 | 2.162 |
| Q9UJ68 | Mitochondrial peptide methionine sulfoxide reductase OS=Homo sapiens GN=MSRA PE=1 SV=1 - [MSRA_HUMAN] | 40.51 | 10.21 | 2.14 |
| P80723 | Brain acid soluble protein 1 OS=Homo sapiens GN=BASP1 PE=1 SV=2 - [BASP1_HUMAN] | 47.11 | 44.05 | 2.139 |
| P63208 | S-phase kinase-associated protein 1 OS=Homo sapiens GN=SKP1 PE=1 SV=2 - [SKP1_HUMAN] | 547.49 | 51.53 | 2.136 |
| Q14032 | Bile acid-CoA:amino acid N-acyltransferase OS=Homo sapiens GN=BAAT PE=1 SV=1 - [BAAT_HUMAN] | 24.46 | 2.15 | 2.134 |
| P28065 | Proteasome subunit beta type-9 OS=Homo sapiens GN=PSMB9 PE=1 SV=2 - [PSB9_HUMAN] | 41.25 | 4.57 | 2.133 |
| Q96SW2 | Protein cereblon OS=Homo sapiens GN=CRBN PE=1 SV=1 - [CRBN_HUMAN] | 42.03 | 2.26 | 2.132 |
| Q15796 | Mothers against decapentaplegic homolog 2 OS=Homo sapiens GN=SMAD2 PE=1 SV=1 - [SMAD2_HUMAN] | 76.73 | 16.92 | 2.112 |
| Q9NRZ9 | Lymphoid-specific helicase OS=Homo sapiens GN=HELLS PE=1 SV=1 - [HELLS_HUMAN] | 329.7 | 23.75 | 2.101 |
| O00622 | Protein CYR61 OS=Homo sapiens GN=CYR61 PE=1 SV=1 - [CYR61_HUMAN] | 26.12 | 4.2 | 2.095 |
| P28347 | Transcriptional enhancer factor TEF-1 OS=Homo sapiens GN=TEAD1 PE=1 SV=2 - [TEAD1_HUMAN] | 28.11 | 3.52 | 2.085 |
| Q09666 | Neuroblast differentiation-associated protein AHNAK OS=Homo sapiens GN=AHNAK PE=1 SV=2 - [AHNK_HUMAN] | 6992.26 | 64.82 | 2.084 |
| Q96IQ9 | Zinc finger protein 414 OS=Homo sapiens GN=ZNF414 PE=1 SV=2 - [ZN414_HUMAN] | 37.5 | 7.05 | 2.077 |
| Q15562 | Transcriptional enhancer factor TEF-4 OS=Homo sapiens GN=TEAD2 PE=1 SV=2 - [TEAD2_HUMAN] | 28.11 | 3.36 | 2.075 |
| Q9UH99 | SUN domain-containing protein 2 OS=Homo sapiens GN=SUN2 PE=1 SV=3 - [SUN2_HUMAN] | 140.15 | 8.51 | 2.068 |
| Q86YZ3 | Hornerin OS=Homo sapiens GN=HRNR PE=1 SV=2 - [HORN_HUMAN] | 28.5 | 4.25 | 2.056 |
| Q9BW83 | Intraflagellar transport protein 27 homolog OS=Homo sapiens GN=IFT27 PE=1 SV=1 - [IFT27_HUMAN] | 72.47 | 31.18 | 2.055 |
| Q9UBF6 | RING-box protein 2 OS=Homo sapiens GN=RNF7 PE=1 SV=1 - [RBX2_HUMAN] | 62.54 | 19.47 | 2.054 |
| Q9Y2H6 | Fibronectin type-III domain-containing protein 3A OS=Homo sapiens GN=FNDC3A PE=1 SV=4 - [FND3A_HUMAN] | 396.55 | 20.37 | 2.051 |
| Q86UT8 | Coiled-coil domain-containing protein 84 OS=Homo sapiens GN=CCDC84 PE=1 SV=1 - [CCD84_HUMAN] | 0 | 1.81 | 2.042 |
| Q9NQ29 | Putative RNA-binding protein Luc7-like 1 OS=Homo sapiens GN=LUC7L PE=1 SV=1 - [LUC7L_HUMAN] | 563.81 | 19.68 | 2.033 |
| P09341 | Growth-regulated alpha protein OS=Homo sapiens GN=CXCL1 PE=1 SV=1 - [GROA_HUMAN] | 37.77 | 7.48 | 2.028 |
| Q9Y3E1 | Hepatoma-derived growth factor-related protein 3 OS=Homo sapiens GN=HDGFL3 PE=1 SV=1 - [HDGR3_HUMAN] | 187.34 | 18.23 | 2.02 |
| Q9C073 | Protein FAM117A OS=Homo sapiens GN=FAM117A PE=1 SV=1 - [F117A_HUMAN] | 33.57 | 1.55 | 2.012 |
| Q96A46 | Mitoferrin-2 OS=Homo sapiens GN=SLC25A28 PE=2 SV=1 - [MFRN2_HUMAN] | 49.73 | 4.12 | 2.011 |
| Q9NVW2 | E3 ubiquitin-protein ligase RLIM OS=Homo sapiens GN=RLIM PE=1 SV=3 - [RNF12_HUMAN] | 87.83 | 5.45 | 2.007 |
| Q9H939 | Proline-serine-threonine phosphatase-interacting protein 2 OS=Homo sapiens GN=PSTPIP2 PE=1 SV=4 - [PPIP2_HUMAN] | 34.76 | 4.19 | 2.007 |
| P61626 | Lysozyme C OS=Homo sapiens GN=LYZ PE=1 SV=1 - [LYSC_HUMAN] | 22.39 | 0.17 | 2 |
| Q8WXX5 | DnaJ homolog subfamily C member 9 OS=Homo sapiens GN=DNAJC9 PE=1 SV=1 - [DNJC9_HUMAN] | 512.49 | 56.54 | 1.993 |
| Q9HAU4 | E3 ubiquitin-protein ligase SMURF2 OS=Homo sapiens GN=SMURF2 PE=1 SV=1 - [SMUF2_HUMAN] | 31.4 | 1.2 | 1.989 |
| Q13485 | Mothers against decapentaplegic homolog 4 OS=Homo sapiens GN=SMAD4 PE=1 SV=1 - [SMAD4_HUMAN] | 143.71 | 14.31 | 1.984 |
| Q70Z53 | Protein FRA10AC1 OS=Homo sapiens GN=FRA10AC1 PE=1 SV=3 - [F10C1_HUMAN] | 32.45 | 6.67 | 1.975 |
| Q13145 | BMP and activin membrane-bound inhibitor homolog OS=Homo sapiens GN=BAMBI PE=1 SV=1 - [BAMBI_HUMAN] | 32.07 | 3.46 | 1.966 |
| P62861 | 40S ribosomal protein S30 OS=Homo sapiens GN=FAU PE=1 SV=1 - [RS30_HUMAN] | 31.17 | 11.86 | 1.961 |
| Q9Y5J9 | Mitochondrial import inner membrane translocase subunit Tim8 B OS=Homo sapiens GN=TIMM8B PE=1 SV=1 - [TIM8B_HUMAN] | 260.51 | 51.81 | 1.938 |
| Q9NPB8 | Glycerophosphocholine phosphodiesterase GPCPD1 OS=Homo sapiens GN=GPCPD1 PE=1 SV=2 - [GPCP1_HUMAN] | 70.75 | 1.93 | 1.938 |
| Q6ZUT1 | Uncharacterized protein NKAPD1 OS=Homo sapiens GN=NKAPD1 PE=1 SV=2 - [NKAP1_HUMAN] | 28.41 | 4.79 | 1.931 |
| P48163 | NADP-dependent malic enzyme OS=Homo sapiens GN=ME1 PE=1 SV=1 - [MAOX_HUMAN] | 975.16 | 17.27 | 1.929 |
| Q8WUH2 | Transforming growth factor-beta receptor-associated protein 1 OS=Homo sapiens GN=TGFBRAP1 PE=1 SV=1 - [TGFA1_HUMAN] | 77.22 | 2.67 | 1.928 |
| Q6IPM2 | IQ domain-containing protein E OS=Homo sapiens GN=IQCE PE=1 SV=2 - [IQCE_HUMAN] | 0 | 2.01 | 1.927 |
| Q01459 | Di-N-acetylchitobiase OS=Homo sapiens GN=CTBS PE=1 SV=1 - [DIAC_HUMAN] | 49.09 | 6.49 | 1.927 |
| Q7Z6B0 | Coiled-coil domain-containing protein 91 OS=Homo sapiens GN=CCDC91 PE=1 SV=2 - [CCD91_HUMAN] | 36.15 | 3.85 | 1.922 |
| P98161 | Polycystin-1 OS=Homo sapiens GN=PKD1 PE=1 SV=3 - [PKD1_HUMAN] | 0 | 0.14 | 1.917 |
| Q52LW3 | Rho GTPase-activating protein 29 OS=Homo sapiens GN=ARHGAP29 PE=1 SV=2 - [RHG29_HUMAN] | 468.39 | 20.06 | 1.914 |
| Q99717 | Mothers against decapentaplegic homolog 5 OS=Homo sapiens GN=SMAD5 PE=1 SV=1 - [SMAD5_HUMAN] | 34.41 | 8.6 | 1.913 |
| P02788 | Lactotransferrin OS=Homo sapiens GN=LTF PE=1 SV=6 - [TRFL_HUMAN] | 35.77 | 1.13 | 1.912 |
| P62877 | E3 ubiquitin-protein ligase RBX1 OS=Homo sapiens GN=RBX1 PE=1 SV=1 - [RBX1_HUMAN] | 341.89 | 29.63 | 1.904 |
| Q66GS9 | Centrosomal protein of 135 kDa OS=Homo sapiens GN=CEP135 PE=1 SV=2 - [CP135_HUMAN] | 32.02 | 3.25 | 1.897 |
| Q9Y2I1 | Nischarin OS=Homo sapiens GN=NISCH PE=1 SV=3 - [NISCH_HUMAN] | 26.98 | 0.66 | 1.893 |
| Q9BRQ6 | MICOS complex subunit MIC25 OS=Homo sapiens GN=CHCHD6 PE=1 SV=1 - [MIC25_HUMAN] | 153.46 | 40.43 | 1.89 |
| P05937 | Calbindin OS=Homo sapiens GN=CALB1 PE=1 SV=2 - [CALB1_HUMAN] | 292.89 | 43.68 | 1.889 |
| Q96FC7 | Phytanoyl-CoA hydroxylase-interacting protein-like OS=Homo sapiens GN=PHYHIPL PE=1 SV=3 - [PHIPL_HUMAN] | 87.59 | 13.3 | 1.883 |
| Q9H7H0 | Methyltransferase-like protein 17, mitochondrial OS=Homo sapiens GN=METTL17 PE=1 SV=1 - [MET17_HUMAN] | 27.93 | 3.95 | 1.882 |
| Q9BX46 | RNA-binding protein 24 OS=Homo sapiens GN=RBM24 PE=1 SV=1 - [RBM24_HUMAN] | 0 | 6.36 | 1.873 |
| P62987 | Ubiquitin-60S ribosomal protein L40 OS=Homo sapiens GN=UBA52 PE=1 SV=2 - [RL40_HUMAN] | 3298.56 | 50.78 | 1.873 |
| P63096 | Guanine nucleotide-binding protein G(i) subunit alpha-1 OS=Homo sapiens GN=GNAI1 PE=1 SV=2 - [GNAI1_HUMAN] | 76.47 | 17.51 | 1.872 |
| Q969H0 | F-box/WD repeat-containing protein 7 OS=Homo sapiens GN=FBXW7 PE=1 SV=1 - [FBXW7_HUMAN] | 33.93 | 1.27 | 1.871 |
| P01009 | Alpha-1-antitrypsin OS=Homo sapiens GN=SERPINA1 PE=1 SV=3 - [A1AT_HUMAN] | 385.86 | 43.3 | 1.871 |
| Q9HAT2 | Sialate O-acetylesterase OS=Homo sapiens GN=SIAE PE=1 SV=1 - [SIAE_HUMAN] | 66.76 | 6.5 | 1.869 |
| Q9BRV3 | Sugar transporter SWEET1 OS=Homo sapiens GN=SLC50A1 PE=2 SV=1 - [SWET1_HUMAN] | 28.74 | 6.33 | 1.869 |
| Q15329 | Transcription factor E2F5 OS=Homo sapiens GN=E2F5 PE=1 SV=1 - [E2F5_HUMAN] | 31.87 | 4.34 | 1.869 |
| Q13129 | Zinc finger protein Rlf OS=Homo sapiens GN=RLF PE=1 SV=2 - [RLF_HUMAN] | 202.48 | 6.22 | 1.868 |
| Q96JB6 | Lysyl oxidase homolog 4 OS=Homo sapiens GN=LOXL4 PE=1 SV=1 - [LOXL4_HUMAN] | 46.86 | 1.32 | 1.867 |
| P54851 | Epithelial membrane protein 2 OS=Homo sapiens GN=EMP2 PE=1 SV=1 - [EMP2_HUMAN] | 26.38 | 3.59 | 1.867 |
| O15061 | Synemin OS=Homo sapiens GN=SYNM PE=1 SV=2 - [SYNEM_HUMAN] | 0 | 0.7 | 1.867 |
| O60502 | Protein O-GlcNAcase OS=Homo sapiens GN=MGEA5 PE=1 SV=2 - [OGA_HUMAN] | 389.25 | 15.5 | 1.866 |
| O43761 | Synaptogyrin-3 OS=Homo sapiens GN=SYNGR3 PE=1 SV=2 - [SNG3_HUMAN] | 64.41 | 11.79 | 1.865 |
| O95405 | Zinc finger FYVE domain-containing protein 9 OS=Homo sapiens GN=ZFYVE9 PE=1 SV=2 - [ZFYV9_HUMAN] | 35.52 | 1.05 | 1.863 |
| Q9H2G4 | Testis-specific Y-encoded-like protein 2 OS=Homo sapiens GN=TSPYL2 PE=1 SV=1 - [TSYL2_HUMAN] | 54.24 | 6.06 | 1.861 |
| P04908 | Histone H2A type 1-B/E OS=Homo sapiens GN=HIST1H2AB PE=1 SV=2 - [H2A1B_HUMAN] | 1378.74 | 28.46 | 1.852 |
| Q8IYT2 | Cap-specific mRNA (nucleoside-2'-O-)-methyltransferase 2 OS=Homo sapiens GN=CMTR2 PE=1 SV=2 - [CMTR2_HUMAN] | 0 | 1.3 | 1.851 |
| Q5M7Z0 | E3 ubiquitin-protein ligase RNFT1 OS=Homo sapiens GN=RNFT1 PE=1 SV=2 - [RNFT1_HUMAN] | 34.22 | 2.07 | 1.851 |
| Q9Y624 | Junctional adhesion molecule A OS=Homo sapiens GN=F11R PE=1 SV=1 - [JAM1_HUMAN] | 227.18 | 41.14 | 1.85 |
| Q6PFW1 | Inositol hexakisphosphate and diphosphoinositol-pentakisphosphate kinase 1 OS=Homo sapiens GN=PPIP5K1 PE=1 SV=1 - [VIP1_HUMAN] | 38.83 | 2.23 | 1.848 |
| P00533 | Epidermal growth factor receptor OS=Homo sapiens GN=EGFR PE=1 SV=2 - [EGFR_HUMAN] | 525.05 | 19.59 | 1.847 |
| Q9NUI1 | Peroxisomal 2,4-dienoyl-CoA reductase OS=Homo sapiens GN=DECR2 PE=1 SV=1 - [DECR2_HUMAN] | 45.67 | 3.77 | 1.843 |
| Q5SXM2 | snRNA-activating protein complex subunit 4 OS=Homo sapiens GN=SNAPC4 PE=1 SV=1 - [SNPC4_HUMAN] | 59.81 | 1.16 | 1.843 |
| O60503 | Adenylate cyclase type 9 OS=Homo sapiens GN=ADCY9 PE=1 SV=4 - [ADCY9_HUMAN] | 36.9 | 1.55 | 1.84 |
| Q96Q89 | Kinesin-like protein KIF20B OS=Homo sapiens GN=KIF20B PE=1 SV=3 - [KI20B_HUMAN] | 88.24 | 7.58 | 1.835 |
| Q6P582 | Mitotic-spindle organizing protein 2A OS=Homo sapiens GN=MZT2A PE=1 SV=2 - [MZT2A_HUMAN] | 58.76 | 32.91 | 1.834 |
| Q6IPR3 | tRNA wybutosine-synthesizing protein 3 homolog OS=Homo sapiens GN=TYW3 PE=2 SV=2 - [TYW3_HUMAN] | 63.26 | 10.04 | 1.832 |
| Q15750 | TGF-beta-activated kinase 1 and MAP3K7-binding protein 1 OS=Homo sapiens GN=TAB1 PE=1 SV=1 - [TAB1_HUMAN] | 136.01 | 18.25 | 1.832 |
| Q9Y4E8 | Ubiquitin carboxyl-terminal hydrolase 15 OS=Homo sapiens GN=USP15 PE=1 SV=3 - [UBP15_HUMAN] | 238.49 | 22.12 | 1.83 |
| P30408 | Transmembrane 4 L6 family member 1 OS=Homo sapiens GN=TM4SF1 PE=1 SV=1 - [T4S1_HUMAN] | 115.75 | 4.46 | 1.83 |
| Q9H3K2 | Growth hormone-inducible transmembrane protein OS=Homo sapiens GN=GHITM PE=1 SV=2 - [GHITM_HUMAN] | 169.09 | 9.57 | 1.829 |
| O14545 | TRAF-type zinc finger domain-containing protein 1 OS=Homo sapiens GN=TRAFD1 PE=1 SV=1 - [TRAD1_HUMAN] | 48.17 | 2.23 | 1.82 |
| P49902 | Cytosolic purine 5'-nucleotidase OS=Homo sapiens GN=NT5C2 PE=1 SV=1 - [5NTC_HUMAN] | 482.62 | 26.74 | 1.813 |
| Q9HBM6 | Transcription initiation factor TFIID subunit 9B OS=Homo sapiens GN=TAF9B PE=1 SV=1 - [TAF9B_HUMAN] | 144.69 | 28.29 | 1.812 |
| P54278 | Mismatch repair endonuclease PMS2 OS=Homo sapiens GN=PMS2 PE=1 SV=2 - [PMS2_HUMAN] | 162.43 | 10.21 | 1.81 |
| Q02446 | Transcription factor Sp4 OS=Homo sapiens GN=SP4 PE=1 SV=2 - [SP4_HUMAN] | 0 | 1.91 | 1.809 |
| Q9H8M5 | Metal transporter CNNM2 OS=Homo sapiens GN=CNNM2 PE=1 SV=2 - [CNNM2_HUMAN] | 77.42 | 5.83 | 1.795 |
| Q9H7X3 | Zinc finger protein 696 OS=Homo sapiens GN=ZNF696 PE=1 SV=2 - [ZN696_HUMAN] | 52.97 | 6.68 | 1.794 |
| Q8N9R8 | Protein SCAI OS=Homo sapiens GN=SCAI PE=1 SV=2 - [SCAI_HUMAN] | 24.42 | 1.32 | 1.789 |
| P62834 | Ras-related protein Rap-1A OS=Homo sapiens GN=RAP1A PE=1 SV=1 - [RAP1A_HUMAN] | 249.27 | 28.26 | 1.787 |
| Q9H8W3 | Protein FAM204A OS=Homo sapiens GN=FAM204A PE=2 SV=1 - [F204A_HUMAN] | 33.87 | 6.44 | 1.78 |
| Q68CZ2 | Tensin-3 OS=Homo sapiens GN=TNS3 PE=1 SV=2 - [TENS3_HUMAN] | 154.19 | 5.74 | 1.769 |
| Q15043 | Zinc transporter ZIP14 OS=Homo sapiens GN=SLC39A14 PE=1 SV=3 - [S39AE_HUMAN] | 433.77 | 12.6 | 1.769 |
| P17568 | NADH dehydrogenase [ubiquinone] 1 beta subcomplex subunit 7 OS=Homo sapiens GN=NDUFB7 PE=1 SV=4 - [NDUB7_HUMAN] | 57.96 | 14.6 | 1.768 |
| P07199 | Major centromere autoantigen B OS=Homo sapiens GN=CENPB PE=1 SV=2 - [CENPB_HUMAN] | 100.44 | 6.01 | 1.764 |
| Q9BVA0 | Katanin p80 WD40 repeat-containing subunit B1 OS=Homo sapiens GN=KATNB1 PE=1 SV=1 - [KTNB1_HUMAN] | 24.75 | 2.14 | 1.759 |
| Q03169 | Tumor necrosis factor alpha-induced protein 2 OS=Homo sapiens GN=TNFAIP2 PE=2 SV=2 - [TNAP2_HUMAN] | 92.88 | 7.34 | 1.755 |
| Q96AJ9 | Vesicle transport through interaction with t-SNAREs homolog 1A OS=Homo sapiens GN=VTI1A PE=1 SV=2 - [VTI1A_HUMAN] | 63.92 | 15.21 | 1.751 |
| O43677 | NADH dehydrogenase [ubiquinone] 1 subunit C1, mitochondrial OS=Homo sapiens GN=NDUFC1 PE=1 SV=1 - [NDUC1_HUMAN] | 0 | 27.63 | 1.75 |
| Q68DH5 | LMBR1 domain-containing protein 2 OS=Homo sapiens GN=LMBRD2 PE=1 SV=1 - [LMBD2_HUMAN] | 35.22 | 3.17 | 1.746 |
| Q14781 | Chromobox protein homolog 2 OS=Homo sapiens GN=CBX2 PE=1 SV=2 - [CBX2_HUMAN] | 51.99 | 5.83 | 1.74 |
| O75528 | Transcriptional adapter 3 OS=Homo sapiens GN=TADA3 PE=1 SV=1 - [TADA3_HUMAN] | 93.88 | 9.95 | 1.74 |
| O43805 | Sjoegren syndrome nuclear autoantigen 1 OS=Homo sapiens GN=SSNA1 PE=1 SV=2 - [SSNA1_HUMAN] | 104.74 | 30.25 | 1.738 |
| P08069 | Insulin-like growth factor 1 receptor OS=Homo sapiens GN=IGF1R PE=1 SV=1 - [IGF1R_HUMAN] | 74.16 | 4.24 | 1.737 |
| Q9UBU8 | Mortality factor 4-like protein 1 OS=Homo sapiens GN=MORF4L1 PE=1 SV=2 - [MO4L1_HUMAN] | 328.44 | 27.35 | 1.731 |
| Q9H7P9 | Pleckstrin homology domain-containing family G member 2 OS=Homo sapiens GN=PLEKHG2 PE=1 SV=3 - [PKHG2_HUMAN] | 0 | 1.37 | 1.729 |
| P03891 | NADH-ubiquinone oxidoreductase chain 2 OS=Homo sapiens GN=MT-ND2 PE=1 SV=2 - [NU2M_HUMAN] | 45.85 | 4.61 | 1.728 |
| O76041 | Nebulette OS=Homo sapiens GN=NEBL PE=1 SV=1 - [NEBL_HUMAN] | 50.99 | 1.78 | 1.728 |
| Q4VCS5 | Angiomotin OS=Homo sapiens GN=AMOT PE=1 SV=1 - [AMOT_HUMAN] | 41.9 | 3.14 | 1.725 |
| Q9NX76 | CKLF-like MARVEL transmembrane domain-containing protein 6 OS=Homo sapiens GN=CMTM6 PE=1 SV=1 - [CKLF6_HUMAN] | 48.88 | 9.29 | 1.722 |
| Q8N9B5 | Junction-mediating and -regulatory protein OS=Homo sapiens GN=JMY PE=1 SV=2 - [JMY_HUMAN] | 74.39 | 1.42 | 1.718 |
| P62979 | Ubiquitin-40S ribosomal protein S27a OS=Homo sapiens GN=RPS27A PE=1 SV=2 - [RS27A_HUMAN] | 3411.4 | 54.49 | 1.713 |
| Q8IY22 | C-Maf-inducing protein OS=Homo sapiens GN=CMIP PE=1 SV=3 - [CMIP_HUMAN] | 71 | 5.82 | 1.711 |
| O43181 | NADH dehydrogenase [ubiquinone] iron-sulfur protein 4, mitochondrial OS=Homo sapiens GN=NDUFS4 PE=1 SV=1 - [NDUS4_HUMAN] | 217.11 | 34.86 | 1.708 |
| P10768 | S-formylglutathione hydrolase OS=Homo sapiens GN=ESD PE=1 SV=2 - [ESTD_HUMAN] | 323.9 | 42.91 | 1.707 |
| P53611 | Geranylgeranyl transferase type-2 subunit beta OS=Homo sapiens GN=RABGGTB PE=1 SV=2 - [PGTB2_HUMAN] | 244.25 | 26.59 | 1.703 |
| Q13526 | Peptidyl-prolyl cis-trans isomerase NIMA-interacting 1 OS=Homo sapiens GN=PIN1 PE=1 SV=1 - [PIN1_HUMAN] | 103.08 | 39.88 | 1.699 |
| Q96EH3 | Mitochondrial assembly of ribosomal large subunit protein 1 OS=Homo sapiens GN=MALSU1 PE=1 SV=1 - [MASU1_HUMAN] | 39.23 | 8.97 | 1.698 |
| Q00403 | Transcription initiation factor IIB OS=Homo sapiens GN=GTF2B PE=1 SV=1 - [TF2B_HUMAN] | 303.7 | 31.65 | 1.698 |
| Q9NP77 | RNA polymerase II subunit A C-terminal domain phosphatase SSU72 OS=Homo sapiens GN=SSU72 PE=1 SV=1 - [SSU72_HUMAN] | 52.48 | 15.46 | 1.697 |
| Q8WTT2 | Nucleolar complex protein 3 homolog OS=Homo sapiens GN=NOC3L PE=1 SV=1 - [NOC3L_HUMAN] | 446.92 | 24.75 | 1.693 |
| Q96AY2 | Crossover junction endonuclease EME1 OS=Homo sapiens GN=EME1 PE=1 SV=2 - [EME1_HUMAN] | 25.54 | 1.58 | 1.692 |
| Q92747 | Actin-related protein 2/3 complex subunit 1A OS=Homo sapiens GN=ARPC1A PE=2 SV=2 - [ARC1A_HUMAN] | 87.26 | 17.3 | 1.692 |
| Q9ULX9 | Transcription factor MafF OS=Homo sapiens GN=MAFF PE=1 SV=2 - [MAFF_HUMAN] | 26.69 | 14.02 | 1.69 |
| Q9P0K8 | Forkhead box protein J2 OS=Homo sapiens GN=FOXJ2 PE=1 SV=1 - [FOXJ2_HUMAN] | 29.99 | 2.96 | 1.687 |
| P07205 | Phosphoglycerate kinase 2 OS=Homo sapiens GN=PGK2 PE=1 SV=3 - [PGK2_HUMAN] | 2197.47 | 16.79 | 1.686 |
| Q9NYL4 | Peptidyl-prolyl cis-trans isomerase FKBP11 OS=Homo sapiens GN=FKBP11 PE=1 SV=1 - [FKB11_HUMAN] | 50.96 | 8.96 | 1.684 |
| Q4G0N4 | NAD kinase 2, mitochondrial OS=Homo sapiens GN=NADK2 PE=1 SV=2 - [NAKD2_HUMAN] | 294.79 | 28.96 | 1.684 |
| Q9NVV5 | Androgen-induced gene 1 protein OS=Homo sapiens GN=AIG1 PE=1 SV=2 - [AIG1_HUMAN] | 72.72 | 6.12 | 1.683 |
| Q9BS16 | Centromere protein K OS=Homo sapiens GN=CENPK PE=1 SV=1 - [CENPK_HUMAN] | 0 | 2.97 | 1.681 |
| Q9BSY4 | Coiled-coil-helix-coiled-coil-helix domain-containing protein 5 OS=Homo sapiens GN=CHCHD5 PE=1 SV=1 - [CHCH5_HUMAN] | 74.79 | 24.55 | 1.679 |
| Q13464 | Rho-associated protein kinase 1 OS=Homo sapiens GN=ROCK1 PE=1 SV=1 - [ROCK1_HUMAN] | 463.4 | 29.91 | 1.677 |
| P35900 | Keratin, type I cytoskeletal 20 OS=Homo sapiens GN=KRT20 PE=1 SV=1 - [K1C20_HUMAN] | 23.46 | 3.75 | 1.677 |
| Q13099 | Intraflagellar transport protein 88 homolog OS=Homo sapiens GN=IFT88 PE=2 SV=2 - [IFT88_HUMAN] | 28.59 | 0.84 | 1.673 |
| Q9Y2V2 | Calcium-regulated heat-stable protein 1 OS=Homo sapiens GN=CARHSP1 PE=1 SV=2 - [CHSP1_HUMAN] | 119.87 | 38.78 | 1.672 |
| P15104 | Glutamine synthetase OS=Homo sapiens GN=GLUL PE=1 SV=4 - [GLNA_HUMAN] | 78.43 | 12.06 | 1.671 |
| Q8N6N3 | UPF0690 protein C1orf52 OS=Homo sapiens GN=C1orf52 PE=1 SV=1 - [CA052_HUMAN] | 54.33 | 35.71 | 1.665 |
| Q9H244 | P2Y purinoceptor 12 OS=Homo sapiens GN=P2RY12 PE=1 SV=1 - [P2Y12_HUMAN] | 32.58 | 4.09 | 1.664 |
| Q9UIS9 | Methyl-CpG-binding domain protein 1 OS=Homo sapiens GN=MBD1 PE=1 SV=2 - [MBD1_HUMAN] | 81.56 | 6.12 | 1.66 |
| P33240 | Cleavage stimulation factor subunit 2 OS=Homo sapiens GN=CSTF2 PE=1 SV=1 - [CSTF2_HUMAN] | 522.64 | 36.92 | 1.658 |
| Q9UI09 | NADH dehydrogenase [ubiquinone] 1 alpha subcomplex subunit 12 OS=Homo sapiens GN=NDUFA12 PE=1 SV=1 - [NDUAC_HUMAN] | 144.87 | 56.55 | 1.657 |
| Q9BQ70 | Transcription factor 25 OS=Homo sapiens GN=TCF25 PE=1 SV=1 - [TCF25_HUMAN] | 91.86 | 9.02 | 1.654 |
| P10515 | Dihydrolipoyllysine-residue acetyltransferase component of pyruvate dehydrogenase complex, mitochondrial OS=Homo sapiens GN=DLAT PE=1 SV=3 - [ODP2_HUMAN] | 929.61 | 28.75 | 1.652 |
| Q96EE4 | Coiled-coil domain-containing protein 126 OS=Homo sapiens GN=CCDC126 PE=2 SV=2 - [CC126_HUMAN] | 43.11 | 10.71 | 1.651 |
| P11310 | Medium-chain specific acyl-CoA dehydrogenase, mitochondrial OS=Homo sapiens GN=ACADM PE=1 SV=1 - [ACADM_HUMAN] | 979.55 | 44.18 | 1.651 |
| Q5SYC1 | Clavesin-2 OS=Homo sapiens GN=CLVS2 PE=2 SV=1 - [CLVS2_HUMAN] | 52.4 | 7.65 | 1.647 |
| Q15417 | Calponin-3 OS=Homo sapiens GN=CNN3 PE=1 SV=1 - [CNN3_HUMAN] | 917.79 | 54.41 | 1.644 |
| P52569 | Cationic amino acid transporter 2 OS=Homo sapiens GN=SLC7A2 PE=1 SV=2 - [CTR2_HUMAN] | 122.56 | 7.9 | 1.642 |
| O75962 | Triple functional domain protein OS=Homo sapiens GN=TRIO PE=1 SV=2 - [TRIO_HUMAN] | 41.01 | 3.13 | 1.639 |
| Q9HAV0 | Guanine nucleotide-binding protein subunit beta-4 OS=Homo sapiens GN=GNB4 PE=1 SV=3 - [GBB4_HUMAN] | 243.79 | 17.65 | 1.638 |
| P07858 | Cathepsin B OS=Homo sapiens GN=CTSB PE=1 SV=3 - [CATB_HUMAN] | 293.55 | 25.37 | 1.638 |
| Q8WU10 | Pyridine nucleotide-disulfide oxidoreductase domain-containing protein 1 OS=Homo sapiens GN=PYROXD1 PE=1 SV=1 - [PYRD1_HUMAN] | 58.21 | 2 | 1.637 |
| P15923 | Transcription factor E2-alpha OS=Homo sapiens GN=TCF3 PE=1 SV=1 - [TFE2_HUMAN] | 54.4 | 2.91 | 1.636 |
| Q9Y5X2 | Sorting nexin-8 OS=Homo sapiens GN=SNX8 PE=1 SV=1 - [SNX8_HUMAN] | 171.32 | 25.59 | 1.632 |
| Q6PCB8 | Embigin OS=Homo sapiens GN=EMB PE=1 SV=1 - [EMB_HUMAN] | 25.08 | 2.75 | 1.63 |
| Q86SQ7 | Serologically defined colon cancer antigen 8 OS=Homo sapiens GN=SDCCAG8 PE=1 SV=1 - [SDCG8_HUMAN] | 29.07 | 3.65 | 1.625 |
| P0CAP2 | DNA-directed RNA polymerase II subunit GRINL1A OS=Homo sapiens GN=POLR2M PE=1 SV=1 - [GRL1A_HUMAN] | 24.32 | 2.99 | 1.625 |
| Q9H5H4 | Zinc finger protein 768 OS=Homo sapiens GN=ZNF768 PE=1 SV=2 - [ZN768_HUMAN] | 67.6 | 13.33 | 1.624 |
| P36955 | Pigment epithelium-derived factor OS=Homo sapiens GN=SERPINF1 PE=1 SV=4 - [PEDF_HUMAN] | 0 | 2.15 | 1.622 |
| Q9BQ39 | ATP-dependent RNA helicase DDX50 OS=Homo sapiens GN=DDX50 PE=1 SV=1 - [DDX50_HUMAN] | 291.34 | 18.59 | 1.621 |
| P55347 | Homeobox protein PKNOX1 OS=Homo sapiens GN=PKNOX1 PE=1 SV=3 - [PKNX1_HUMAN] | 0 | 2.06 | 1.621 |
| O75843 | AP-1 complex subunit gamma-like 2 OS=Homo sapiens GN=AP1G2 PE=1 SV=1 - [AP1G2_HUMAN] | 21.75 | 0.76 | 1.619 |
| Q9H0W9 | Ester hydrolase C11orf54 OS=Homo sapiens GN=C11orf54 PE=1 SV=1 - [CK054_HUMAN] | 228.29 | 17.46 | 1.615 |
| P02652 | Apolipoprotein A-II OS=Homo sapiens GN=APOA2 PE=1 SV=1 - [APOA2_HUMAN] | 33.9 | 17 | 1.612 |
| P22415 | Upstream stimulatory factor 1 OS=Homo sapiens GN=USF1 PE=1 SV=1 - [USF1_HUMAN] | 47.92 | 6.13 | 1.611 |
| Q02252 | Methylmalonate-semialdehyde dehydrogenase [acylating], mitochondrial OS=Homo sapiens GN=ALDH6A1 PE=1 SV=2 - [MMSA_HUMAN] | 106.06 | 13.46 | 1.61 |
| Q96F07 | Cytoplasmic FMR1-interacting protein 2 OS=Homo sapiens GN=CYFIP2 PE=1 SV=2 - [CYFP2_HUMAN] | 339.14 | 14.71 | 1.608 |
| O60476 | Mannosyl-oligosaccharide 1,2-alpha-mannosidase IB OS=Homo sapiens GN=MAN1A2 PE=1 SV=1 - [MA1A2_HUMAN] | 77.67 | 8.89 | 1.608 |
| Q9NR30 | Nucleolar RNA helicase 2 OS=Homo sapiens GN=DDX21 PE=1 SV=5 - [DDX21_HUMAN] | 2661.94 | 49.04 | 1.607 |
| O95298 | NADH dehydrogenase [ubiquinone] 1 subunit C2 OS=Homo sapiens GN=NDUFC2 PE=1 SV=1 - [NDUC2_HUMAN] | 49.05 | 36.13 | 1.605 |
| O43674 | NADH dehydrogenase [ubiquinone] 1 beta subcomplex subunit 5, mitochondrial OS=Homo sapiens GN=NDUFB5 PE=1 SV=1 - [NDUB5_HUMAN] | 154.89 | 21.16 | 1.603 |
| Q99523 | Sortilin OS=Homo sapiens GN=SORT1 PE=1 SV=3 - [SORT_HUMAN] | 228.04 | 22.38 | 1.602 |
| Q8TCA0 | Leucine-rich repeat-containing protein 20 OS=Homo sapiens GN=LRRC20 PE=1 SV=1 - [LRC20_HUMAN] | 38.66 | 10.87 | 1.602 |
| P55789 | FAD-linked sulfhydryl oxidase ALR OS=Homo sapiens GN=GFER PE=1 SV=2 - [ALR_HUMAN] | 64.74 | 12.2 | 1.602 |
| Q7Z3T8 | Zinc finger FYVE domain-containing protein 16 OS=Homo sapiens GN=ZFYVE16 PE=1 SV=3 - [ZFY16_HUMAN] | 185.99 | 12.22 | 1.601 |
| Q6SJ93 | Protein FAM111B OS=Homo sapiens GN=FAM111B PE=1 SV=1 - [F111B_HUMAN] | 150.44 | 16.49 | 1.601 |
| P35558 | Phosphoenolpyruvate carboxykinase, cytosolic [GTP] OS=Homo sapiens GN=PCK1 PE=1 SV=3 - [PCKGC_HUMAN] | 195.31 | 9 | 1.601 |
| P11182 | Lipoamide acyltransferase component of branched-chain alpha-keto acid dehydrogenase complex, mitochondrial OS=Homo sapiens GN=DBT PE=1 SV=3 - [ODB2_HUMAN] | 162.45 | 21.37 | 1.601 |
| P00167 | Cytochrome b5 OS=Homo sapiens GN=CYB5A PE=1 SV=2 - [CYB5_HUMAN] | 444.76 | 57.46 | 1.601 |
| Q9UPU9 | Protein Smaug homolog 1 OS=Homo sapiens GN=SAMD4A PE=1 SV=3 - [SMAG1_HUMAN] | 38.21 | 2.37 | 1.599 |
| Q9H6D7 | HAUS augmin-like complex subunit 4 OS=Homo sapiens GN=HAUS4 PE=1 SV=1 - [HAUS4_HUMAN] | 47.56 | 7.44 | 1.599 |
| P08670 | Vimentin OS=Homo sapiens GN=VIM PE=1 SV=4 - [VIME_HUMAN] | 678.54 | 7.4 | 1.598 |
| P13051 | Uracil-DNA glycosylase OS=Homo sapiens GN=UNG PE=1 SV=2 - [UNG_HUMAN] | 88.9 | 13.74 | 1.597 |
| Q5T2D3 | OTU domain-containing protein 3 OS=Homo sapiens GN=OTUD3 PE=1 SV=1 - [OTUD3_HUMAN] | 24.91 | 5.53 | 1.596 |
| Q2Q1W2 | E3 ubiquitin-protein ligase TRIM71 OS=Homo sapiens GN=TRIM71 PE=1 SV=1 - [LIN41_HUMAN] | 73.21 | 5.41 | 1.596 |
| O43715 | TP53-regulated inhibitor of apoptosis 1 OS=Homo sapiens GN=TRIAP1 PE=1 SV=1 - [TRIA1_HUMAN] | 66.73 | 71.05 | 1.596 |
| P39060 | Collagen alpha-1(XVIII) chain OS=Homo sapiens GN=COL18A1 PE=1 SV=5 - [COIA1_HUMAN] | 55.94 | 2.05 | 1.595 |
| O14561 | Acyl carrier protein, mitochondrial OS=Homo sapiens GN=NDUFAB1 PE=1 SV=3 - [ACPM_HUMAN] | 74.78 | 14.74 | 1.595 |
| O95182 | NADH dehydrogenase [ubiquinone] 1 alpha subcomplex subunit 7 OS=Homo sapiens GN=NDUFA7 PE=1 SV=3 - [NDUA7_HUMAN] | 68.23 | 53.1 | 1.594 |
| Q9UH03 | Neuronal-specific septin-3 OS=Homo sapiens GN=SEPT3 PE=1 SV=3 - [SEPT3_HUMAN] | 26.4 | 2.51 | 1.593 |
| Q8N9F7 | Lysophospholipase D GDPD1 OS=Homo sapiens GN=GDPD1 PE=1 SV=2 - [GDPD1_HUMAN] | 120.91 | 20.7 | 1.593 |
| Q7Z6K3 | Protein prenyltransferase alpha subunit repeat-containing protein 1 OS=Homo sapiens GN=PTAR1 PE=1 SV=2 - [PTAR1_HUMAN] | 34.97 | 9.2 | 1.593 |
| Q16777 | Histone H2A type 2-C OS=Homo sapiens GN=HIST2H2AC PE=1 SV=4 - [H2A2C_HUMAN] | 242.67 | 39.91 | 1.591 |
| Q14195 | Dihydropyrimidinase-related protein 3 OS=Homo sapiens GN=DPYSL3 PE=1 SV=1 - [DPYL3_HUMAN] | 40.52 | 4.39 | 1.59 |
| P30405 | Peptidyl-prolyl cis-trans isomerase F, mitochondrial OS=Homo sapiens GN=PPIF PE=1 SV=1 - [PPIF_HUMAN] | 145.09 | 45.89 | 1.59 |
| Q9UPY8 | Microtubule-associated protein RP/EB family member 3 OS=Homo sapiens GN=MAPRE3 PE=1 SV=1 - [MARE3_HUMAN] | 89.02 | 8.9 | 1.585 |
| Q13576 | Ras GTPase-activating-like protein IQGAP2 OS=Homo sapiens GN=IQGAP2 PE=1 SV=4 - [IQGA2_HUMAN] | 658.86 | 26.41 | 1.585 |
| P07305 | Histone H1.0 OS=Homo sapiens GN=H1F0 PE=1 SV=3 - [H10_HUMAN] | 220.44 | 29.38 | 1.582 |
| P03915 | NADH-ubiquinone oxidoreductase chain 5 OS=Homo sapiens GN=MT-ND5 PE=1 SV=2 - [NU5M_HUMAN] | 29.29 | 2.82 | 1.582 |
| Q5SW96 | Low density lipoprotein receptor adapter protein 1 OS=Homo sapiens GN=LDLRAP1 PE=1 SV=3 - [ARH_HUMAN] | 0 | 3.25 | 1.581 |
| Q9UEE9 | Craniofacial development protein 1 OS=Homo sapiens GN=CFDP1 PE=1 SV=1 - [CFDP1_HUMAN] | 89.08 | 25.75 | 1.58 |
| Q15942 | Zyxin OS=Homo sapiens GN=ZYX PE=1 SV=1 - [ZYX_HUMAN] | 500.81 | 27.8 | 1.578 |
| Q03135 | Caveolin-1 OS=Homo sapiens GN=CAV1 PE=1 SV=4 - [CAV1_HUMAN] | 70.31 | 6.96 | 1.578 |
| Q7KZN9 | Cytochrome c oxidase assembly protein COX15 homolog OS=Homo sapiens GN=COX15 PE=1 SV=1 - [COX15_HUMAN] | 109.4 | 14.39 | 1.575 |
| Q96IZ0 | PRKC apoptosis WT1 regulator protein OS=Homo sapiens GN=PAWR PE=1 SV=1 - [PAWR_HUMAN] | 585.94 | 34.41 | 1.573 |
| P35520 | Cystathionine beta-synthase OS=Homo sapiens GN=CBS PE=1 SV=2 - [CBS_HUMAN] | 254.1 | 21.78 | 1.573 |
| P31431 | Syndecan-4 OS=Homo sapiens GN=SDC4 PE=1 SV=2 - [SDC4_HUMAN] | 61.71 | 17.68 | 1.573 |
| P02655 | Apolipoprotein C-II OS=Homo sapiens GN=APOC2 PE=1 SV=1 - [APOC2_HUMAN] | 44.14 | 8.91 | 1.573 |
| O00220 | Tumor necrosis factor receptor superfamily member 10A OS=Homo sapiens GN=TNFRSF10A PE=1 SV=3 - [TR10A_HUMAN] | 23.03 | 3.42 | 1.571 |
| Q9Y2F5 | Little elongation complex subunit 1 OS=Homo sapiens GN=ICE1 PE=1 SV=5 - [ICE1_HUMAN] | 48.44 | 1.15 | 1.569 |
| Q96EK5 | KIF1-binding protein OS=Homo sapiens GN=KIF1BP PE=1 SV=1 - [KBP_HUMAN] | 124.61 | 23.99 | 1.569 |
| P07919 | Cytochrome b-c1 complex subunit 6, mitochondrial OS=Homo sapiens GN=UQCRH PE=1 SV=2 - [QCR6_HUMAN] | 89.57 | 52.75 | 1.567 |
| Q15274 | Nicotinate-nucleotide pyrophosphorylase [carboxylating] OS=Homo sapiens GN=QPRT PE=1 SV=3 - [NADC_HUMAN] | 329.39 | 28.62 | 1.566 |
| Q6PD74 | Alpha- and gamma-adaptin-binding protein p34 OS=Homo sapiens GN=AAGAB PE=1 SV=1 - [AAGAB_HUMAN] | 50.25 | 15.87 | 1.562 |
| Q9UDY2 | Tight junction protein ZO-2 OS=Homo sapiens GN=TJP2 PE=1 SV=2 - [ZO2_HUMAN] | 676.48 | 27.56 | 1.561 |
| Q8WUK0 | Phosphatidylglycerophosphatase and protein-tyrosine phosphatase 1 OS=Homo sapiens GN=PTPMT1 PE=1 SV=1 - [PTPM1_HUMAN] | 0 | 8.46 | 1.561 |
| Q9NS56 | E3 ubiquitin-protein ligase Topors OS=Homo sapiens GN=TOPORS PE=1 SV=1 - [TOPRS_HUMAN] | 48.11 | 1.44 | 1.56 |
| Q9H2T7 | Ran-binding protein 17 OS=Homo sapiens GN=RANBP17 PE=2 SV=1 - [RBP17_HUMAN] | 30.28 | 0.74 | 1.56 |
| P42773 | Cyclin-dependent kinase 4 inhibitor C OS=Homo sapiens GN=CDKN2C PE=1 SV=1 - [CDN2C_HUMAN] | 0 | 4.17 | 1.56 |
| P19022 | Cadherin-2 OS=Homo sapiens GN=CDH2 PE=1 SV=4 - [CADH2_HUMAN] | 80.4 | 4.86 | 1.56 |
| Q9H9J4 | Ubiquitin carboxyl-terminal hydrolase 42 OS=Homo sapiens GN=USP42 PE=1 SV=3 - [UBP42_HUMAN] | 30.47 | 2.57 | 1.558 |
| Q96S15 | GATOR complex protein WDR24 OS=Homo sapiens GN=WDR24 PE=1 SV=1 - [WDR24_HUMAN] | 27.1 | 0.87 | 1.558 |
| Q08257 | Quinone oxidoreductase OS=Homo sapiens GN=CRYZ PE=1 SV=1 - [QOR_HUMAN] | 2392.66 | 70.52 | 1.556 |
| Q8WXF7 | Atlastin-1 OS=Homo sapiens GN=ATL1 PE=1 SV=1 - [ATLA1_HUMAN] | 43.34 | 4.66 | 1.555 |
| Q9UBV7 | Beta-1,4-galactosyltransferase 7 OS=Homo sapiens GN=B4GALT7 PE=1 SV=1 - [B4GT7_HUMAN] | 25.12 | 3.36 | 1.554 |
| Q9NZW5 | MAGUK p55 subfamily member 6 OS=Homo sapiens GN=MPP6 PE=1 SV=2 - [MPP6_HUMAN] | 187.5 | 25.37 | 1.553 |
| Q8IXY8 | Peptidyl-prolyl cis-trans isomerase-like 6 OS=Homo sapiens GN=PPIL6 PE=2 SV=1 - [PPIL6_HUMAN] | 30.46 | 2.57 | 1.552 |
| Q32P51 | Heterogeneous nuclear ribonucleoprotein A1-like 2 OS=Homo sapiens GN=HNRNPA1L2 PE=2 SV=2 - [RA1L2_HUMAN] | 3812.6 | 37.19 | 1.552 |
| P14406 | Cytochrome c oxidase subunit 7A2, mitochondrial OS=Homo sapiens GN=COX7A2 PE=1 SV=1 - [CX7A2_HUMAN] | 95.35 | 27.71 | 1.55 |
| O75380 | NADH dehydrogenase [ubiquinone] iron-sulfur protein 6, mitochondrial OS=Homo sapiens GN=NDUFS6 PE=1 SV=1 - [NDUS6_HUMAN] | 66.63 | 26.61 | 1.55 |
| Q9UBB4 | Ataxin-10 OS=Homo sapiens GN=ATXN10 PE=1 SV=1 - [ATX10_HUMAN] | 732.67 | 34.53 | 1.545 |
| P51114 | Fragile X mental retardation syndrome-related protein 1 OS=Homo sapiens GN=FXR1 PE=1 SV=3 - [FXR1_HUMAN] | 610.97 | 28.82 | 1.544 |
| Q9Y2B9 | cAMP-dependent protein kinase inhibitor gamma OS=Homo sapiens GN=PKIG PE=2 SV=1 - [IPKG_HUMAN] | 63.31 | 21.05 | 1.542 |
| P43363 | Melanoma-associated antigen 10 OS=Homo sapiens GN=MAGEA10 PE=2 SV=2 - [MAGAA_HUMAN] | 466.81 | 14.63 | 1.542 |
| Q8IUE0 | Homeobox protein TGIF2LY OS=Homo sapiens GN=TGIF2LY PE=1 SV=1 - [TF2LY_HUMAN] | 24.81 | 15.68 | 1.541 |
| Q15652 | Probable JmjC domain-containing histone demethylation protein 2C OS=Homo sapiens GN=JMJD1C PE=1 SV=2 - [JHD2C_HUMAN] | 46.85 | 1.89 | 1.54 |
| Q9H2A9 | Carbohydrate sulfotransferase 8 OS=Homo sapiens GN=CHST8 PE=1 SV=2 - [CHST8_HUMAN] | 15.8 | 1.42 | 1.539 |
| Q8NF91 | Nesprin-1 OS=Homo sapiens GN=SYNE1 PE=1 SV=4 - [SYNE1_HUMAN] | 0 | 0.3 | 1.537 |
| P53999 | Activated RNA polymerase II transcriptional coactivator p15 OS=Homo sapiens GN=SUB1 PE=1 SV=3 - [TCP4_HUMAN] | 480.83 | 77.95 | 1.537 |
| O15020 | Spectrin beta chain, non-erythrocytic 2 OS=Homo sapiens GN=SPTBN2 PE=1 SV=3 - [SPTN2_HUMAN] | 264.81 | 3.81 | 1.537 |
| P34931 | Heat shock 70 kDa protein 1-like OS=Homo sapiens GN=HSPA1L PE=1 SV=2 - [HS71L_HUMAN] | 2893.99 | 33.85 | 1.536 |
| Q96AH0 | SOSS complex subunit B2 OS=Homo sapiens GN=NABP1 PE=1 SV=1 - [SOSB2_HUMAN] | 29.01 | 9.8 | 1.535 |
| Q92990 | Glomulin OS=Homo sapiens GN=GLMN PE=1 SV=2 - [GLMN_HUMAN] | 254.7 | 15.32 | 1.535 |
| Q92538 | Golgi-specific brefeldin A-resistance guanine nucleotide exchange factor 1 OS=Homo sapiens GN=GBF1 PE=1 SV=2 - [GBF1_HUMAN] | 818.74 | 26.04 | 1.535 |
| P09630 | Homeobox protein Hox-C6 OS=Homo sapiens GN=HOXC6 PE=2 SV=3 - [HXC6_HUMAN] | 0 | 2.98 | 1.535 |
| Q9BW71 | HIRA-interacting protein 3 OS=Homo sapiens GN=HIRIP3 PE=1 SV=3 - [HIRP3_HUMAN] | 88.88 | 5.58 | 1.534 |
| Q15907 | Ras-related protein Rab-11B OS=Homo sapiens GN=RAB11B PE=1 SV=4 - [RB11B_HUMAN] | 1279.49 | 67.43 | 1.534 |
| P12429 | Annexin A3 OS=Homo sapiens GN=ANXA3 PE=1 SV=3 - [ANXA3_HUMAN] | 253.39 | 39.63 | 1.534 |
| Q53S58 | Transmembrane protein 177 OS=Homo sapiens GN=TMEM177 PE=2 SV=1 - [TM177_HUMAN] | 0 | 3.54 | 1.533 |
| Q9P260 | LisH domain and HEAT repeat-containing protein KIAA1468 OS=Homo sapiens GN=KIAA1468 PE=1 SV=2 - [K1468_HUMAN] | 239.86 | 16.04 | 1.532 |
| Q96IZ0 | PRKC apoptosis WT1 regulator protein OS=Homo sapiens GN=PAWR PE=1 SV=1 - [PAWR_HUMAN] | 585.94 | 34.41 | 1.532 |
| Q8NE62 | Choline dehydrogenase, mitochondrial OS=Homo sapiens GN=CHDH PE=1 SV=2 - [CHDH_HUMAN] | 113.87 | 23.91 | 1.532 |
| P20339 | Ras-related protein Rab-5A OS=Homo sapiens GN=RAB5A PE=1 SV=2 - [RAB5A_HUMAN] | 368.51 | 26.98 | 1.529 |
| Q9UK22 | F-box only protein 2 OS=Homo sapiens GN=FBXO2 PE=1 SV=2 - [FBX2_HUMAN] | 126.05 | 8.11 | 1.528 |
| Q9HCE1 | Putative helicase MOV-10 OS=Homo sapiens GN=MOV10 PE=1 SV=2 - [MOV10_HUMAN] | 356.31 | 16.95 | 1.525 |
| O60671 | Cell cycle checkpoint protein RAD1 OS=Homo sapiens GN=RAD1 PE=1 SV=1 - [RAD1_HUMAN] | 32.27 | 2.48 | 1.524 |
| Q96DE5 | Anaphase-promoting complex subunit 16 OS=Homo sapiens GN=ANAPC16 PE=1 SV=1 - [APC16_HUMAN] | 88.67 | 17.27 | 1.523 |
| Q9BXI6 | TBC1 domain family member 10A OS=Homo sapiens GN=TBC1D10A PE=1 SV=1 - [TB10A_HUMAN] | 74.37 | 11.02 | 1.519 |
| Q00978 | Interferon regulatory factor 9 OS=Homo sapiens GN=IRF9 PE=1 SV=1 - [IRF9_HUMAN] | 0 | 2.29 | 1.517 |
| O15239 | NADH dehydrogenase [ubiquinone] 1 alpha subcomplex subunit 1 OS=Homo sapiens GN=NDUFA1 PE=1 SV=1 - [NDUA1_HUMAN] | 31.82 | 8.57 | 1.517 |
| Q9BWF3 | RNA-binding protein 4 OS=Homo sapiens GN=RBM4 PE=1 SV=1 - [RBM4_HUMAN] | 764.64 | 51.65 | 1.515 |
| P16403 | Histone H1.2 OS=Homo sapiens GN=HIST1H1C PE=1 SV=2 - [H12_HUMAN] | 1793.23 | 47.89 | 1.515 |
| O60675 | Transcription factor MafK OS=Homo sapiens GN=MAFK PE=1 SV=1 - [MAFK_HUMAN] | 62.78 | 30.13 | 1.514 |
| Q9H2G2 | STE20-like serine/threonine-protein kinase OS=Homo sapiens GN=SLK PE=1 SV=1 - [SLK_HUMAN] | 818.2 | 36.84 | 1.513 |
| P07307 | Asialoglycoprotein receptor 2 OS=Homo sapiens GN=ASGR2 PE=1 SV=2 - [ASGR2_HUMAN] | 33.3 | 20.26 | 1.512 |
| Q92793 | CREB-binding protein OS=Homo sapiens GN=CREBBP PE=1 SV=3 - [CBP_HUMAN] | 99.35 | 2.83 | 1.509 |
| Q9P2J9 | [Pyruvate dehydrogenase [acetyl-transferring]]-phosphatase 2, mitochondrial OS=Homo sapiens GN=PDP2 PE=2 SV=2 - [PDP2_HUMAN] | 33.22 | 2.65 | 1.508 |
| Q5VZK9 | F-actin-uncapping protein LRRC16A OS=Homo sapiens GN=CARMIL1 PE=1 SV=1 - [CARL1_HUMAN] | 89.48 | 9.85 | 1.508 |
| Q9H2A7 | C-X-C motif chemokine 16 OS=Homo sapiens GN=CXCL16 PE=2 SV=4 - [CXL16_HUMAN] | 25.25 | 2.76 | 1.505 |
| Q5JSZ5 | Protein PRRC2B OS=Homo sapiens GN=PRRC2B PE=1 SV=2 - [PRC2B_HUMAN] | 83.07 | 4.62 | 1.505 |
| Q00534 | Cyclin-dependent kinase 6 OS=Homo sapiens GN=CDK6 PE=1 SV=1 - [CDK6_HUMAN] | 131.54 | 22.39 | 1.504 |
| Q13356 | Peptidyl-prolyl cis-trans isomerase-like 2 OS=Homo sapiens GN=PPIL2 PE=1 SV=1 - [PPIL2_HUMAN] | 66.26 | 12.5 | 1.503 |
| P18669 | Phosphoglycerate mutase 1 OS=Homo sapiens GN=PGAM1 PE=1 SV=2 - [PGAM1_HUMAN] | 2778.39 | 65.75 | 1.503 |
| P61163 | Alpha-centractin OS=Homo sapiens GN=ACTR1A PE=1 SV=1 - [ACTZ_HUMAN] | 611.26 | 35.37 | 1.502 |
| O15111 | Inhibitor of nuclear factor kappa-B kinase subunit alpha OS=Homo sapiens GN=CHUK PE=1 SV=2 - [IKKA_HUMAN] | 190.27 | 10.07 | 1.502 |
| O14757 | Serine/threonine-protein kinase Chk1 OS=Homo sapiens GN=CHEK1 PE=1 SV=2 - [CHK1_HUMAN] | 88.49 | 17.02 | 1.502 |
| Q93034 | Cullin-5 OS=Homo sapiens GN=CUL5 PE=1 SV=4 - [CUL5_HUMAN] | 287.91 | 21.92 | 1.5 |
| Q8NEK5 | Zinc finger protein 548 OS=Homo sapiens GN=ZNF548 PE=1 SV=2 - [ZN548_HUMAN] | 0 | 3.56 | 1.5 |
|  |  |  |  |  |
|  |  |  |  |  |
|  |  |  |  |  |
|  |  |  |  |  |
|  |  |  |  |  |
|  |  |  |  |  |
