# Supplementary material for: Overexpression of RNF38 facilitates TGF-β signaling by Ubiquitinating and degrading AHNAK in hepatocellular carcinoma
Source: J Exp Clin Cancer Res. 2019 Mar 5;38:113. doi: 10.1186/s13046-019-1113-3 (PMC6402116; doi:10.1186/s13046-019-1113-3)
Supplement: Supplementary file 8 — Table S3. SILAC of differential proteins in HepG2-RNF38 and HepG2-vector (Down-regulated) (DOCX 37 kb) [file 13046_2019_1113_MOESM8_ESM.docx]

Supplementary table 3. SILAC of differential proteins in HepG2-RNF38 and HepG2-vector (Down-regulated)

| Accession | Description | Score | Coverage | Fold change (≥1.5 times) |
| --- | --- | --- | --- | --- |
| P02675 | Variable charge X-linked protein 2 OS=Homo sapiens GN=VCX2 PE=2 SV=3 - [VCX2_HUMAN] | 50.18 | 7.91 | 28.57142857 |
| Q8TAD7 | Protein S100-P OS=Homo sapiens GN=S100P PE=1 SV=2 - [S100P_HUMAN] | 390.48 | 48.42 | 15.38461538 |
| Q8IVT2 | Kinesin-like protein KIF28P OS=Homo sapiens GN=KIF28P PE=3 SV=2 - [KIF28_HUMAN] | 0 | 1.45 | 12.65822785 |
| Q9UNQ0 | CD9 antigen OS=Homo sapiens GN=CD9 PE=1 SV=4 - [CD9_HUMAN] | 29.13 | 3.07 | 10.30927835 |
| P32455 | Cytochrome b-245 light chain OS=Homo sapiens GN=CYBA PE=1 SV=3 - [CY24A_HUMAN] | 41.86 | 15.9 | 10.1010101 |
| Q96BS2 | G antigen 2B/2C OS=Homo sapiens GN=GAGE2B PE=1 SV=1 - [GAG2B_HUMAN] | 25.41 | 6.03 | 9.174311927 |
| Q14CM0 | Tubulin alpha-4A chain OS=Homo sapiens GN=TUBA4A PE=1 SV=1 - [TBA4A_HUMAN] | 6415.87 | 51.56 | 9.009009009 |
| Q9C073 | Xin actin-binding repeat-containing protein 2 OS=Homo sapiens GN=XIRP2 PE=1 SV=2 - [XIRP2_HUMAN] | 26.26 | 0.47 | 7.8125 |
| P07195 | Annexin A13 OS=Homo sapiens GN=ANXA13 PE=1 SV=3 - [ANX13_HUMAN] | 32.62 | 10.76 | 6.896551724 |
| Q5VTQ0 | Histone H2B type 1-A OS=Homo sapiens GN=HIST1H2BA PE=1 SV=3 - [H2B1A_HUMAN] | 957.87 | 30.71 | 6.849315068 |
| P09972 | CD81 antigen OS=Homo sapiens GN=CD81 PE=1 SV=1 - [CD81_HUMAN] | 40 | 11.02 | 6.666666667 |
| P13796 | ATP-dependent 6-phosphofructokinase, muscle type OS=Homo sapiens GN=PFKM PE=1 SV=2 - [PFKAM_HUMAN] | 460.92 | 25.9 | 5.917159763 |
| P48681 | Mothers against decapentaplegic homolog 2 OS=Homo sapiens GN=SMAD2 PE=1 SV=1 - [SMAD2_HUMAN] | 76.73 | 16.92 | 5.813953488 |
| Q96B97 | Protein FAM9C OS=Homo sapiens GN=FAM9C PE=1 SV=1 - [FAM9C_HUMAN] | 0 | 7.83 | 5.464480874 |
| Q8IYD9 | 40S ribosomal protein S27-like OS=Homo sapiens GN=RPS27L PE=1 SV=3 - [RS27L_HUMAN] | 1660.13 | 58.09 | 5.376344086 |
| P49448 | Galectin-3-binding protein OS=Homo sapiens GN=LGALS3BP PE=1 SV=1 - [LG3BP_HUMAN] | 359.58 | 20.17 | 5.376344086 |
| P27216 | CD97 antigen OS=Homo sapiens GN=CD97 PE=1 SV=4 - [CD97_HUMAN] | 85.87 | 3.47 | 5.347593583 |
| Q7Z4F1 | Cathepsin S OS=Homo sapiens GN=CTSS PE=1 SV=3 - [CATS_HUMAN] | 57.84 | 8.16 | 5.102040816 |
| P00973 | Polypeptide N-acetylgalactosaminyltransferase 4 OS=Homo sapiens GN=GALNT4 PE=1 SV=2 - [GALT4_HUMAN] | 83.94 | 6.57 | 5.076142132 |
| P21333 | Thymosin beta-4 OS=Homo sapiens GN=TMSB4X PE=1 SV=2 - [TYB4_HUMAN] | 248.87 | 63.64 | 5 |
| P62760 | Dehydrogenase/reductase SDR family member 2, mitochondrial OS=Homo sapiens GN=DHRS2 PE=1 SV=4 - [DHRS2_HUMAN] | 22.88 | 2.5 | 4.901960784 |
| Q8WVV4 | HLA class I histocompatibility antigen, A-3 alpha chain OS=Homo sapiens GN=HLA-A PE=1 SV=2 - [1A03_HUMAN] | 515.15 | 46.3 | 4.87804878 |
| P04440 | Monocarboxylate transporter 4 OS=Homo sapiens GN=SLC16A3 PE=1 SV=1 - [MOT4_HUMAN] | 0 | 1.51 | 4.739336493 |
| P50281 | C-C motif chemokine 20 OS=Homo sapiens GN=CCL20 PE=1 SV=1 - [CCL20_HUMAN] | 27.91 | 8.33 | 4.716981132 |
| Q9NXG6 | Protein FAM111A OS=Homo sapiens GN=FAM111A PE=1 SV=2 - [F111A_HUMAN] | 31.11 | 3.27 | 4.694835681 |
| P29966 | Histone H2B type 1-A OS=Homo sapiens GN=HIST1H2BA PE=1 SV=3 - [H2B1A_HUMAN] | 957.87 | 30.71 | 4.504504505 |
| P50225 | 14-3-3 protein sigma OS=Homo sapiens GN=SFN PE=1 SV=1 - [1433S_HUMAN] | 845.98 | 64.52 | 4.444444444 |
| O95671 | NAD(P)H dehydrogenase [quinone] 1 OS=Homo sapiens GN=NQO1 PE=1 SV=1 - [NQO1_HUMAN] | 536.16 | 35.04 | 4.329004329 |
| P27338 | Ornithine carbamoyltransferase, mitochondrial OS=Homo sapiens GN=OTC PE=1 SV=3 - [OTC_HUMAN] | 41.17 | 2.82 | 4.329004329 |
| Q5TZA2 | RING finger protein 38 OS=Homo sapiens GN=RNF38 PE=1 SV=2 - [RNF38_HUMAN] | 220.52 | 19.35 | 4.310344828 |
| P02792 | Tetraspanin-7 OS=Homo sapiens GN=TSPAN7 PE=1 SV=2 - [TSN7_HUMAN] | 172.69 | 8.84 | 4.237288136 |
| Q14914 | Heat-stable enterotoxin receptor OS=Homo sapiens GN=GUCY2C PE=1 SV=2 - [GUC2C_HUMAN] | 0 | 0.56 | 4.184100418 |
| Q96GU1 | Palladin OS=Homo sapiens GN=PALLD PE=1 SV=3 - [PALLD_HUMAN] | 397.82 | 9.26 | 4.166666667 |
| Q9NZR1 | Corticoliberin OS=Homo sapiens GN=CRH PE=1 SV=1 - [CRF_HUMAN] | 133.9 | 15.82 | 4.132231405 |
| P54868 | Carbamoyl-phosphate synthase [ammonia], mitochondrial OS=Homo sapiens GN=CPS1 PE=1 SV=2 - [CPSM_HUMAN] | 9086.96 | 54.67 | 4.098360656 |
| O00499 | Protein PBDC1 OS=Homo sapiens GN=PBDC1 PE=1 SV=1 - [PBDC1_HUMAN] | 32.91 | 17.6 | 4.081632653 |
| P07148 | MFS-type transporter SLC18B1 OS=Homo sapiens GN=SLC18B1 PE=1 SV=1 - [S18B1_HUMAN] | 51.97 | 2.41 | 4.081632653 |
| Q96BW5 | Bone marrow stromal antigen 2 OS=Homo sapiens GN=BST2 PE=1 SV=1 - [BST2_HUMAN] | 61.94 | 21.67 | 3.891050584 |
| Q6NZI2 | Calbindin OS=Homo sapiens GN=CALB1 PE=1 SV=2 - [CALB1_HUMAN] | 292.89 | 43.68 | 3.861003861 |
| Q9HB65 | Unconventional myosin-Id OS=Homo sapiens GN=MYO1D PE=1 SV=2 - [MYO1D_HUMAN] | 358.7 | 29.92 | 3.846153846 |
| O00194 | Histone H2A type 2-C OS=Homo sapiens GN=HIST2H2AC PE=1 SV=4 - [H2A2C_HUMAN] | 242.67 | 39.91 | 3.831417625 |
| O00625 | Myeloid differentiation primary response protein MyD88 OS=Homo sapiens GN=MYD88 PE=1 SV=1 - [MYD88_HUMAN] | 0 | 7.09 | 3.816793893 |
| P21589 | Translocator protein OS=Homo sapiens GN=TSPO PE=1 SV=3 - [TSPO_HUMAN] | 65.56 | 9.47 | 3.816793893 |
| P10620 | Aminopeptidase N OS=Homo sapiens GN=ANPEP PE=1 SV=4 - [AMPN_HUMAN] | 1157.04 | 32.26 | 3.816793893 |
| P00450 | Low-density lipoprotein receptor-related protein 2 OS=Homo sapiens GN=LRP2 PE=1 SV=3 - [LRP2_HUMAN] | 72.47 | 16.22 | 3.802281369 |
| P01031 | Platelet endothelial cell adhesion molecule OS=Homo sapiens GN=PECAM1 PE=1 SV=1 - [PECA1_HUMAN] | 24.37 | 1.22 | 3.731343284 |
| O75843 | UDP-glucuronosyltransferase 1-1 OS=Homo sapiens GN=UGT1A1 PE=1 SV=1 - [UD11_HUMAN] | 251.82 | 15.76 | 3.717472119 |
| Q9BVA1 | Anterior gradient protein 2 homolog OS=Homo sapiens GN=AGR2 PE=1 SV=1 - [AGR2_HUMAN] | 1688.89 | 68 | 3.717472119 |
| Q05682 | Four and a half LIM domains protein 1 OS=Homo sapiens GN=FHL1 PE=1 SV=4 - [FHL1_HUMAN] | 52.91 | 3.72 | 3.6900369 |
| P54868 | Immortalization up-regulated protein OS=Homo sapiens GN=IMUP PE=1 SV=1 - [IMUP_HUMAN] | 32.54 | 16.04 | 3.676470588 |
| P78545 | Breast carcinoma-amplified sequence 1 OS=Homo sapiens GN=BCAS1 PE=1 SV=2 - [BCAS1_HUMAN] | 630.33 | 45.72 | 3.649635036 |
| Q96T17 | STE20/SPS1-related proline-alanine-rich protein kinase OS=Homo sapiens GN=STK39 PE=1 SV=3 - [STK39_HUMAN] | 0 | 2.57 | 3.636363636 |
| Q96EB6 | Perilipin-2 OS=Homo sapiens GN=PLIN2 PE=1 SV=2 - [PLIN2_HUMAN] | 236.43 | 18.31 | 3.636363636 |
| P18283 | Protein SSX2 OS=Homo sapiens GN=SSX2 PE=1 SV=2 - [SSX2_HUMAN] | 161.44 | 30.32 | 3.623188406 |
| O60879 | Fibrinogen gamma chain OS=Homo sapiens GN=FGG PE=1 SV=3 - [FIBG_HUMAN] | 833.93 | 39.51 | 3.496503497 |
| Q9Y624 | Kynureninase OS=Homo sapiens GN=KYNU PE=1 SV=1 - [KYNU_HUMAN] | 638.68 | 37.2 | 3.412969283 |
| P14209 | Mucin-2 OS=Homo sapiens GN=MUC2 PE=1 SV=2 - [MUC2_HUMAN] | 107.94 | 3.24 | 3.367003367 |
| P02787 | Glucose-6-phosphate 1-dehydrogenase OS=Homo sapiens GN=G6PD PE=1 SV=4 - [G6PD_HUMAN] | 1178.71 | 40.58 | 3.367003367 |
| Q9HAU4 | Protein S100-A11 OS=Homo sapiens GN=S100A11 PE=1 SV=2 - [S10AB_HUMAN] | 308.39 | 39.05 | 3.333333333 |
| Q3SY69 | Fibrinogen alpha chain OS=Homo sapiens GN=FGA PE=1 SV=2 - [FIBA_HUMAN] | 2471.5 | 39.49 | 3.322259136 |
| Q07065 | Proline-serine-threonine phosphatase-interacting protein 2 OS=Homo sapiens GN=PSTPIP2 PE=1 SV=4 - [PPIP2_HUMAN] | 34.76 | 4.19 | 3.300330033 |
| P48995 | Tyrosine-protein kinase FRK OS=Homo sapiens GN=FRK PE=1 SV=1 - [FRK_HUMAN] | 27.04 | 4.75 | 3.300330033 |
| Q14766 | Tetraspanin-8 OS=Homo sapiens GN=TSPAN8 PE=1 SV=1 - [TSN8_HUMAN] | 108.85 | 8.86 | 3.300330033 |
| P15882 | Probable glutathione peroxidase 8 OS=Homo sapiens GN=GPX8 PE=1 SV=2 - [GPX8_HUMAN] | 94.51 | 8.61 | 3.267973856 |
| Q13145 | Villin-1 OS=Homo sapiens GN=VIL1 PE=1 SV=4 - [VILI_HUMAN] | 1395.66 | 44.26 | 3.236245955 |
| Q86SF2 | Tropomyosin alpha-4 chain OS=Homo sapiens GN=TPM4 PE=1 SV=3 - [TPM4_HUMAN] | 1647.75 | 75 | 3.225806452 |
| Q8N8V4 | Protein S100-A14 OS=Homo sapiens GN=S100A14 PE=1 SV=1 - [S10AE_HUMAN] | 96.74 | 21.15 | 3.205128205 |
| Q96S97 | Protein-glutamine gamma-glutamyltransferase 2 OS=Homo sapiens GN=TGM2 PE=1 SV=2 - [TGM2_HUMAN] | 567.41 | 31.59 | 3.194888179 |
| Q96HE7 | C-terminal-binding protein 2 OS=Homo sapiens GN=CTBP2 PE=1 SV=1 - [CTBP2_HUMAN] | 136.41 | 13.03 | 3.184713376 |
| Q9H3R2 | TBC1 domain family member 4 OS=Homo sapiens GN=TBC1D4 PE=1 SV=2 - [TBCD4_HUMAN] | 38.6 | 4.01 | 3.184713376 |
| P84022 | 1,25-dihydroxyvitamin D(3) 24-hydroxylase, mitochondrial OS=Homo sapiens GN=CYP24A1 PE=1 SV=2 - [CP24A_HUMAN] | 154.62 | 18.87 | 3.174603175 |
| Q96A49 | Filamin-binding LIM protein 1 OS=Homo sapiens GN=FBLIM1 PE=1 SV=2 - [FBLI1_HUMAN] | 50.29 | 4.56 | 3.164556962 |
| Q8N0U4 | Clusterin OS=Homo sapiens GN=CLU PE=1 SV=1 - [CLUS_HUMAN] | 282.09 | 22.49 | 3.164556962 |
| P32121 | Caveolae-associated protein 2 OS=Homo sapiens GN=CAVIN2 PE=1 SV=3 - [CAVN2_HUMAN] | 378.44 | 36 | 3.154574132 |
| Q969P0 | Overexpressed in colon carcinoma 1 protein OS=Homo sapiens GN=OCC1 PE=1 SV=2 - [OCC1_HUMAN] | 50.96 | 19.05 | 3.125 |
| P49184 | Fibrinogen beta chain OS=Homo sapiens GN=FGB PE=1 SV=2 - [FIBB_HUMAN] | 448.9 | 36.25 | 3.125 |
| Q13485 | ATP-binding cassette sub-family G member 2 OS=Homo sapiens GN=ABCG2 PE=1 SV=3 - [ABCG2_HUMAN] | 82.36 | 5.34 | 3.115264798 |
| P23443 | Mitotic interactor and substrate of PLK1 OS=Homo sapiens GN=MISP PE=1 SV=1 - [MISP_HUMAN] | 468.06 | 34.9 | 3.115264798 |
| Q96SQ9 | Guanylate-binding protein 1 OS=Homo sapiens GN=GBP1 PE=1 SV=2 - [GBP1_HUMAN] | 94.39 | 9.8 | 3.095975232 |
| P01008 | Calcineurin B homologous protein 3 OS=Homo sapiens GN=TESC PE=1 SV=3 - [CHP3_HUMAN] | 104.91 | 25.7 | 3.067484663 |
| P25942 | FERM and PDZ domain-containing protein 4 OS=Homo sapiens GN=FRMPD4 PE=1 SV=1 - [FRPD4_HUMAN] | 28.93 | 0.98 | 3.012048193 |
| P30837 | Protein FAM117A OS=Homo sapiens GN=FAM117A PE=1 SV=1 - [F117A_HUMAN] | 33.57 | 1.55 | 2.994011976 |
| P63313 | Tetratricopeptide repeat protein 39B OS=Homo sapiens GN=TTC39B PE=1 SV=4 - [TT39B_HUMAN] | 47.27 | 1.76 | 2.976190476 |
| Q14573 | L-lactate dehydrogenase B chain OS=Homo sapiens GN=LDHB PE=1 SV=2 - [LDHB_HUMAN] | 1808.69 | 44.61 | 2.976190476 |
| P50851 | Fructose-bisphosphate aldolase C OS=Homo sapiens GN=ALDOC PE=1 SV=2 - [ALDOC_HUMAN] | 856.66 | 50.82 | 2.941176471 |
| O95571 | Plastin-2 OS=Homo sapiens GN=LCP1 PE=1 SV=6 - [PLSL_HUMAN] | 1902.55 | 56.94 | 2.93255132 |
| Q9Y2Q3 | SH3 domain-containing kinase-binding protein 1 OS=Homo sapiens GN=SH3KBP1 PE=1 SV=2 - [SH3K1_HUMAN] | 117.15 | 11.58 | 2.923976608 |
| P09104 | Nestin OS=Homo sapiens GN=NES PE=1 SV=2 - [NEST_HUMAN] | 208.8 | 13.7 | 2.923976608 |
| Q9C030 | Glutamate dehydrogenase 2, mitochondrial OS=Homo sapiens GN=GLUD2 PE=1 SV=2 - [DHE4_HUMAN] | 1389.22 | 31 | 2.915451895 |
| P00325 | Lung adenoma susceptibility protein 2 OS=Homo sapiens GN=LAS2 PE=1 SV=1 - [LAS2_HUMAN] | 0 | 2.15 | 2.88184438 |
| P55011 | Annexin A13 OS=Homo sapiens GN=ANXA13 PE=1 SV=3 - [ANX13_HUMAN] | 32.62 | 10.76 | 2.873563218 |
| P62877 | Low-density lipoprotein receptor-related protein 10 OS=Homo sapiens GN=LRP10 PE=1 SV=2 - [LRP10_HUMAN] | 33.3 | 2.95 | 2.865329513 |
| P52788 | 2'-5'-oligoadenylate synthase 1 OS=Homo sapiens GN=OAS1 PE=1 SV=4 - [OAS1_HUMAN] | 31.78 | 4.5 | 2.857142857 |
| Q14511 | Filamin-A OS=Homo sapiens GN=FLNA PE=1 SV=4 - [FLNA_HUMAN] | 7601.42 | 53.83 | 2.840909091 |
| P16422 | Protein POF1B OS=Homo sapiens GN=POF1B PE=1 SV=3 - [POF1B_HUMAN] | 285.59 | 25.3 | 2.808988764 |
| Q9UHN1 | Visinin-like protein 1 OS=Homo sapiens GN=VSNL1 PE=1 SV=2 - [VISL1_HUMAN] | 37.63 | 17.8 | 2.808988764 |
| A6NCS6 | Transmembrane prolyl 4-hydroxylase OS=Homo sapiens GN=P4HTM PE=1 SV=2 - [P4HTM_HUMAN] | 47.97 | 6.18 | 2.801120448 |
| P19823 | Matrix metalloproteinase-14 OS=Homo sapiens GN=MMP14 PE=1 SV=3 - [MMP14_HUMAN] | 37.67 | 4.3 | 2.801120448 |
| Q15120 | HLA class II histocompatibility antigen, DP beta 1 chain OS=Homo sapiens GN=HLA-DPB1 PE=1 SV=1 - [DPB1_HUMAN] | 25.4 | 2.71 | 2.801120448 |
| Q8WTV0 | Sulfotransferase 1A1 OS=Homo sapiens GN=SULT1A1 PE=1 SV=3 - [ST1A1_HUMAN] | 155.82 | 27.12 | 2.793296089 |
| Q7Z3T8 | Myristoylated alanine-rich C-kinase substrate OS=Homo sapiens GN=MARCKS PE=1 SV=4 - [MARCS_HUMAN] | 3594.41 | 51.51 | 2.793296089 |
| Q6ZVF9 | Amine oxidase [flavin-containing] B OS=Homo sapiens GN=MAOB PE=1 SV=3 - [AOFB_HUMAN] | 153.33 | 13.46 | 2.777777778 |
| P17252 | N-acetylserotonin O-methyltransferase-like protein OS=Homo sapiens GN=ASMTL PE=1 SV=3 - [ASML_HUMAN] | 302.61 | 29.15 | 2.770083102 |
| Q13885 | Rootletin OS=Homo sapiens GN=CROCC PE=1 SV=1 - [CROCC_HUMAN] | 184.8 | 6.79 | 2.747252747 |
| Q96B70 | P antigen family member 5 OS=Homo sapiens GN=PAGE5 PE=1 SV=2 - [PAGE5_HUMAN] | 192.36 | 54.62 | 2.72479564 |
| P12259 | Prostaglandin reductase 1 OS=Homo sapiens GN=PTGR1 PE=1 SV=2 - [PTGR1_HUMAN] | 27.78 | 3.04 | 2.72479564 |
| P33908 | Ferritin light chain OS=Homo sapiens GN=FTL PE=1 SV=2 - [FRIL_HUMAN] | 107.29 | 27.43 | 2.72479564 |
| P30626 | Tropomodulin-2 OS=Homo sapiens GN=TMOD2 PE=1 SV=1 - [TMOD2_HUMAN] | 68.47 | 4.56 | 2.702702703 |
| Q8N5S9 | Hydroxymethylglutaryl-CoA synthase, mitochondrial OS=Homo sapiens GN=HMGCS2 PE=1 SV=1 - [HMCS2_HUMAN] | 81.34 | 9.06 | 2.69541779 |
| Q8WUH2 | Fatty acid-binding protein, liver OS=Homo sapiens GN=FABP1 PE=1 SV=1 - [FABPL_HUMAN] | 182.73 | 41.73 | 2.673796791 |
| Q86V59 | Myc box-dependent-interacting protein 1 OS=Homo sapiens GN=BIN1 PE=1 SV=1 - [BIN1_HUMAN] | 407.74 | 41.15 | 2.652519894 |
| Q9P2Q2 | Phosphotriesterase-related protein OS=Homo sapiens GN=PTER PE=1 SV=1 - [PTER_HUMAN] | 323.2 | 20.06 | 2.645502646 |
| P00742 | Caveolae-associated protein 1 OS=Homo sapiens GN=CAVIN1 PE=1 SV=1 - [CAVN1_HUMAN] | 327.02 | 35.9 | 2.624671916 |
| P12236 | RNA polymerase II elongation factor ELL3 OS=Homo sapiens GN=ELL3 PE=1 SV=2 - [ELL3_HUMAN] | 31.88 | 4.28 | 2.610966057 |
| Q92793 | Ras-related protein Rab-27B OS=Homo sapiens GN=RAB27B PE=1 SV=4 - [RB27B_HUMAN] | 233.61 | 17.89 | 2.604166667 |
| Q9Y680 | Microsomal glutathione S-transferase 1 OS=Homo sapiens GN=MGST1 PE=1 SV=1 - [MGST1_HUMAN] | 152.86 | 9.03 | 2.597402597 |
| P13667 | 5'-nucleotidase OS=Homo sapiens GN=NT5E PE=1 SV=1 - [5NTD_HUMAN] | 129.21 | 23.87 | 2.583979328 |
| P52209 | Pirin OS=Homo sapiens GN=PIR PE=1 SV=1 - [PIR_HUMAN] | 354.89 | 42.41 | 2.564102564 |
| Q9Y4H2 | Complement C5 OS=Homo sapiens GN=C5 PE=1 SV=4 - [CO5_HUMAN] | 83.7 | 3.34 | 2.551020408 |
| Q13501 | Ceruloplasmin OS=Homo sapiens GN=CP PE=1 SV=1 - [CERU_HUMAN] | 85.82 | 13.62 | 2.551020408 |
| Q96D15 | Tubulin beta-2B chain OS=Homo sapiens GN=TUBB2B PE=1 SV=1 - [TBB2B_HUMAN] | 5650.91 | 52.36 | 2.538071066 |
| P20337 | Caldesmon OS=Homo sapiens GN=CALD1 PE=1 SV=3 - [CALD1_HUMAN] | 1287.1 | 33.67 | 2.518891688 |
| O95025 | AP-1 complex subunit gamma-like 2 OS=Homo sapiens GN=AP1G2 PE=1 SV=1 - [AP1G2_HUMAN] | 21.75 | 0.76 | 2.518891688 |
| P37802 | ETS-related transcription factor Elf-3 OS=Homo sapiens GN=ELF3 PE=1 SV=1 - [ELF3_HUMAN] | 31.65 | 6.47 | 2.512562814 |
| P43304 | Hydroxymethylglutaryl-CoA synthase, mitochondrial OS=Homo sapiens GN=HMGCS2 PE=1 SV=1 - [HMCS2_HUMAN] | 81.34 | 9.06 | 2.512562814 |
| Q02318 | NAD-dependent protein deacetylase sirtuin-1 OS=Homo sapiens GN=SIRT1 PE=1 SV=2 - [SIR1_HUMAN] | 68.24 | 8.99 | 2.506265664 |
| Q15942 | MAP7 domain-containing protein 2 OS=Homo sapiens GN=MAP7D2 PE=1 SV=2 - [MA7D2_HUMAN] | 255.7 | 14.34 | 2.5 |
| P49407 | Glutathione peroxidase 2 OS=Homo sapiens GN=GPX2 PE=1 SV=3 - [GPX2_HUMAN] | 37.81 | 12.11 | 2.475247525 |
| Q99674 | Junctional adhesion molecule A OS=Homo sapiens GN=F11R PE=1 SV=1 - [JAM1_HUMAN] | 227.18 | 41.14 | 2.450980392 |
| Q6ZTQ3 | Protein diaphanous homolog 2 OS=Homo sapiens GN=DIAPH2 PE=1 SV=1 - [DIAP2_HUMAN] | 46.29 | 3.72 | 2.450980392 |
| O15400 | CD99 antigen OS=Homo sapiens GN=CD99 PE=1 SV=1 - [CD99_HUMAN] | 306.68 | 18.38 | 2.444987775 |
| O14832 | Serotransferrin OS=Homo sapiens GN=TF PE=1 SV=3 - [TRFE_HUMAN] | 288.67 | 27.08 | 2.444987775 |
| Q9H4G0 | E3 ubiquitin-protein ligase SMURF2 OS=Homo sapiens GN=SMURF2 PE=1 SV=1 - [SMUF2_HUMAN] | 31.4 | 1.2 | 2.433090024 |
| Q14449 | Mitochondrial 10-formyltetrahydrofolate dehydrogenase OS=Homo sapiens GN=ALDH1L2 PE=1 SV=2 - [AL1L2_HUMAN] | 0 | 0.87 | 2.421307506 |
| Q12797 | Latent-transforming growth factor beta-binding protein 1 OS=Homo sapiens GN=LTBP1 PE=1 SV=4 - [LTBP1_HUMAN] | 33.68 | 0.58 | 2.415458937 |
| Q15758 | Short transient receptor potential channel 1 OS=Homo sapiens GN=TRPC1 PE=1 SV=1 - [TRPC1_HUMAN] | 33.62 | 1.64 | 2.409638554 |
| Q9C037 | Cytoskeleton-associated protein 4 OS=Homo sapiens GN=CKAP4 PE=1 SV=2 - [CKAP4_HUMAN] | 2587.99 | 69.44 | 2.403846154 |
| O95405 | N-acetylgalactosaminyltransferase 7 OS=Homo sapiens GN=GALNT7 PE=1 SV=1 - [GALT7_HUMAN] | 280.18 | 19.79 | 2.392344498 |
| O43167 | BMP and activin membrane-bound inhibitor homolog OS=Homo sapiens GN=BAMBI PE=1 SV=1 - [BAMBI_HUMAN] | 32.07 | 3.46 | 2.392344498 |
| O14545 | N-chimaerin OS=Homo sapiens GN=CHN1 PE=1 SV=3 - [CHIN_HUMAN] | 41.87 | 6.54 | 2.392344498 |
| Q9NRF8 | Myeloid-associated differentiation marker OS=Homo sapiens GN=MYADM PE=1 SV=2 - [MYADM_HUMAN] | 96.95 | 9.01 | 2.386634845 |
| Q15750 | Ankyrin repeat and SAM domain-containing protein 4B OS=Homo sapiens GN=ANKS4B PE=1 SV=2 - [ANS4B_HUMAN] | 33.63 | 5.04 | 2.386634845 |
| Q9BXJ1 | Mucin-13 OS=Homo sapiens GN=MUC13 PE=1 SV=3 - [MUC13_HUMAN] | 151.3 | 13.48 | 2.380952381 |
| P62979 | ERO1-like protein alpha OS=Homo sapiens GN=ERO1A PE=1 SV=2 - [ERO1A_HUMAN] | 916.95 | 43.16 | 2.375296912 |
| Q5MNZ9 | Synapse-associated protein 1 OS=Homo sapiens GN=SYAP1 PE=1 SV=1 - [SYAP1_HUMAN] | 1086.47 | 57.95 | 2.369668246 |
| P13645 | Protein FAM185A OS=Homo sapiens GN=FAM185A PE=2 SV=3 - [F185A_HUMAN] | 0 | 2.04 | 2.369668246 |
| Q9Y2D5 | Mothers against decapentaplegic homolog 3 OS=Homo sapiens GN=SMAD3 PE=1 SV=1 - [SMAD3_HUMAN] | 102.25 | 16.94 | 2.369668246 |
| Q68CJ9 | Beta-arrestin-2 OS=Homo sapiens GN=ARRB2 PE=1 SV=2 - [ARRB2_HUMAN] | 28.99 | 6.11 | 2.352941176 |
| P63208 | Immunoglobulin superfamily member 8 OS=Homo sapiens GN=IGSF8 PE=1 SV=1 - [IGSF8_HUMAN] | 42 | 3.43 | 2.341920375 |
| O76039 | Deoxyribonuclease-1-like 1 OS=Homo sapiens GN=DNASE1L1 PE=1 SV=1 - [DNSL1_HUMAN] | 35.86 | 2.98 | 2.341920375 |
| Q5U3C3 | Ribosomal protein S6 kinase beta-1 OS=Homo sapiens GN=RPS6KB1 PE=1 SV=2 - [KS6B1_HUMAN] | 27.86 | 2.29 | 2.336448598 |
| O43462 | Cytochrome P450 2S1 OS=Homo sapiens GN=CYP2S1 PE=1 SV=2 - [CP2S1_HUMAN] | 338.04 | 21.03 | 2.325581395 |
| Q15329 | Mothers against decapentaplegic homolog 4 OS=Homo sapiens GN=SMAD4 PE=1 SV=1 - [SMAD4_HUMAN] | 143.71 | 14.31 | 2.325581395 |
| P02751 | Antithrombin-III OS=Homo sapiens GN=SERPINC1 PE=1 SV=1 - [ANT3_HUMAN] | 0 | 4.74 | 2.320185615 |
| Q8N3E9 | Inositol 1,4,5-trisphosphate receptor type 3 OS=Homo sapiens GN=ITPR3 PE=1 SV=2 - [ITPR3_HUMAN] | 365.44 | 11.16 | 2.309468822 |
| Q8IUE0 | Aldehyde dehydrogenase X, mitochondrial OS=Homo sapiens GN=ALDH1B1 PE=1 SV=3 - [AL1B1_HUMAN] | 491.77 | 23.21 | 2.309468822 |
| P10599 | Tumor necrosis factor receptor superfamily member 5 OS=Homo sapiens GN=CD40 PE=1 SV=1 - [TNR5_HUMAN] | 29.29 | 3.61 | 2.309468822 |
| Q96CP6 | Thymosin beta-10 OS=Homo sapiens GN=TMSB10 PE=1 SV=2 - [TYB10_HUMAN] | 175.18 | 77.27 | 2.304147465 |
| O14668 | Lipopolysaccharide-responsive and beige-like anchor protein OS=Homo sapiens GN=LRBA PE=1 SV=4 - [LRBA_HUMAN] | 47.17 | 1.26 | 2.293577982 |
| Q9UL12 | Glutathione S-transferase kappa 1 OS=Homo sapiens GN=GSTK1 PE=1 SV=3 - [GSTK1_HUMAN] | 366.87 | 39.82 | 2.283105023 |
| P23229 | Gamma-enolase OS=Homo sapiens GN=ENO2 PE=1 SV=3 - [ENOG_HUMAN] | 3397.78 | 11.98 | 2.283105023 |
| P06396 | Persulfide dioxygenase ETHE1, mitochondrial OS=Homo sapiens GN=ETHE1 PE=1 SV=2 - [ETHE1_HUMAN] | 175.27 | 24.02 | 2.283105023 |
| P16144 | Tripartite motif-containing protein 6 OS=Homo sapiens GN=TRIM6 PE=1 SV=1 - [TRIM6_HUMAN] | 40.2 | 2.05 | 2.272727273 |
| Q5M7Z0 | E3 ubiquitin-protein ligase RBX1 OS=Homo sapiens GN=RBX1 PE=1 SV=1 - [RBX1_HUMAN] | 341.89 | 29.63 | 2.257336343 |
| Q96JQ2 | Solute carrier family 12 member 2 OS=Homo sapiens GN=SLC12A2 PE=1 SV=1 - [S12A2_HUMAN] | 1173.41 | 20.05 | 2.257336343 |
| Q09666 | Alcohol dehydrogenase 1B OS=Homo sapiens GN=ADH1B PE=1 SV=2 - [ADH1B_HUMAN] | 27.32 | 6.67 | 2.257336343 |
| Q9HCY8 | DNA polymerase subunit gamma-2, mitochondrial OS=Homo sapiens GN=POLG2 PE=1 SV=1 - [DPOG2_HUMAN] | 33.16 | 2.47 | 2.252252252 |
| P62987 | Enhancer of filamentation 1 OS=Homo sapiens GN=NEDD9 PE=1 SV=1 - [CASL_HUMAN] | 27.57 | 3.24 | 2.252252252 |
| Q9H361 | Spermine synthase OS=Homo sapiens GN=SMS PE=1 SV=2 - [SPSY_HUMAN] | 251.57 | 34.97 | 2.252252252 |
| P00734 | Epithelial cell adhesion molecule OS=Homo sapiens GN=EPCAM PE=1 SV=2 - [EPCAM_HUMAN] | 604.22 | 31.5 | 2.232142857 |
| P30613 | [Pyruvate dehydrogenase (acetyl-transferring)] kinase isozyme 3, mitochondrial OS=Homo sapiens GN=PDK3 PE=1 SV=1 - [PDK3_HUMAN] | 209.03 | 20.44 | 2.222222222 |
| O43157 | Inter-alpha-trypsin inhibitor heavy chain H2 OS=Homo sapiens GN=ITIH2 PE=1 SV=2 - [ITIH2_HUMAN] | 116.61 | 8.67 | 2.2172949 |
| P09936 | Uncharacterized protein C2orf72 OS=Homo sapiens GN=C2orf72 PE=1 SV=2 - [CB072_HUMAN] | 161.29 | 20.34 | 2.212389381 |
| P11168 | Zinc finger FYVE domain-containing protein 16 OS=Homo sapiens GN=ZFYVE16 PE=1 SV=3 - [ZFY16_HUMAN] | 185.99 | 12.22 | 2.207505519 |
| Q8IY57 | Scavenger receptor class B member 1 OS=Homo sapiens GN=SCARB1 PE=1 SV=1 - [SCRB1_HUMAN] | 206.28 | 11.05 | 2.202643172 |
| Q8TER5 | G protein-regulated inducer of neurite outgrowth 3 OS=Homo sapiens GN=GPRIN3 PE=2 SV=2 - [GRIN3_HUMAN] | 204.8 | 11.21 | 2.192982456 |
| Q2QL34 | Tubulin beta-2A chain OS=Homo sapiens GN=TUBB2A PE=1 SV=1 - [TBB2A_HUMAN] | 5810.27 | 52.36 | 2.188183807 |
| Q6PCB0 | Protein kinase C alpha type OS=Homo sapiens GN=PRKCA PE=1 SV=4 - [KPCA_HUMAN] | 172.57 | 19.64 | 2.188183807 |
| Q9NZJ6 | Mannosyl-oligosaccharide 1,2-alpha-mannosidase IA OS=Homo sapiens GN=MAN1A1 PE=1 SV=3 - [MA1A1_HUMAN] | 269.79 | 17.46 | 2.183406114 |
| P27701 | Coagulation factor V OS=Homo sapiens GN=F5 PE=1 SV=4 - [FA5_HUMAN] | 88.82 | 0.58 | 2.173913043 |
| Q96JB3 | Leukocyte receptor cluster member 9 OS=Homo sapiens GN=LENG9 PE=2 SV=2 - [LENG9_HUMAN] | 23.97 | 1.8 | 2.169197397 |
| Q8IVF7 | Sorcin OS=Homo sapiens GN=SRI PE=1 SV=1 - [SORCN_HUMAN] | 437.12 | 40.91 | 2.164502165 |
| P08709 | Transforming growth factor-beta receptor-associated protein 1 OS=Homo sapiens GN=TGFBRAP1 PE=1 SV=1 - [TGFA1_HUMAN] | 77.22 | 2.67 | 2.159827214 |
| Q8WW01 | Calcium/calmodulin-dependent protein kinase kinase 1 OS=Homo sapiens GN=CAMKK1 PE=1 SV=2 - [KKCC1_HUMAN] | 54.24 | 2.18 | 2.159827214 |
| P02652 | FERM domain-containing protein 4A OS=Homo sapiens GN=FRMD4A PE=1 SV=3 - [FRM4A_HUMAN] | 38.85 | 2.6 | 2.155172414 |
| P48681 | Paraneoplastic antigen-like protein 8A OS=Homo sapiens GN=PNMA8A PE=1 SV=2 - [PNM8A_HUMAN] | 51.09 | 2.96 | 2.155172414 |
| O60547 | ADP/ATP translocase 3 OS=Homo sapiens GN=SLC25A6 PE=1 SV=4 - [ADT3_HUMAN] | 1147.04 | 46.64 | 2.150537634 |
| O75414 | Coagulation factor X OS=Homo sapiens GN=F10 PE=1 SV=2 - [FA10_HUMAN] | 66.64 | 6.97 | 2.150537634 |
| Q96FQ6 | Peptidyl-prolyl cis-trans isomerase FKBP7 OS=Homo sapiens GN=FKBP7 PE=1 SV=1 - [FKBP7_HUMAN] | 104.7 | 19.69 | 2.145922747 |
| P07306 | CREB-binding protein OS=Homo sapiens GN=CREBBP PE=1 SV=3 - [CBP_HUMAN] | 99.35 | 2.83 | 2.145922747 |
| Q5TBA9 | Insulin receptor substrate 2 OS=Homo sapiens GN=IRS2 PE=1 SV=2 - [IRS2_HUMAN] | 32.57 | 1.12 | 2.141327623 |
| P46059 | Sequestosome-1 OS=Homo sapiens GN=SQSTM1 PE=1 SV=1 - [SQSTM_HUMAN] | 751.02 | 38.41 | 2.141327623 |
| Q12974 | 6-phosphogluconate dehydrogenase, decarboxylating OS=Homo sapiens GN=PGD PE=1 SV=3 - [6PGD_HUMAN] | 2004.58 | 39.54 | 2.141327623 |
| P25815 | Protein disulfide-isomerase A4 OS=Homo sapiens GN=PDIA4 PE=1 SV=2 - [PDIA4_HUMAN] | 5967.72 | 64.03 | 2.141327623 |
| P51589 | Reticulocalbin-3 OS=Homo sapiens GN=RCN3 PE=1 SV=1 - [RCN3_HUMAN] | 586.77 | 30.79 | 2.132196162 |
| Q5BJH2 | Semaphorin-3D OS=Homo sapiens GN=SEMA3D PE=2 SV=2 - [SEM3D_HUMAN] | 28.33 | 1.42 | 2.127659574 |
| P01009 | Glycerol-3-phosphate dehydrogenase, mitochondrial OS=Homo sapiens GN=GPD2 PE=1 SV=3 - [GPDM_HUMAN] | 884.62 | 42.64 | 2.123142251 |
| Q96EH3 | Ras-related protein Rab-3B OS=Homo sapiens GN=RAB3B PE=1 SV=2 - [RAB3B_HUMAN] | 107.92 | 31.05 | 2.123142251 |
| P04908 | Transgelin-2 OS=Homo sapiens GN=TAGLN2 PE=1 SV=3 - [TAGL2_HUMAN] | 1795.24 | 78.39 | 2.118644068 |
| Q6UX53 | Zyxin OS=Homo sapiens GN=ZYX PE=1 SV=1 - [ZYX_HUMAN] | 500.81 | 27.8 | 2.109704641 |
| Q93099 | Sterol 26-hydroxylase, mitochondrial OS=Homo sapiens GN=CYP27A1 PE=1 SV=1 - [CP27A_HUMAN] | 69.15 | 7.72 | 2.109704641 |
| Q8IVI9 | Beta-arrestin-1 OS=Homo sapiens GN=ARRB1 PE=1 SV=2 - [ARRB1_HUMAN] | 193.85 | 35.17 | 2.105263158 |
| Q9NX18 | Cell growth regulator with EF hand domain protein 1 OS=Homo sapiens GN=CGREF1 PE=2 SV=2 - [CGRE1_HUMAN] | 98.75 | 26.91 | 2.100840336 |
| Q8NBJ4 | Ras association domain-containing protein 6 OS=Homo sapiens GN=RASSF6 PE=1 SV=1 - [RASF6_HUMAN] | 22.11 | 2.17 | 2.100840336 |
| P69905 | Syntaxin-7 OS=Homo sapiens GN=STX7 PE=1 SV=4 - [STX7_HUMAN] | 611.05 | 49.81 | 2.096436059 |
| Q9UBF6 | Phytanoyl-CoA dioxygenase, peroxisomal OS=Homo sapiens GN=PHYH PE=1 SV=1 - [PAHX_HUMAN] | 102.48 | 22.49 | 2.096436059 |
| Q8NCU8 | Band 4.1-like protein 1 OS=Homo sapiens GN=EPB41L1 PE=1 SV=2 - [E41L1_HUMAN] | 55.53 | 10.22 | 2.092050209 |
| Q8N0U4 | Growth factor receptor-bound protein 14 OS=Homo sapiens GN=GRB14 PE=1 SV=2 - [GRB14_HUMAN] | 33.18 | 1.67 | 2.083333333 |
| P13284 | Aspartyl/asparaginyl beta-hydroxylase OS=Homo sapiens GN=ASPH PE=1 SV=3 - [ASPH_HUMAN] | 1161.28 | 39.84 | 2.079002079 |
| Q92597 | Neutral amino acid transporter B(0) OS=Homo sapiens GN=SLC1A5 PE=1 SV=2 - [AAAT_HUMAN] | 473.33 | 13.31 | 2.074688797 |
| O95807 | E3 ubiquitin-protein ligase TRIM4 OS=Homo sapiens GN=TRIM4 PE=1 SV=2 - [TRIM4_HUMAN] | 29.93 | 1.2 | 2.066115702 |
| Q8NC06 | Zinc finger FYVE domain-containing protein 9 OS=Homo sapiens GN=ZFYVE9 PE=1 SV=2 - [ZFYV9_HUMAN] | 35.52 | 1.05 | 2.06185567 |
| P13929 | Zinc finger and BTB domain-containing protein 24 OS=Homo sapiens GN=ZBTB24 PE=1 SV=2 - [ZBT24_HUMAN] | 39.58 | 1.43 | 2.06185567 |
| Q5HYK3 | TRAF-type zinc finger domain-containing protein 1 OS=Homo sapiens GN=TRAFD1 PE=1 SV=1 - [TRAD1_HUMAN] | 48.17 | 2.23 | 2.06185567 |
| Q9Y597 | TGF-beta-activated kinase 1 and MAP3K7-binding protein 1 OS=Homo sapiens GN=TAB1 PE=1 SV=1 - [TAB1_HUMAN] | 136.01 | 18.25 | 2.057613169 |
| Q9Y5T4 | CTP synthase 2 OS=Homo sapiens GN=CTPS2 PE=1 SV=1 - [PYRG2_HUMAN] | 663.67 | 24.23 | 2.05338809 |
| Q96GC9 | Complement C1q tumor necrosis factor-related protein 1 OS=Homo sapiens GN=C1QTNF1 PE=1 SV=1 - [C1QT1_HUMAN] | 35.88 | 2.49 | 2.049180328 |
| Q9NVV5 | Ubiquitin-40S ribosomal protein S27a OS=Homo sapiens GN=RPS27A PE=1 SV=2 - [RS27A_HUMAN] | 3411.4 | 54.49 | 2.044989775 |
| Q6PFW1 | A-kinase anchor protein 2 OS=Homo sapiens GN=AKAP2 PE=1 SV=3 - [AKAP2_HUMAN] | 50.46 | 6.87 | 2.040816327 |
| P02042 | Keratin, type I cytoskeletal 10 OS=Homo sapiens GN=KRT10 PE=1 SV=6 - [K1C10_HUMAN] | 229.01 | 12.67 | 2.036659878 |
| Q9UBV7 | Cyclic AMP-responsive element-binding protein 3-like protein 3 OS=Homo sapiens GN=CREB3L3 PE=1 SV=2 - [CR3L3_HUMAN] | 36.48 | 9.54 | 2.028397566 |
| P63261 | WD repeat domain phosphoinositide-interacting protein 1 OS=Homo sapiens GN=WIPI1 PE=1 SV=3 - [WIPI1_HUMAN] | 36.48 | 5.16 | 2.028397566 |
| Q9Y6H1 | Cyclin-dependent kinase-like 5 OS=Homo sapiens GN=CDKL5 PE=1 SV=1 - [CDKL5_HUMAN] | 93.75 | 2.33 | 2.024291498 |
| Q9BQL6 | Transmembrane protein 164 OS=Homo sapiens GN=TMEM164 PE=2 SV=1 - [TM164_HUMAN] | 25.12 | 10.44 | 2.02020202 |
| O75808 | S-phase kinase-associated protein 1 OS=Homo sapiens GN=SKP1 PE=1 SV=2 - [SKP1_HUMAN] | 547.49 | 51.53 | 2.02020202 |
| P19883 | Transcription factor E2F5 OS=Homo sapiens GN=E2F5 PE=1 SV=1 - [E2F5_HUMAN] | 31.87 | 4.34 | 2.016129032 |
| Q15149 | GRAM domain-containing protein 1A OS=Homo sapiens GN=GRAMD1A PE=1 SV=2 - [GRM1A_HUMAN] | 27.47 | 3.45 | 2.008032129 |
| Q8TD30 | 1-phosphatidylinositol 4,5-bisphosphate phosphodiesterase delta-3 OS=Homo sapiens GN=PLCD3 PE=1 SV=3 - [PLCD3_HUMAN] | 88.63 | 6.97 | 2.008032129 |
| Q8NBK3 | Homeobox protein TGIF2LY OS=Homo sapiens GN=TGIF2LY PE=1 SV=1 - [TF2LY_HUMAN] | 24.81 | 15.68 | 2.008032129 |
| P16403 | Thioredoxin OS=Homo sapiens GN=TXN PE=1 SV=3 - [THIO_HUMAN] | 445.26 | 31.43 | 2.008032129 |
| Q9H4K7 | Fibronectin OS=Homo sapiens GN=FN1 PE=1 SV=4 - [FINC_HUMAN] | 696.99 | 32.69 | 2.008032129 |
| Q9Y2Z9 | Membrane-bound transcription factor site-2 protease OS=Homo sapiens GN=MBTPS2 PE=1 SV=1 - [MBTP2_HUMAN] | 25.14 | 2.7 | 2.008032129 |
| P41235 | Gelsolin OS=Homo sapiens GN=GSN PE=1 SV=1 - [GELS_HUMAN] | 519.1 | 26.47 | 2.004008016 |
| Q969H0 | Transmembrane gamma-carboxyglutamic acid protein 1 OS=Homo sapiens GN=PRRG1 PE=1 SV=1 - [TMG1_HUMAN] | 27.46 | 3.67 | 2.004008016 |
| P28332 | Sarcosine dehydrogenase, mitochondrial OS=Homo sapiens GN=SARDH PE=1 SV=1 - [SARDH_HUMAN] | 35.38 | 2.51 | 2 |
| O43688 | Integrin alpha-6 OS=Homo sapiens GN=ITGA6 PE=1 SV=5 - [ITA6_HUMAN] | 233.17 | 11.06 | 2 |
| P01019 | Integrin beta-4 OS=Homo sapiens GN=ITGB4 PE=1 SV=5 - [ITB4_HUMAN] | 126.82 | 6.09 | 1.992031873 |
| Q96GW9 | Neuroblast differentiation-associated protein AHNAK OS=Homo sapiens GN=AHNAK PE=1 SV=2 - [AHNK_HUMAN] | 6992.26 | 64.82 | 1.945525292 |
| O00584 | Calmin OS=Homo sapiens GN=CLMN PE=1 SV=1 - [CLMN_HUMAN] | 27.48 | 0.9 | 1.848428835 |
| Q9BWJ5 | E3 ubiquitin-protein ligase RNFT1 OS=Homo sapiens GN=RNFT1 PE=1 SV=2 - [RNFT1_HUMAN] | 34.22 | 2.07 | 1.841620626 |
| Q96A29 | Polyadenylate-binding protein 3 OS=Homo sapiens GN=PABPC3 PE=1 SV=2 - [PABP3_HUMAN] | 1174.15 | 26.78 | 1.834862385 |
| P16402 | Ubiquitin-60S ribosomal protein L40 OS=Homo sapiens GN=UBA52 PE=1 SV=2 - [RL40_HUMAN] | 3298.56 | 50.78 | 1.828153565 |
| P15529 | Protein S100-A14 OS=Homo sapiens GN=S100A14 PE=1 SV=1 - [S10AE_HUMAN] | 96.74 | 21.15 | 1.805054152 |
| Q8N1A0 | Prothrombin OS=Homo sapiens GN=F2 PE=1 SV=2 - [THRB_HUMAN] | 112.28 | 4.66 | 1.773049645 |
| Q8IVL6 | Pyruvate kinase PKLR OS=Homo sapiens GN=PKLR PE=1 SV=2 - [KPYR_HUMAN] | 167.59 | 3.14 | 1.751313485 |
| Q96RS0 | Plexin-B1 OS=Homo sapiens GN=PLXNB1 PE=1 SV=3 - [PLXB1_HUMAN] | 38.2 | 0.84 | 1.736111111 |
| P60709 | Ubiquitin carboxyl-terminal hydrolase isozyme L1 OS=Homo sapiens GN=UCHL1 PE=1 SV=2 - [UCHL1_HUMAN] | 1364.51 | 27.91 | 1.733102253 |
| Q9UHA2 | Solute carrier family 2, facilitated glucose transporter member 2 OS=Homo sapiens GN=SLC2A2 PE=1 SV=1 - [GTR2_HUMAN] | 116.72 | 11.07 | 1.715265866 |
| P50416 | YY1-associated factor 2 OS=Homo sapiens GN=YAF2 PE=1 SV=3 - [YAF2_HUMAN] | 153.51 | 20 | 1.709401709 |
| P52895 | Rho guanine nucleotide exchange factor 40 OS=Homo sapiens GN=ARHGEF40 PE=1 SV=3 - [ARH40_HUMAN] | 57.28 | 2.44 | 1.680672269 |
| P49441 | von Willebrand factor A domain-containing protein 1 OS=Homo sapiens GN=VWA1 PE=1 SV=1 - [VWA1_HUMAN] | 21.97 | 2.02 | 1.675041876 |
| P51648 | Mpv17-like protein OS=Homo sapiens GN=MPV17L PE=1 SV=1 - [MP17L_HUMAN] | 34.66 | 5.1 | 1.672240803 |
| P30047 | Ubiquinone biosynthesis O-methyltransferase, mitochondrial OS=Homo sapiens GN=COQ3 PE=1 SV=3 - [COQ3_HUMAN] | 48.82 | 8.4 | 1.652892562 |
| P0DPB5 | CD82 antigen OS=Homo sapiens GN=CD82 PE=1 SV=1 - [CD82_HUMAN] | 419.32 | 51.65 | 1.647446458 |
| Q9H2D1 | Hypermethylated in cancer 2 protein OS=Homo sapiens GN=HIC2 PE=1 SV=2 - [HIC2_HUMAN] | 44.52 | 2.28 | 1.644736842 |
| P26374 | Formin-like protein 3 OS=Homo sapiens GN=FMNL3 PE=1 SV=3 - [FMNL3_HUMAN] | 64.52 | 5.16 | 1.628664495 |
| Q9NPJ3 | tRNA-splicing endonuclease subunit Sen15 OS=Homo sapiens GN=TSEN15 PE=1 SV=1 - [SEN15_HUMAN] | 33.86 | 23.98 | 1.623376623 |
| Q9BVL4 | Coagulation factor VII OS=Homo sapiens GN=F7 PE=1 SV=1 - [FA7_HUMAN] | 0 | 3.65 | 1.620745543 |
| Q01167 | Nestin OS=Homo sapiens GN=NES PE=1 SV=2 - [NEST_HUMAN] | 208.8 | 13.7 | 1.602564103 |
| Q8N5V2 | Apolipoprotein A-II OS=Homo sapiens GN=APOA2 PE=1 SV=1 - [APOA2_HUMAN] | 33.9 | 17 | 1.6 |
| O43513 | Nucleoside diphosphate kinase 6 OS=Homo sapiens GN=NME6 PE=1 SV=3 - [NDK6_HUMAN] | 22.18 | 6.99 | 1.592356688 |
| Q9UHE5 | Asialoglycoprotein receptor 1 OS=Homo sapiens GN=ASGR1 PE=1 SV=2 - [ASGR1_HUMAN] | 176.77 | 35.4 | 1.589825119 |
| P04035 | GDP-mannose 4,6 dehydratase OS=Homo sapiens GN=GMDS PE=1 SV=1 - [GMDS_HUMAN] | 357.53 | 58.33 | 1.589825119 |
| O95395 | Protein S100-A16 OS=Homo sapiens GN=S100A16 PE=1 SV=1 - [S10AG_HUMAN] | 383.69 | 42.72 | 1.587301587 |
| Q92508 | Protein tyrosine phosphatase type IVA 2 OS=Homo sapiens GN=PTP4A2 PE=1 SV=1 - [TP4A2_HUMAN] | 73.88 | 31.74 | 1.57480315 |
| Q6P582 | Protein S100-P OS=Homo sapiens GN=S100P PE=1 SV=2 - [S100P_HUMAN] | 390.48 | 48.42 | 1.57480315 |
| A5YM69 | Solute carrier family 15 member 1 OS=Homo sapiens GN=SLC15A1 PE=2 SV=1 - [S15A1_HUMAN] | 41.18 | 1.84 | 1.569858713 |
| Q6ZNA5 | Protein furry homolog OS=Homo sapiens GN=FRY PE=1 SV=1 - [FRY_HUMAN] | 30.74 | 0.66 | 1.567398119 |
| Q16658 | Cytochrome P450 2J2 OS=Homo sapiens GN=CYP2J2 PE=1 SV=2 - [CP2J2_HUMAN] | 29.56 | 2.59 | 1.540832049 |
| Q9Y2H5 | Mitochondrial assembly of ribosomal large subunit protein 1 OS=Homo sapiens GN=MALSU1 PE=1 SV=1 - [MASU1_HUMAN] | 39.23 | 8.97 | 1.531393568 |
| Q8TE67 | Transmembrane protein 128 OS=Homo sapiens GN=TMEM128 PE=1 SV=1 - [TM128_HUMAN] | 114.65 | 16.36 | 1.531393568 |
| Q9UHK6 | Alpha-1-antitrypsin OS=Homo sapiens GN=SERPINA1 PE=1 SV=3 - [A1AT_HUMAN] | 385.86 | 43.3 | 1.526717557 |
| P05026 | Homogentisate 1,2-dioxygenase OS=Homo sapiens GN=HGD PE=1 SV=2 - [HGD_HUMAN] | 160.08 | 18.43 | 1.508295626 |
| Q8NBS9 | Histone H2A type 1-B/E OS=Homo sapiens GN=HIST1H2AB PE=1 SV=2 - [H2A1B_HUMAN] | 1378.74 | 28.46 | 1.508295626 |
| P19484 | Methyltransferase-like protein 7B OS=Homo sapiens GN=METTL7B PE=1 SV=2 - [MET7B_HUMAN] | 280.15 | 29.92 | 1.503759398 |
| Q8NBJ9 | Golgi membrane protein 1 OS=Homo sapiens GN=GOLM1 PE=1 SV=1 - [GOLM1_HUMAN] | 60.71 | 22.94 | 1.501501502 |
| P82970 | Nostrin OS=Homo sapiens GN=NOSTRIN PE=1 SV=2 - [NOSTN_HUMAN] | 44.51 | 4.74 | 1.501501502 |
